# Electroacupuncture treatment of primary dysmenorrhea: A randomized, participant-blinded, sham-controlled clinical trial protocol

**DOI:** 10.1371/journal.pone.0282541

**Published:** 2023-05-26

**Authors:** Xiao Xue, Xin Liu, Sian Pan, Juan Li, Shaohua Wang, Hanyu Yuan, Yu Liu, Zenghui Yue

**Affiliations:** 1 College of Acupuncture, Massage and Rehabilitation, Hunan University of Chinese Medicine, Changsha, China; 2 The First Affiliated Hospital, Department of Chinese Medicine, Hengyang Medical School, University of South China, Heng Yang, Hunan, China; University of Abuja Teaching Hospital, NIGERIA

## Abstract

**Background:**

Primary dysmenorrhea in women is a common and serious public health problem with psychological and physical effects. Painkillers have adverse effects, such as tolerance, addiction, irritation of the digestive tract, and liver and kidney damage. Electroacupuncture has been used as alternative therapy, although with no (non-anecdotal) evidence of effectiveness.

**Objective:**

This study aims to provide evidence for the feasibility and efficacy of electroacupuncture in the treatment of primary dysmenorrhea. Moreover, by observing changes in serum and urine metabolites, we will evaluate the putative mechanisms mediating electroacupuncture effects in primary dysmenorrhea.

**Methods:**

This multicenter, randomized, participant-blinded, sham-controlled clinical trial including 336 women with primary dysmenorrhea is being conducted at three hospital centers in China and consists of a 12-week treatment and a 3-month follow-up. Women will undergo electroacupuncture (n = 168) or sham acupuncture (n = 168), beginning 7 days before their menstruation, once per day, until menstruation. Each menstrual cycle equals one course of treatment, and we will evaluate a total of three courses of treatment. The primary outcome of interest is the change in visual analogue scale scores before and after treatment. The secondary outcomes include changes in the numeric rating scale, Cox Menstrual Symptom Scale, traditional Chinese medicine symptoms, the Self-Rating Anxiety Scale, Self-Rating Depression Scale, and 36-Item Short Form questionnaire scores, and a safety evaluation. Moreover, we will preliminarily investigate the metabolomics mechanism as a potential mediator of the association between electroacupuncture and primary dysmenorrhea symptomology.

**Discussion:**

We aim to find a suitable non-medicinal alternative for primary dysmenorrhea treatment to reduce reliance on non-steroidal anti-inflammatory drugs.

**Trial registration:**

Chinese Clinical Trial Registry: ChiCTR2100054234; http://www.chictr.org.cn/.

## Introduction

Primary dysmenorrhea (PDM) is diagnosed when spastic pain occurs during the menstrual cycle and organic genital lesions have been excluded in a differential diagnosis. In 2020, an article published in the *Journal of the American Medical Association* reported that PDM affects 50–90% of women worldwide, causing substantial impacts on work and quality of life [1]. Moreover, a recent study reported that hypertension during pregnancy is closely related to dysmenorrhea in early adulthood [2]. Therefore, the primary prevention of dysmenorrhea, as well as slowing the disease progression and developing effective and low risk treatment modalities, are priorities within modern medical research.

At present, the Western medicine approach to treating dysmenorrhea mainly includes non-steroidal drugs. More specifically, drugs commonly prescribed for this condition include ibuprofen and oral contraceptives. However, the failure rate of these drugs is as high as 25% due to contraindications or intolerance [3]. Moreover, these medications are associated with a high risk of adverse side effects. The long-term use of such drugs can cause liver, kidney, and digestive system disorders; inhibit ovulation; thin the endometrium; cause a series of adverse reproductive reactions (including impacts on menstrual volume), headache, and drowsiness; and even increase breast cancer risk [4].

In contrast, according to a recent meta-analysis, the effect of electroacupuncture (EA) on controlling PDM symptomology was statistically significant in both animal experiments and clinical epidemiologic investigations [5–8]. Moreover, EA is safe and effective and may therefore be an excellent candidate for an alternative therapy within Western medicine.

Whether EA can relieve pain in PDM is controversial. Thus, this multicenter study aims to develop a validated protocol for treating PDM symptomology by applying EA at specific acupoints using a sham acupuncture (SA) control group. The proposed study protocol describes a randomized, controlled, participant-blinded experiment. Specifically, the proposed study evaluates the efficacy of EA in alleviating PDM as well as the use of metabolomic techniques to reveal the associated effector mechanisms, discover potential biomarkers, and derive associated metabolic pathways. Through this research, we aim to explain the mechanisms mediating the effects of EA on PDM symptomology at the overall biological level.

The primary aim of our study is to add to the existing evidence base on the effects of EA treatment on PDM symptomology. Furthermore, we aim to find a suitable alternative to pharmaceutical treatment in patients with PDM to reduce reliance on non-steroidal anti-inflammatory drugs (NSAIDs).

## Materials and methods

### Objectives

The proposed study has three objectives: to evaluate the immediate, near-term, and long-term efficacy of EA in alleviating PDM symptoms, evaluate the safety of EA treatment, and evaluate the metabolic pathways that mediate these associations. Specifically, we aim to evaluate the changing patterns of endogenous small molecule metabolites in blood and urine samples, compare changes in metabolic profiles within a treatment and a sham group, elucidate the specific components underlying the hypothesized mediating pathways, and explore the possible biological mechanisms through which EA might affect PDM via hematuria metabolomics.

### Study design

We propose to conduct a multicenter, randomized, participant-blinded, sham-controlled clinical trial evaluating the effects of EA on PDM for which participants will be publicly recruited. All centers must screen cases in strict accordance with the specified diagnostic, inclusion, and exclusion criteria, as specified below. A total of 336 participants with PDM will be randomly assigned to two groups. The treatment group (n = 168) will receive EA, and the control group will receive SA (n = 168). The study consists of 12 weeks of treatment and 3 months of follow-up. Treatment will begin 7 days before each woman’s menstruation and will be applied once per day until menstruation starts. Each menstrual cycle equals one course of treatment. We will evaluate a total of three courses of treatment (i.e., three menstrual cycles) in the current study. A total of 21 treatments will be performed. The study will include the selected participants, implementing efficacy evaluators, and a statistician. With regard to SA, the enrolled participants will be blinded and evaluated blindly after the last treatment.

All participants must provide their written informed consent prior to enrollment. Written informed consent will be obtained from the parents or guardians of participants under 16 years of age. The operator responsible for the specific acupuncture treatment at each center must have an acupuncturist qualification certificate and agree to independently undertake each patient’s clinical treatment for at least 2 years. After group randomization, treatment will be arranged by an acupuncturist. The study acupuncturists at all three centers will be trained according to the same protocol to ensure consistency in treatment manipulation and modality. After the last treatment, the physician records will be reviewed to assess the fidelity of the treatment. The primary outcome evaluated in this study is the change in visual analogue scale (VAS) scores between the start and end of the study period. The secondary outcomes include changes in the numeric rating scale (NRS), Cox Menstrual Symptom Scale (CMSS), traditional Chinese medicine symptoms, the Self-Rating Anxiety Scale, Self-Rating Depression Scale, and 36-Item Short Form questionnaire scores, as well as a safety evaluation of the acupuncture intervention during the course of treatment and follow-up.

We also propose to collect blood and urine samples before and after treatment to evaluate changes in patterns in endogenous small-molecule metabolites detectable in blood and urine.

**Fig 1:**
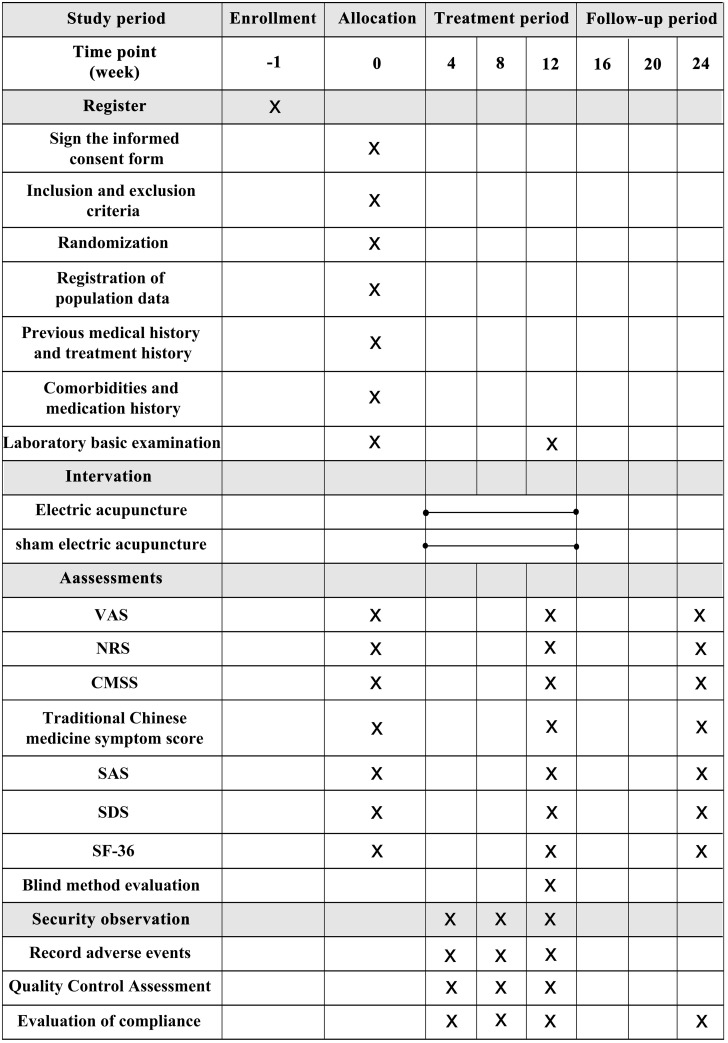
The SPIRIT schedule of enrollment, interventions, and assessment.

**Fig 2:**
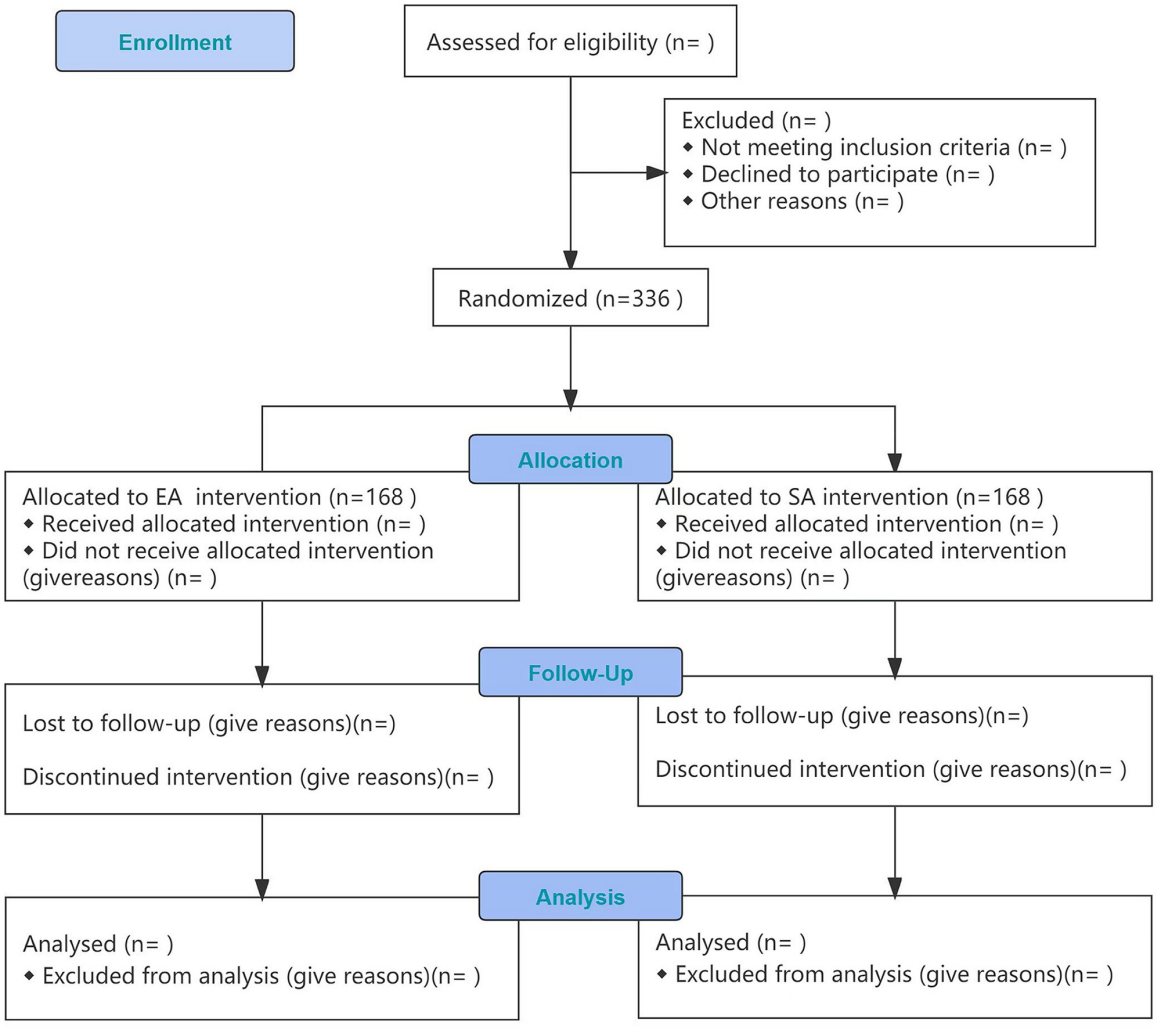
Study flow chart, in accordance with the CONSORT guidelines.

The study protocol, which was approved by the hospital ethics committee on December 9, 2021 (approval no: 2021KS-ZY-14-02), has been registered at www.clinicaltrials.gov (approval no: ChiCTR2100054234) and will follow the provisions of the Declaration of Helsinki and other relevant national and international guidelines. This protocol is conducted according to the 2013 Standard Protocol Items: Recommendations for Interventional Trials (SPIRIT) guidelines [9] (Fig 1, S1 Appendix). The intervention description follows the Template for Intervention Description and Replication (TIDieR) checklist and guide [10]. The study will be reported according to the Consolidated Standards of Reporting Trials (CONSORT) guidelines [11, 12] (Fig 2, S2 Appendix) and the Consolidated Criteria for Reporting Qualitative Research (COREQ) [13].

### Setting

This study will be spearheaded by the First Affiliated Hospital, Hengyang Medical School, University of South China as the leading unit. The sub-centers are the First Affiliated Hospital of Hunan University of Chinese Medicine and the Affiliated Changsha Central Hospital, Hengyang Medical School, University of South China. The three hospitals are affiliated with universities. An additional file shows the consent form detailing the risks and benefits of study enrollment that all participants will sign (S2 Appendix).

An additional file shows the case report form (CRF) in table format that we developed for the purpose of recording baseline medical and demographic data, various rating scale scores, and adverse reactions. An additional file shows the details of the Revised Standards for Reporting Interventions in Clinical Trials of Acupuncture (SPIRIT) checklist items (S1 Appendix).

### Recruitment

The study will be conducted by the University of South China (the central research headquarters). However, there are three centers participating in this project: the First Affiliated Hospital, Hengyang Medical School, University of South China; the First Affiliated Hospital of Hunan University of Traditional Chinese Medicine; and the Affiliated Changsha Central Hospital, Hengyang Medical School, University of South China. Participants will be publicly recruited according to three major strategies. The first strategy is to recruit participants from the three aforementioned hospitals. The second strategy is to print recruitment posters and distribute them across major health institutions and frequented public places to recruit potentially eligible research participants. The third strategy is to publish online advertisements introducing our study and recruiting volunteer participants.

### Inclusion criteria

Eligible participants must meet all of the criteria below to be considered for this study:

1. Diagnosed with PDM according to the International Classification of Diseases criteria (ICD-10-I63; Code: 902) [14].
2. Aged 16–35 years.
3. Regular menstrual cycles (defined as 28-day cycles ± 7 days) for 4–7 menstrual days.
4. Detailed contact information provided, with no short-term migration and ability to attend follow-up visits.
5. Received no treatments within the preceding month, including acupuncture, NSAIDs, anti-inflammatory drugs, and analgesics.
6. Willing to cooperate with the treatment procedures, medical examination, and efficacy evaluations, and must agree to not participate in other clinical experiments during the study period.
7. Provision of written informed consent prior to participation.
8. VAS score (i.e., the primary study endpoint described above) for three consecutive menstrual cycles ≥ 4 cm.

### Exclusion criteria

Participants were excluded for any one of the following:

1. Diagnosis of secondary dysmenorrhea (i.e., dysmenorrhea caused by uterine fibroids, adenomyosis, endometriosis, pelvic inflammation, internal foreign bodies, ovarian lesions, or other organic lesions).
2. Irregular menstrual periods.
3. History of thrombosis, embolism, cerebrovascular disease, coronary artery disease, or high risk for thrombosis.
4. Mental illness or cognitive impairment preventing comprehension of scale evaluation content.
5. History of complications, depression, or antidepressant medication use.
6. Severe heart, lung, liver, kidney, blood, immune, or endocrine system diseases.
7. Hematopoietic diseases, acquired immunodeficiency syndrome, tuberculosis, hepatitis, or miscellaneous infectious diseases (as differentiated from the specific infections defined above).
8. Malignancy, history of malignancy, or malignancy findings.
9. Pregnancy, planned pregnancy, or lactation within 1 year.
10. History of jaundice or herpes during pregnancy.
11. Participation in other clinical studies within 4 weeks before provision of informed consent.
12. Use of any of the following drugs within 4 weeks before provision of informed consent: gonadotropin-releasing hormone analogues or testosterone derivatives, hormone preparations containing mainly progesterone or estrogen, estrogen antagonists or aromatase inhibitors, and ongoing treatment for other gynecological diseases.
13. Metal allergy or severe needle phobia such that electroacupuncture treatment cannot be tolerated.
14. Skin rupture from needle, scars, or cardiac pacemaker.
15. Other lesions or conditions deemed inappropriate or too complicated according to the judgment of the researchers, such as a frequently changing work environment or unstable living conditions, which easily cause loss to follow-up.

### Screening

Patients are to be screened by research assistants and other trained study personnel. Study procedures include physical examinations, VAS administration, gynecological routine examinations, trans-abdominal B-ultrasound tests, and urinary pregnancy tests administered under the guidance of senior study personnel. We note that strict restriction regarding inclusion criteria is critical for controlling bias within the current study. Hence, we formulated clear inclusion and exclusion criteria to appropriately restrict research participants and reduce differences between participants. This is conducive to drawing objective conclusions about the observed factors.

### Random allocation

This study will use a PROC PLAN software environment for stochastic randomization. A third party produced a confidential dynamic randomization scheme using the Interactive Web Randomization System (Beijing LNKMED Tech Co., Ltd., Beijing, China, http://www.lnkmed.com/edc/a/sysIndex). The group assigned to each subject will be determined by a randomized file. Files will be randomly generated according to statistical units (SAS version 9.4, SAS Institute Inc., Cary, NC, USA). To prevent the prediction of participants’ grouping, the length of each group will be kept confidential (for example, if the number of individuals in a group is 4, and if three people in a group are known to be grouped for various reasons, then the grouping of the fourth person can obviously be predicted). When the appropriate district length is selected, a random number of 336 subjects will be generated for each of the three centers at a 1:1 ratio (this number shall not be reused) and will include the treatment assignments with corresponding serial numbers: 1001–1336, 2001–2336, and 3001–3336 (i.e., random coding table). After screening each eligible participant, the researchers at each center who did not participate in the treatment or evaluation will be asked by the research assistant (non-blind personnel) to log into the randomization system. According to the enrollment order of participants, the treatment they will receive is determined by the randomization file. All other individuals participating in the study—including patients, physicians, and outcome assessors—will be blinded to the randomization procedure. Further, participants and outcome assessors will also be blinded to treatment allocation. For participants who complete the composition work but do not receive treatment, the random number cannot be reassigned to another participant. The next enrolled subject will receive the next random number in order. To ensure that each subject will receive the same chance of trial conditions in this randomized controlled study, we will balance the influence of confounding factors, avoid trial bias caused by subjective and random arrangements, and provide a scientific basis for statistical analysis. Participants from the three centers who are eligible for enrollment will be randomly assigned to receive an electric or sham needle using a 1:1 ratio. The present study design has been demonstrated as the most effective method for avoiding selection bias. Specifically, after allocation within the LNKMED Interactive Web Randomization System by a research assistant who will not be participating in the treatment and evaluation procedures, basic patient information will be entered into the allocation system to enroll patients meeting the inclusion criteria into the randomization process and obtain the corresponding randomization numbers and groupings. After enrollment, the clinical researcher will record the demographic information, including age, race, marital status, education level, employment status, comorbidities, and body mass index, within the study data system. Moreover, the duration of dysmenorrhea, treatment history, and related scale scores will be recorded before treatment. The study protocol includes regular monitoring and bias evaluations among centers.

The random coding table (i.e., the grouping corresponding to the randomly generated numbers) will be sealed in an envelope and placed at the clinical trial institution for storage, as a “blind bottom.” This study follows a first-level blind design, in which the primary randomized number corresponds to the actual treatment group. The random coding table is generated by the statistical unit, and the blind bottom will be sealed separately and delivered to the responsible clinical trial unit at The First Affiliated Hospital, Department of Chinese Medicine, Hengyang Medical School, University of South China.

The central randomization system designates strict personnel authority. More specifically, no one other than the highest administrator is allowed to view the random scheme within the system, thereby ensuring a completely random distribution and effectively eliminating human interference and error.

The investigator will not perform emergency unblinding unless grouping information is required to medically treat the patient.

In this trial, the emergency unblinding process involves electronic emergency letters. When the investigator believes that the grouping information is required for the treatment of serious adverse events, emergency unblinding can be achieved through the LNKMED Interactive Web Randomization System. Meanwhile, the researcher will record the relevant data in detail, including the time of unblinding, cause, trial treatment, treatment, etc. Once the electronic emergency envelope is opened, the patient will be treated as a shedding case, that is, as a participant missing from the trial.

### Informed consent

The informed consent procedure is as follows. Before each participant is enrolled in the study, the investigator must comprehensively introduce the study background, purpose, inclusion and exclusion criteria, possible benefits and risks, participant rights and compensation, and obligations (as specified in the Declaration of Helsinki). After providing written informed consent, the participant or their guardian (if the participant is under the age of 16 years) will be provided a clinical study document (e.g., an informed consent document) for future reference. Participants receive free treatment and examination and some compensation for their participation in the study. Participants’ self-reported ethnicity information will routinely be collected without ethnic discrimination. The personal privacy and anonymity of the enrolled participants are to be kept strictly confidential, adhering to all relevant laws and regulators. Supervisors, inspectors, ethics committees, and administrative authorities shall be allowed direct access to the original medical records to verify procedures and examine data in accordance with applicable laws and regulations.

During the course of the study, the participant has the right to decide to withdraw from the study without any discrimination or retaliation. Withdrawal from the study will not affect the provision of any medical services. Personal privacy and data confidentiality will be protected during the study, and personal information will likewise be protected with regard to unauthorized contacts. Privacy and security procedures include removing identifying information from the main study database. During the trial, if new perspectives on research ethics arise, the informed consent documentation will be modified and resubmitted to the ethics committee. After approval, the participants will again be asked to sign the updated informed consent form. All informed consent forms should be kept as clinical study documentation for adherence to safety and ethics protocols and for future reference. We note that, after the end of the study, the results of this investigation will be published in a public venue regardless of obtaining negative or positive results.

### Acupoints

The acupuncture prescription evaluated in this study is as follows. First, the evaluated protocol was designed according to the findings of a systematic review [15]. Moreover, our research group searched the clinical literature relevant to acupuncture and PDM in established medical and scientific databases (PubMed, Web of Science, CNKI, Wanfang database, etc.) and subsequently constructed an acupoint compatibility database according to the results of the prior systematic review and our own literature search. We found that the two most highly evaluated acupoints were SP6 and CV4. Therefore, SP6 and CV4 were selected for the EA treatment of PDM in the current study.

### Intervention

#### EA group

**Fig 3:**
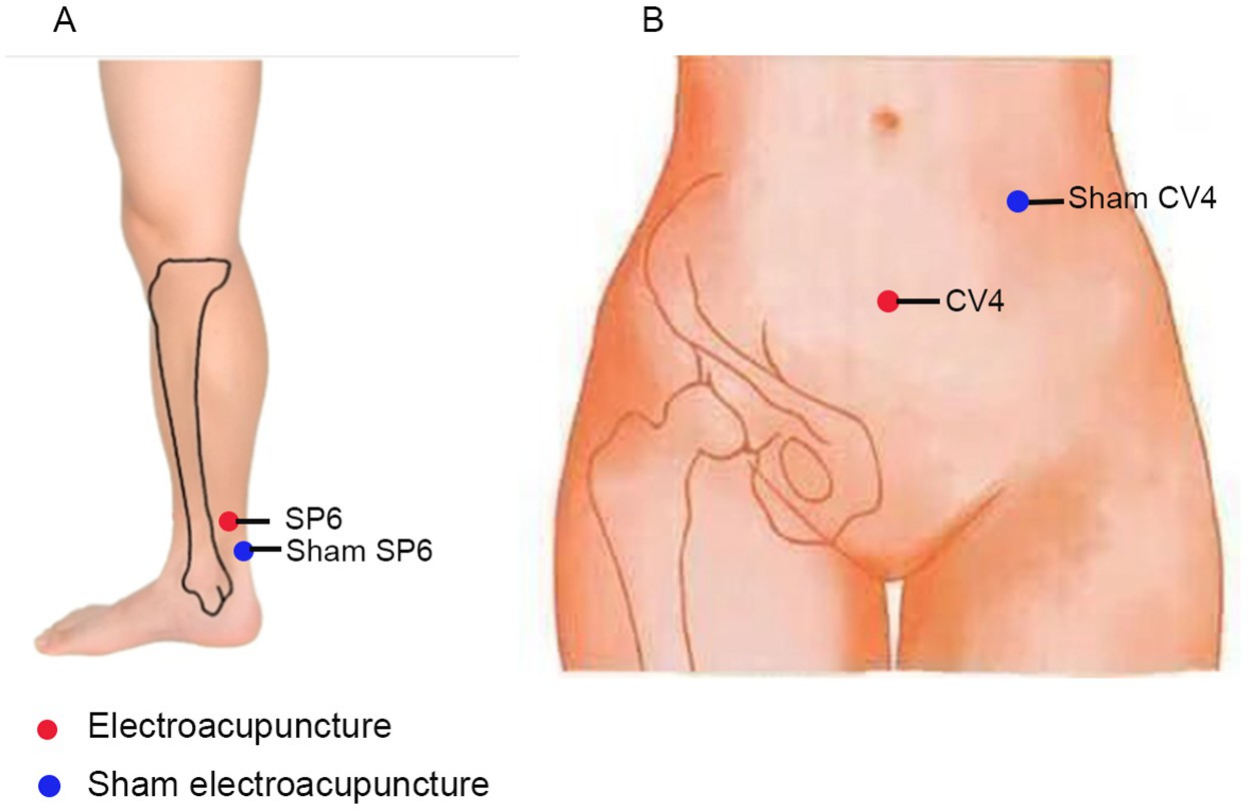
Acupuncture points used in this study.

Hwato brand disposable acupuncture needles (size 0.30 ×40 mm), and SDZ-V (Suzhou Medical Device Co., Ltd) electroacupuncture apparatuses will be used. Participants in the EA group will be treated at the following acupoints: bilateral sanyinjiao (SP6), located in the medial calf, 3 cun (approximately 60 mm) above the tip of the medial malleolus (Fig 3A) behind the medial edge of the tibia; and guanyuan (CV4), located in the lower abdomen on the anterior midline, 3 cun below the umbilicus (Fig 3B). The acupoints will be routinely disinfected prior to treatment. Acupuncture needles will be inserted 0.8–1.5 cun (approximately 16–30 mm) into the skin. Needle insertion will follow an angle of 30–45° in an inferomedial direction for SP6.

The acupuncture needle will be inserted vertically into CV4. Following needle insertion, small, equal manipulations of twirling, lifting, and thrusting will be performed on all needles to reach de qi (a composite of sensations including soreness, numbness, distention, heaviness, and other sensations believed to be an essential component for acupuncture efficacy). After de qi is achieved, paired electrodes on the electric needle device will be connected to the needle handle on one side of SP6 and CV4. Electroacupuncture stimulation will last for 30 min *a priori*, with a continuous wave of 100 Hz, and current intensities of 1–5 mA (the skin around the acupoints shakes slightly, which participants can typically tolerate without pain). We will administer EA 7 days before each menstrual cycle until menstruation is over. Each menstrual cycle equals one course of treatment. We will evaluate a total of three courses of treatment (i.e., three menstrual cycles) in the current study. A total of 21 treatments will be performed. During treatment, operators (i.e., operating personnel) will be separated from follow-up personnel. Before the electroacupuncture procedure, patients will be informed that they might experience slight pain after the treatment, caused by muscle spasms, which usually disappears within a period of several hours to 1 day without interventions and does not affect the next electroacupuncture treatment. If such pain occurs, the current intensity of the electroacupuncture shall be reduced in the next treatment session.

In addition to the unified training provided to all treating acupuncturists, we will also control for other influencing factors to avoid information bias such as randomization, blinding, and the objectivity of outcome indicators. Strict control of inclusion and exclusion criteria will limit the study participants to a specific range, reduce the differences between them, and enable us to draw objective conclusions about the observation factors.

#### SA group

Participants in the sham acupuncture group will receive sham electric acupuncture with false (sham) acupuncture points. The sham SP6 point is located 2 cun (approximately 40 mm) above taixi (KD3, midway between the spleen and kidney meridians [Fig 3A]). The sham CV4 point is located between the qihai (CV6) and yinjiao (CV7) levels, 3 cun lateral to the midline (Fig 3B). SA acupuncture needles will be inserted into the skin surface (2–3 mm). No acupuncture technique will be applied and de qi will not be attempted. The same electrode placements and other treatment settings will be used as in the EA group. However, the internal power cord will be cut off without any actual current output. In order to better implement blinding, all study patients will be treated separately to ensure that patients do not contact each other. Before the treatment, both groups will be told that they might not be able to feel the electrical stimulation due to body adaptability. Throughout the trial, participants will be treated individually with procedures in place to prevent communication between participants. To test for the blinding effect of the participants, all participants will be asked to guess whether they received an EA or a SA within 5 min of one session at week 12. The EA intervention methods used for participants in both groups are shown in Table 1.

**Table 1:** EA intervention methods used for participants in both groups.

**Etails of needling**	**Number of needles per session for each participant**	3 needles
	**Name and location of acupoints**: Refer to Name and Location of Acupoints 2006 (GB / T 12346–2006) and the International Code of Human Acupoints.	**EA group**: Sanyinjiao (SP6, double), Guanyuan (CV4) SP6: in the medial calf, 3 cun (approximately 60 mm) above the tip of the medial malleolus and behind the medial edge of the tibia. CV4: in the lower abdomen, on the anterior midline, 3 cun below the umbilicus.
		**SA group**: The sham SP6 point is located in 2 cun (approximately 40 mm) above taixi (KD3), midway between the spleen and kidney meridians, and the sham CV4 point is located between the levels of qihai (CV6) and yinjiao (CV7), 3 cun lateral to the midline.
	**Insertion depth**: Based on the developed unit of measurement or the specified level	**EA group**: Acupuncture needle inserts 0.8–1.5cun (approximately 16–30 mm) into the skin
		**SA group**: Acupuncture needle inserts into the surface of skin (2–3 mm)
	**Response**	**EA group**: De qi
		**SA group**: No de qi
	**Needle stimulation mode (electrical)**	**EA group**: The acupoints were electrically stimulated by an electric needle stimulator (model: SDZ-V), Selecting a continuous wave (frequency of 100 Hz).
		**SA group**: Electrode placement and other treatment settings were the same as in the electric needle group, although with no electrical output (the power cord was cut).
	**Keep needle time**	30 minutes
	**Type of needle**	**Disposable needles**: The specification is for φ 0.30 × 40 mm. That is, the thickness is 0.30 mm and the length is 1.5 cun, approximately 30 mm (Hwato brand disposable acupuncture needles; Suzhou Medical

EA: electroacupuncture, SA: sham acupuncture

### Handling of acupuncture accidents

The selected acupoints in this trial are not high-risk areas, which ensures the safety of the acupuncture procedure. Rare potential adverse events and their respective treatment are as follows: severe pain: quick injection and rest; subcutaneous hematoma: press the pinhole to stop bleeding, local cold compress, hot compress after 12 hours; and diapus: massage and side acupuncture.

### Emergency medication

In principle, no other treatments or drugs should be administered during the study period. However, if the participants experience painful menstrual periods that seriously affect their work and overall quality of life during the study period, they will be allowed to take ibuprofen sustained-release capsules (after explaining the situation to the researchers; Ibuprofen Sustained-release Capsules, approval number: H10900089, manufacturer: Sino-American Tianjin Shijiazhuang Pharmaceutical Co., Ltd., Shanghai, China). Researchers should specify the name, specification, dose, number of doses, and resolution of symptoms for any medications taken during the study period in the CRF, as this information will be used in the evaluation of the results.

### Primary outcome

**Fig 4:**
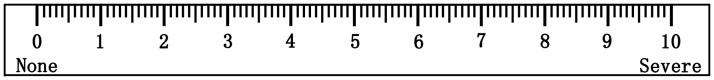
Visual Analogue Scale (VAS).

Participants will have been diagnosed with PDM according to the criteria specified in the International Classification of Diseases criteria (ICD-10-I63; Code: 902) [10]. Moreover, VAS scores will be recorded before and after treatment (i.e., during the follow-up period). We adopted a methodology based on the VAS card created by the Pain Society of the Chinese Medical Association, ranging from “no pain” (at 0 cm on the left side of the scale card) to “the most severe pain” (at 10 cm on the right side of the scale card). The distance from the starting point of the scale to the patient evaluation measurement is recorded in cm. The VAS score is measured by the patient placing the cursor at the area best representing their subjective level of pain at the time of evaluation. The score is recorded according to the position of the cursor (Fig 4).

### Secondary outcome

**Fig 5:**
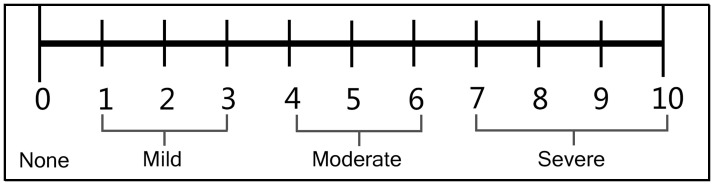
Numeric Rating Scale (NRS).

One of the secondary outcomes evaluated in this study is the change in NRS scores (Fig 5).

Pain level on this scale is indicated by 11 numbers ranging from 0 to 10 (0 = painless, 10 = the most painful). The degree classification standard was scored as follows: 0 = painless, 1 to 3 = mild pain, 4 to 6 = moderate pain, 7 to 10 = severe pain. The participants will be scored according to their individual pain sensations. This method is easily understood by patients and can be either dictated or written. The CMSS is a total frequency score indicating an average severity evaluation of menstrual dysmenorrhea and includes information on specific symptoms of menstrual dysmenorrhea. Moreover, a traditional Chinese medicine symptom score is formulated to score the evaluation of Chinese medicine symptoms according to the Guiding Principles for Clinical Research published by the Chinese Medical Science and Technology Press. The Self-Rating Anxiety Scale, Self-Rating Depression Scale, and 36-Item Short Form Survey are scales used to evaluate participants’ mood and quality of life during treatment and follow-up. A metabolomics test on blood and urine samples is to be used to evaluate metabolic product changes occurring after the treatment of PDM with EA and to provide findings informing the evidence base about putative mechanisms associated with metabolic pathways affected by EA.

### Laboratory indicators

#### Blood and urine metabolomics

All 336 enrolled participants will contribute blood and urine samples at baseline and on the second day of their week 12 menstrual period in the laboratory department of each hospital.

Blood collection: Blood will be taken from the cubital vein in the morning of the second day of menstruation (8:00–12:00), centrifuged 30 min followed by 3500 r/min for 10–15 min; the supernatant will be removed and saved at –80°C until testing (if precipitation occurs during the storage process, it will be centrifuged again).

Urine collection: Middle urine will be collected in the morning of each menstruation day and centrifuged at 12000 r/min for 10 min. The supernatant will be removed and saved at –80°C until testing (if precipitation occurs during storage, centrifugation will be repeated).

This project uses liquid mass combination (LC-MS) technology, which has no requirements for the volatility and thermal stability of measurement components, includes no tedious derivatization steps, follows a simple sample pre-processing procedure, has a low detection temperature, and provides fast and efficient separation, high sensitivity, and high specificity. It is therefore especially suitable for separation and high-throughput research and represents the most commonly used analysis technique in metabolomics research [16–20]. The Agilent6545, Agilent6550, and AB SCIEX 6600 systems will be used.

Screening of differential metabolites: The VIP value of a multivariate statistical model and the P value of a univariate analysis test will be used to screen for differential metabolites. Univariate statistical analysis methods, such as t-tests and analysis of variance, are more sensitive to independent changes in metabolite levels, while multivariate statistical analyses are better suited to reveal relationships between metabolites in biological process. Considering the results of both types of statistical analysis methods will help us observe the data from different angles and draw conclusions, and also help us avoid false positive errors or model over-fitting caused by using only one type of statistical analysis method. The screening threshold will be VIP > 1 and P < 0.05.

Database: A local self-built library (containing about 3000 metabolites; https://www.metware.cn/) and public databases including METLIN (https://metlin.scripps.edu/), the human metabolome database (HMDB; http://www.hmdb.ca/), the Kyoto encyclopedia of genes and genomes (KEGG; https://www.genome.jp/kegg/ligand.html), and other mainstream databases will be used.

#### Safety evaluation

Relevant safety indicators during treatment and follow-up are to be recorded by the acupuncturist on the participant adverse event record form. The EA-related safety evaluation includes assessments with regard to acupuncture fracture, dizziness symptoms, unbearable acupuncture pain (VAS ≥ 8 points), local hematoma, infections and abscesses, and other forms of discomfort following acupuncture (i.e., post-acupuncture pain, nausea, vomiting, diarrhea, palpitations, dizziness, headache, anorexia, or insomnia occurring for a duration of 1 hour or more). A specific record form is to be completed each time an enrolled patient undergoes diagnosis and treatment. Each patient is to be queried as to whether they experienced any of the above adverse events or symptoms. If so, this is to be immediately recorded in the CRF.

#### Participant blinding

This study is participant-blinded and will use independent treatment rooms and participant appointments to ensure that participants enrolled in the two study groups cannot communicate with each other. Both groups’ acupoint locations and EA operations are similar, thereby optimizing participant blinding. We will conduct evaluations of the blinding success rate within the EA and false EA groups, inform participants as to the presence of two EA therapies (EA with deep penetration and EA with shallow penetration), and ask participants whether they received deep penetration therapy (answer choices: yes, no, do not know).

Evaluations of the blind method are to be conducted within 5 min after the end of each course of treatment, as well as at the end of week 12 of the intervention period. Efficacy and safety indicators will be evaluated and recorded. Participants will also be followed up for efficacy at the end of week 24. Data management is to be undertaken by the data management department of Beijing Lingxun Pharmaceutical Technology Co, LTD, which will not participate in the experiment. The Electronic Data Capture System (EDC; https://www.lnkmed.com/edc/a/login) will be used for data acquisition. Data managers will design the characteristics of the EDC system and the actual requirements of the project to evaluate and record these outcomes through the data summary stage to ensure the accuracy and reliability of the research results.

We used the template from the SPIRIT 2013 Statement.

CMSS, Cox Menstrual Symptom Scale; NRS, Numeric Rating Scale; SAS, Self-Rating Anxiety Scale; SDS, Self-Rating Depression Scale; SF-36, 36-Item Short Form Survey; VAS, Visual Analogue Scale.

#### Sample size

The difference between the baseline VAS score and the VAS score at week 12 of the treatment is considered the main effect. Based on the evaluation of previous studies [21] as the basis for sample size estimation, after 60 days of treatment, we set the difference between the treatment groups to 4.75, the difference between control group and baseline to 0.29, the conservative estimate between the two groups to 2, and the standard deviation to 5 (α = 0.025 (one-sided), test efficacy 1–β = 0.9, superiority margin Δ = 0). According to the 1:1 ratio used in this study, at least 133 cases are required per group, at a lost-to-follow-up rate of 20%. The adjusted sample size is therefore 336, that is, 168 participants in each group.

### Statistical analysis

#### Descriptive statistics

SAS version 9.4 (SAS Institute) and STATA 14 (StataCorp) will be used for statistical analyses. The detailed statistical procedures and methods will be described in the separate Statistical Analysis document (the statistical analysis plan is a separate document, and the statistical analysis will be done before), which clearly formulates the hypothesis tests for the main and secondary indicators and describes the analysis methods (including the proposed fixed effects and covariates mentioned in the model), adjustments for missing data, and corrections for multiple comparisons.

#### General methods

Unless noted, all tests are performed at the two-sided 0.05-level, with 95% confidence intervals (CI). For continuous variables, descriptive statistics will include the means, interquartiles, standard deviations, and maximum and minimum values; for categorical variables, count data will be statistically described using frequencies (percentages).

#### Case enrollment and completion

The screening procedure, potential failures, random enrollment, and completion of the study will be recorded; the distribution of subjects at each study center and the final analysis sets will be summarized, as well as the main reasons for the participants’ withdrawal from the group and for data samples not being included in an analysis set.

#### Demographics and baseline analysis

Demographic information (participant age, sex, etc.) and baseline characteristics (e.g., history of disease) will be collected.

#### Combined drug use

Concomitant drugs will be recorded, including details on the drug name, reason of use, usage and dosage, use time, and any other relevant information.

#### Analysis of the primary efficacy indicators

The difference between baseline scores and VAS scores at week 12 of the primary index treatment will be tested as follows: H_0_: μ_T_ − μ_C_ ≤ Δ; H_1_: μ_T_ − μ_C_ > Δ; μ_T_: mean value of differences between baseline and 12-week treatment scores; μ_C_: mean value of the difference between baseline and 12-week treatment scores in the control group; Δ: excellent effect boundary value. The numbers of cases, means, standard deviations, medians, interquartiles, and minimum and maximum values will be calculated for the difference between baseline and 12-week treatment scores. This study will use a general linear model analysis, with the change value in the VAS score from baseline to week 12 as the dependent variable, the treatment as fixed effect, and the baseline score and center as covariates. If the number of single cases in a single group does not reach 5, a post-center analysis will be applied. If the lower limit of the 95% CI of the difference between the VAS score at week 12 in both groups is greater than the optimal threshold value, the test group can be considered superior to the control group.

#### Adjustments for missing data

The estimation of missing values for the main variables, such as no case data for the whole course of treatment, assumes that the data are missing at random (MAR), and multiple filling (MI) will be applied. The following methods will be used for the sensitivity analysis: 1) Assume that the deletion is a non-random deletion (MNAR), and fill in the missing data with the control group-based mixed model (PMM) for sensitivity analysis; 2) Assume that the VAS score of the control group at the 12th week is smaller than the baseline (this method is more conservative); 3) AssumingMNAR, the sensitivity analysis will be performed using a PMM based on the corresponding acupuncture test group; and 4) Assuming that the subject did not terminate the specific treatment early, which is a method that better reflects the baseline decrease in VAS scores at week 12. Missing data were not filled in for the sensitivity analysis.

#### Secondary efficacy index analysis

Secondary efficacy indicators will be analyzed using different statistical methods, depending on data distribution characteristics.

Differences in VAS scores, 4, 8, 12, 24, COX 4, 8, 12, 24, menstrual symptom scale (CMSS), 4, 8, 12, 24 anxiety (SAS), self-rating scale of depression (SDS), and quality of life scale (SF-36). Hypothesis test: H_0_: μ_T_ = μ_C_ H_1_: μ_T_ ≠ μ_C_; μ_T_: Relevant indicators of the test group, μ_C_ Control group index. The number of cases, mean, standard deviation, median, quartile, minimum, and maximum value will be calculated, and group comparisons will be conducted using the same method used for the primary efficacy measures. An MMRM analysis will also be conducted, with score change value as dependent variables, treatment and time as fixed effects, and baseline score and center as covariates; the model will simultaneously include the interaction term time * treatment.

An effective response will be defined as a reduction in the VAS score to 50% of the baseline period. Hypothesis test: H_0_: P_T_ = P_C_ H_1_: P_T_ ≠ P_C_; P_T_: Related indicators of test group, P_C_: related indicators of control group. Cases and percentages will be calculated. A logistics regression model analysis will also be performed, with the effective response rate as the dependent variable, treatment as a fixed effect, and the center as a covariate. If the number of single cases in a single group does not reach 5, a post-center analysis will be applied. RD values and their 95% CIs will be calculated between the groups.

Evaluation of acupuncture expectation and patient self-evaluation hypothesis test: H_0_: P_T_ = P_C_ H_1_: P_T_ ≠ P_C_. Cases and percentages will be calculated. Chi-square tests or exact probability tests will be applied.

#### Electro-needle blinded evaluation

Hypothesis test: H_0_: P_T_ = P_C_ H_1_: P_T_ ≠ P_C_. Cases and percentages will be calculated. Chi-square tests or exact probability tests will be applied. Bang’s index values and 95% confidence intervals will be calculated, with positive values indicating blind failure and negative values indicating blind success.

#### Safety analysis

Adverse events will be coded using the latest set of standard medical terms (MedDRA 23.1 or above). Treatment-emergent adverse events (TEAEs) are defined as adverse events that occurred or worsened after the study 1 intervention. The incidence of TEAEs, TEAEs, TESAEs related and unrelated to the study intervention will be analyzed by treatment group and in total. The incidence of TEAEs will be presented using the system organ class (SOC) and preferred term (PT).

#### Multiplicity correction problem

Only the difference between baseline VAS scores and VAS scores at week 12 of the treatment will be used as an indicator, without accounting for multiple groups or time points; therefore, only one major hypothesis test will be applied without correction for multiplicity. All tests are two-sided, and P values < 0.05 will be considered statistically significant.

#### Metabolomics analysis

The raw data will be converted to mzML format using the Proteo Wizard, sealed for extraction, aligned, and corrected for retention time. Peak areas will be corrected using the SVR method, and peaks with a missing rate > 50% per group sample will be filtered out, corrected, and the metabolite identification information will be obtained by searching the database. The statistical analysis (performed using R) will be divided into a univariate and a multivariate statistical analysis. The former will report Student’s t-tests and differences between multiple analysis methods, and the latter will include a principal component (PCA) analysis, partial least squares discrimination (PLS-DA), and orthogonal partial least squares discrimination (OPLS-DA).

The metabolomics sample may contain missing values for different reasons: a. the signal is not detectable; b. a detection error occurs, such as ion suppression or instrument instability; c. due to the algorithm limits of peak lifting, the low signal cannot be extracted from the background; and d. overlapping peaks cannot be deconvoluted. Missing values in tables are usually recorded in the form of null values or as “NA”. Data filtering is a common method in metabolomics analyses, based on the proportion of missing values within a sample or group. For example, removing more than 50% of the missing peaks in the QC sample, where a QC sample is a mixture of all the samples used to assess the stability of the instrument; or removing more than 80% of the missing values in the sample (missing values > 50% are usually filtered out). A data matrix with unfiltered missing values may affect subsequent algorithm calculations, will trigger anomalies, and therefore requires simulation filling. A simple method is to fill it with fixed values, means, medians, minimums or 1/2 minimum values; more complex procedures use machine learning algorithms such as the near algorithm (KNN) [22] and random forest (RF) [23] calculations as follows.

*KNN padding*. The principle of the KNN approach is to identify k samples, spatially similar or close, in the dataset. We use these "k" samples to estimate the values of the missing data points. Missing values for each sample were interpolated using the mean of the "k" neighborhood found in the dataset. The KNN algorithm is the most robust algorithm in the missing value filling method, which has been widely used in recent years. But some researchers believe in the need to choose according to the missing type, for completely nonrandom missing can use half of the minimum value of filling, completely missing or random missing using random forest method (Wei et al., 2018). As there is no completely unified standard, the specific filling method should be chosen according to its data type and biological significance.

*The RF method*. Random forests can also be applied to the regression problem, depending on whether each cart tree in the random forest is a classification or regression tree. It puts back and samples the original dataset many times, getting many different data sets, and then builds a decision tree for each dataset. The final result of the random forest is the average of the results of all trees. A new observation gets “n” predicted values from many trees (such as “n trees”), and the average of the n predicted values is filled as the final result. Of course, it is the same as the above regression data preprocessing process, first building the training set and prediction set before the model prediction.

The universal threshold for metabolome difference analysis, is not invariable. For the actual problem and actual analysis, such as before the omics analysis has been through experimental validation, some interest material is a real difference, but omics analysis may not achieve a significant difference standard. This situation is often encountered in data screening, and this time can according to the difference of the substances of interest standard to adjust the threshold, such as the FC value reduced to 1.5 times and etc.

#### Data collection and management

The clinical research team has developed a data safety monitoring plan according to risk determinations.

The PDM CRF has been designed to collect data for each participant individually. All clinical observations will be recorded in the CRF with timely input into the central database. The original CRF, informed consent form, clinical medical record, original laboratory sheet, and original data must be traceable for each participant. The assigned investigator must manually complete the RF, regularly collect the necessary quality control and safety evaluation information and use the double-double entry method for data entry. This project uses the EDC system to conduct data collection and the single input of data both by the system and manually. Electronic records shall be original, real-time, accurate, complete, reliable and traceable. All electronic records must be modified to ensure that the starting records and the records in the test process can be recovered; the recorded date and time shall be accurate to year, month, day, hours and minutes. The electronic signature shall be executed by a special person (the Principal Investigator of the study) as authorized by the research unit. The Clinical Research Associate (CRA) will check the original subject records, check the CRF with the original records, mark the doubt data, and ask the researcher to confirm or correct it. The project leader shall arrange for a third party to save and store this documentation. Access to the data will be limited to approved researchers on the center-specific research team.

This study will establish a Clinical Trial Data Monitoring Committee (DMC), which will consist of an acupuncturist, a gynecologist, statisticians, and an ethics committee. The DMC will support the study participants and monitor the safety, effectiveness, and quality of the study and will advise the sponsor whether to continue, adjust, or stop the study. The DMC will be unblinded to analyze, summarize, or check the experiment before data collection and will ensure confidentiality during data transmission.

The data will be stored in the database of the project (https://www.lnkmed.com/edc/a/edc-web/index.html. After the trial, the original data set can be exported directly by the system. The SAS programmer will generate the analysis data set through the original data set, and the statistician will generate the statistical results by analyzing the data set. Finally, the website submitted to the Ministry of Science and Technology of The First Affiliated Hospital, Department of Chinese Medicine, Hengyang Medical School, University of South China (https://www.nhfyyy.com/).

#### Data questioning processing

The Principal Investigator (PI) will log in to the EDC system, check the consistency of case report form and source data, and record the case report within the specified time. The researcher can answer the questions online or download the list of questions and answer offline. Then, the researcher input the questions into the EDC. The data administrator and supervisor can approve the researcher to enter and answer questions, and ask questions by separate CRA or medical roles.

#### Adverse incident monitoring

All adverse events shall be recorded in detail and handled and tracked until properly resolved or stable, and serious adverse events shall be reported to the ethics committee and competent authorities as required according to institutional regulations and relevant laws. The principal investigator shall conduct a cumulative review of all adverse events and hold an investigator meeting if necessary to reassess the risks and benefits of the study in light of the new information arising during the course of the study. Once an adverse event is identified, this event shall be followed-up until it is resolved or until the adverse event is determined to be permanent.

Moreover, researchers must assess changes in the severity of the adverse events occurring during the course of the study, suspicious reactions to the study treatment, and the need for intervention at each visit (including recommending increased study visit frequency if needed). All adverse events must be recorded on the adverse events page of the CRF. Adverse events must be recorded with regard to time, severity, and duration parameters, as well as with relevant information on measures and outcomes. Adverse events must be carefully and accurately recorded during the trial. Adverse events are to be divided into acupuncture-related and non-acupuncture-related events according to their potential reactions with the acupuncture treatment process.

#### Quality control

This study protocol specifies three levels of quality control inspections. A grade 1 quality inspection involves at least one non-treatment practitioner performing a relevant quality inspection. For level 2 quality control inspections, the group leader of the project at the First Affiliated Hospital, Hengyang Medical School, University of South China shall appoint at least three non-treatment implementers to conduct quality inspections. Level 3 quality control inspections are to be conducted by the Clinical Evaluation Center of the Hunan University of Traditional Chinese Medicine following the appointment of at least three quality control personnel for conducting quality inspections within this clinical trial. The research team will hold a special clinical training meeting before the official launch of the clinical trial to conduct unified training for the clinical researchers at each center. Discuss the possible issues with the local supervisor or the head of the clinical medicine department and get a unified understanding.

Acupuncturists must have the necessary qualification certificate and more than two years of work experience. Key training on the project implementation plan and standard operating procedure includes training on project implementation steps and methods; specifications regarding diagnoses, inclusion and exclusion criteria, evaluation and filling methods of the CRF, and each scale; and specifications regarding the acupuncture operation method. Each clinical researcher must be made familiar with the research process and specific implementation rules to improve internal observation consistency and interobserver consistency and to ensure the reliability of clinical research conclusions. An investigator statement must be signed to establish a standard operating procedure for experimental index observation as well as for conducting quality control evaluations in the laboratories of the participating hospitals. Quality control measures are to be implemented at each center, including conducting regular monitoring and controlling bias between research centers.

#### Trial monitoring

After recruitment and before randomization, all participants must undergo routine blood and urine tests as well as liver and kidney function tests to identify and exclude participants with severe heart, liver, and/or kidney disease. Participants will undergo these tests again at the end of the study period to assess the possible side effects of the intervention as well as possible adverse reactions to acupuncture, including acupuncture fractures, acupuncture misses, dizziness, local hematoma, infection, and abscess. Participants will also be assessed with regard to the incidence and average number of symptoms with respect to post-acupuncture pain, nausea, vomiting, diarrhea, palpitations, dizziness, headache, anorexia, and insomnia. A specific record table is to be constructed, including specific information on each participant’s diagnosis and treatment as well as queries as to whether any of the above situations have occurred. Any adverse events will be recorded in the CRF table in a timely manner.

#### Ethics and dissemination

The study has been approved conducted in accordance with the National Declaration and the Declaration of Helsinki and other relevant national and international guidelines be in progress, And it has been approved by the Clinical Research Group Committee of the First Affiliated Hospital of South China University (No.2021KS-ZY-14-02), The study has been registered at http://www.chictr.org.cn/ (approval no: ChiCTR2100054234), The information explains the study in great detail, Including the purpose of the study, the basic research content, the process, the methods, and the research time limit; the basic information of the researchers and the qualification of the research institution; the benefits that the research results may bring to the subjects, the relevant persons, and the society, And the possible discomfort and risks posed to subjects; protective measures for subjects; the scope and measures for the confidentiality of study data and subject personal data; subject rights, Including voluntary participation and withdrawal at any time, knowledge, consent or disapproval, confidentiality, compensation, obtaining free treatment and compensation when damage, access to new information, and obtaining informed consent; Precautions before, after and during the study The equal voluntary nature of participants and the choice to withdraw at any time. Participants were also given the confidentiality and secure data storage of their personal information., Any adverse events arising will be reported and managed by the instructors and the research team. Data will be securely stored EDC Systems and Test Initiation Center. No person shall have access to the collected data without the authorization of the project leader and the supervisor.

## Discussion

The pathogenesis of PDM, also known as “menstrual abdominal pain” and “moon water to abdominal pain,” is currently believed to involve endogenous factors such as immune activation and inflammatory cytokines [24]. Evidence indicates that PDM is also influenced by age, marital status, social environment, education level, stress, and other exogenous factors [25, 26]. The etiology of PDM is currently not well understood.

Modern medical treatment of PDM mainly consists of oral NSAIDs and contraceptives. NSAIDs are the first-line treatment for PDM worldwide. However, EA treatment for PDM may be a promising alternative therapy. The anti-inflammatory mechanisms of EA have previously been studied with regard to other inflammatory diseases [27, 28]. Mediation through elevated uterine prostaglandin levels is a generally accepted explanation for the pathogenesis of PDM [29]. Several studies have also investigated potential mechanisms through which EA may relieve PDM by reducing inflammatory factor levels, such as human prostaglandin F2α. However, multicenter, large-sample, randomized controlled trials and relevant mechanistic studies on this topic are lacking, and the specific mechanisms by which EA relieves PDM remain unclear. Therefore, we designed this multicenter, large-sample, SA-controlled, randomized clinical trial to evaluate participant scale scores to obtain high-quality research data to provide a reliable evidence base for the effectiveness of EA in PDM. Hematuria samples will be collected from the participants in both treatment groups before and after treatment. The hematuria samples will be processed and analyzed before and after treatment using metabolomics methodologies to identify potential biomarkers, derive information on related metabolic pathways, and clarify the potential mechanisms through which EA may positively affect PDM symptomology.

In this study, SA will be administered to the control group to eliminate any potential placebo effects of acupuncture. To help maximize participant blinding, we referred to the experimental designs of a large number of investigations that administered SA [30–32]. We found that designing a non-penetrating pseudo-acupuncture procedure that does not produce any physiological effects is difficult. This study limitation may therefore create performance bias. Hence, in this study, we designed an EA intervention that did not penetrate the skin or involve current output. However, our SA design is limited by reduced participant compliance coupled with the long implementation period and possibly a higher exit rate among the enrolled participants receiving SA (i.e., with potentially insufficient blinding).

We have established guidelines and training for researchers to improve participant compliance. Relevant measures include good communication with participants and higher subsidies. These issues were explained in detail in the ethical documentation for this research.

## Conclusions

Overall, the results of this trial are expected to provide clinical evidence regarding the effectiveness of EA for treating PDM symptomology and explain the possible mechanisms through which EA may affect PDM (namely, through metabolomic pathways). On the basis of exploring the effectiveness of EA in the treatment of PDM, we aim to preliminarily investigate the metabolomics mechanism as a potential mediator of the association between EA and PDM symptomology. Specifically, the proposed study aims to use metabolomic techniques to elucidate the associated effector mechanisms of EA, discover potential biomarkers, and derive associated metabolic pathways.

Our aim is to find a suitable non-pharmacological alternative for treating PDM to reduce reliance on NSAIDs. Our proposed protocol thus guides future research directions while directly adding to the evidence base for this alternative and complementary therapy according to the Western medicine paradigm.

## Supporting information

S1 Appendix SPIRIT checklist. (DOCX)

S2 Appendix CONSORT checklist. (PDF)

S3 Appendix Trial protocol for ethics application. (DOCX)

S1 File (PDF)
