# Supplementary material for: Electroacupuncture treatment of primary dysmenorrhea: A randomized, participant-blinded, sham-controlled clinical trial protocol
Source: PLoS One. 2023 May 26;18(5):e0282541. doi: 10.1371/journal.pone.0282541 (PMC10218736; doi:10.1371/journal.pone.0282541)
Supplement: S3 Appendix — (DOCX) [file pone.0282541.s003.docx]

**Trial protocol for ethics application**

| Synopsis |  |
| --- | --- |
| **Name of the Funding Source:** Youth Program of National Natural Science Foundation of China (No. 82004490) and the Innovation Platform Open Fund project of the Hunan Education Department (no.20K091) and the University of South China Clinical Research 4310 Program（no.20214310NHYCG04） | **Protocol Number:**  Version 2.0 |
| **Study Title:** Electroacupuncture treatment of primary dysmenorrhea: a randomized, participant-blinded, sham-controlled clinical trial | |
| **Short Title:** Electroacupuncture Treatment of Primary Dysmenorrhea | |
| **Planned Study Sites:** This study will be spearheaded by the First Affiliated Hospital, Hengyang Medical School, University of South China as the leading unit. The sub-centers are the First Affiliated Hospital of Hunan University of Chinese Medicine and the Affiliated Changsha Central Hospital, Hengyang Medical School, University of South China. The three hospitals are affiliated with universities. | |
| **Number of Participants:** A total of 336participants | |
| **Chief Investigator:**  Dr Xiao Xue | |
| **Study Period:** December 2022 – December 2025 | |
| **Objectives:** Primary dysmenorrhea is common among women, with both psychological and physical effects. Electroacupuncture has been used as alternative therapy for primary dysmenorrhea, although with no (non-anecdotal) evidence of effectiveness. This multicenter, randomized, participant-blinded, sham electroacupuncture-controlled clinical study will observe the prophylactic and therapeutic effects of electroacupuncture in participants with primary dysmenorrhea at three Chinese hospitals. Moreover, by observing changes in serum and urine metabolites before and after electroacupuncture, we evaluated the putative mechanisms mediating the electroacupuncture effects on patients with primary dysmenorrhea while focusing on the metabolic pathway. | |
| **Study Design:** This study is a randomized, participant-blinded, sham-controlled clinical trial trial. | |
| **Study Criteria:**  Inclusion criteria  Eligible participants must meet all of the criteria below to be considered for this study:  (1) Diagnosed with PDMM according to the International Classification of Diseases criteria (ICD-10-I63; Code: 902)  (2) Aged 16–35 years.  (3) Regular menstrual cycles (defined as 28-day cycles ± 7 days) for 4–7 menstrual days.  (4) Detailed contact information provided, with no short-term migration and ability to attend follow-up visits.  (5) Received no treatments within the preceding month, including acupuncture, NSAIDs, anti-inflammatory drugs, and analgesics.  (6) Willing to cooperate with the treatment procedures, medical examination, and efficacy evaluations, and must agree to not participate in other clinical experiments during the study period.  (7) Provision of written informed consent prior to participation.  (8) VAS score (i.e., the primary study endpoint described above) for three consecutive menstrual cycles ≥ 4 cm.  Exclusion criteria  Participants were excluded for any one of the following:  (1) Diagnosis of secondary dysmenorrhea (i.e., dysmenorrhea caused by uterine fibroids, adenomyosis, endometriosis, pelvic inflammation, internal foreign bodies, ovarian lesions, or other organic lesions).  (2) Irregular menstrual periods  (3) History of thrombosis, embolism, cerebrovascular disease, coronary artery disease, or high risk for thrombosis.  (4) Mental illness or cognitive impairment preventing comprehension of scale evaluation content.  (5) History of complications, depression, or antidepressant medication use.  (6) Severe heart, lung, liver, kidney, blood, immune, or endocrine system diseases.  (7) Hematopoietic diseases, acquired immunodeficiency syndrome, tuberculosis, hepatitis, or miscellaneous infectious diseases (as differentiated from the specific infections defined above).  (8) Malignancy, history of malignancy, or malignancy findings.  (9) Pregnancy, planned pregnancy, or lactation within 1 year.  (10) History of jaundice or herpes during pregnancy.  (11) Participation in other clinical studies within 4 weeks before provision of informed consent.  (12) Use of any of the following drugs within 4 weeks before provision of informed consent: gonadotropin-releasing hormone analogues or testosterone derivatives, hormone preparations containing mainly progesterone or estrogen, estrogen antagonists or aromatase inhibitors, and ongoing treatment for other gynecological diseases.  (13) Metal allergy or severe needle phobia such that electroacupuncture treatment cannot be tolerated.  (14)Skin rupture from needle, scars, or cardiac pacemaker.  (15) Other lesions or conditions deemed inappropriate or too complicated according to the judgment of the researchers, such as frequently changing work environment or unstable living conditions, which easily cause loss to follow-up. | |
| **Sample Size:** Based on previousPDMM therapy intervention studies, n = 168 participants will be required per group (total n=336) for this study. | |

# **Investigators’ qualifications**

Professor Xin Liu, The Chief Investigator, has 30 years working experience in the field of health. He worked in China, Hunan Province famous Traditional Chinese medicine, the host of the National Natural Science Foundation of China, research direction: acupuncture and moxibustion for pain treatment and the prevention and treatment of respiratory diseases, the project leader of this project,He has several publications in the field of TCM.

Professor Zenghui Yue, China first-class construction discipline of traditional Chinese medicine-acupuncture and massage science direction leader. Head of national first-class professional acupuncture and massage major,doctoral supervisor.He is the person in charge of the First Affiliated Hospital of Hunan University of Traditional Chinese Medicine,He is mainly engaged in the research of the treatment mechanism of acupuncture and moxibustion, presided over 4 national self-treatment projects, has in-depth research on acupuncture analgesia, publishes many papers on the clinical research of acupuncture and acupuncture, and plays a guiding role in the whole implementation of the project.

Dr XiaoXue，The first affiliated hospital of south China university attending physician, a doctor of Hunan university of Chinese medicine, nearly ten years has been carrying out the clinical research of electroacupuncture treatment of primary dysmenorrhea, successively for the treatment of electroacupuncture primary dysmenorrhea for a lot of clinical research he basic experimental research, around the research in the field of series participant read a lot of literature, the ancient literature about acupuncture treatment of dysmenorrhea and hole rule for the arrangement and research. Explore the aging, quantity and effect relationship of electricity for primary dysmenorrhea and has several publications in the field of TCM. Xiao Xue will provide overall support and mentorship on this project.

Dr YuLiu, Associate Professor, School of Acupuncture and Massage, Hunan University of Traditional Chinese Medicine. Her area of expertise is needle pain research. Dr. Liu has published several peer-reviewed journals and conference papers and book chapters. Since 2010, she has also received multiple research grants from the National Government Agency, Industry and University Research Council since 2010.

# **Research Scope, Aims, Themes, Questions and Methods**

**Aims or Questions**

The primary aim of our study is to add to the existing evidence base on the effects of EA treatment on PDMM symptomology. Furthermore, we aim to find a suitable alternative to pharmaceutical treatment in patients with PDMM to reduce reliance on non-steroidal anti-inflammatory drugs (NSAIDs).

**Benefit of exploring these research questions**

At present, the Western medicine approach to treating dysmenorrhea mainly includes non-steroidal drugs. More specifically, drugs commonly prescribed for this condition include ibuprofen and oral contraceptives. However, the failure rate of these drugs is as high as 25% due to contraindications or intolerance. Moreover, these medications are associated with a high risk of adverse side effects. The long-term use of such drugs can cause liver, kidney, and digestive system disorders; inhibit ovulation; thin the endometrium; cause a series of adverse reproductive reactions (including impacts on menstrual volume), headache, and drowsiness; and even increase breast cancer risk.

In contrast, according to a recent meta-analysis, the effect of electroacupuncture (EA) on controlling PDMM symptomology was statistically significant in both animal experiments and clinical epidemiologic investigations . Moreover, EA serves as a safe and effective “green” therapy and therefore may be an excellent candidate as an alternative therapy within Western medicine.

**Outline the design of this research; its methods and details of the instruments to be used to collect the data, including psychometric properties if applicable.**

**Study design:**

We propose to conduct a multicenter, randomized, participant-blinded, sham-controlled clinical trial evaluating the effects of EA on PDMM in which participants will be publicly recruited. All centers must screen cases in strict accordance with the specified diagnostic, inclusion, and exclusion criteria, as specified below. A total of 336 participants with PDMM will be randomly assigned to two groups. The treatment group (n = 168) will receive EA, and the control group will receive SA (n = 168). The study will include the participants, implementing efficacy evaluators, and a data statistician. With regard to SA, the enrolled participants will be blinded and evaluated blindly after the last treatment.

**Study type:**

Interventional study

**PICOs:**

P: There were 336 participants with primary dysmenorrhea who met the diagnosis and inclusion criteria, aged 16 to 35 years, with 4cm average pain visual simulation score (VisualAnalogueScale, VAS) for 3 consecutive menstrual cycles.

I: EA +SA

C: SA

O:The main purpose of this study is to evaluate the recent efficacy and safety of electroacupuncture for relieving primary dysmenorrhea after 12 weeks of treatment. The secondary purpose is to evaluate the long-term efficacy of electroacupuncture for treating primary dysmenorrhea through a 12-week follow-up observation, and to reveal the effect mechanism of electroacupuncture for treating primary dysmenorrhea using metabolomics technology.

S: This is a single-blind (assessors blind to group allocation), double-armed, randomised controlled trial on the feasibility and therapeutic efficacy of a two-week LLLA therapy for office workers with SNP.

**Intervention and exposure:**

Intervention group:

EA group

Hwato brand disposable acupuncture needles (size 0.30 ×40 mm), and SDZ-V （Suzhou Medical Device Co., Ltd）electroacupuncture apparatuses will be used. Participants in the EA group will be treated at the following acupoints: bilateral sanyinjiao (SP6), located in the medial calf, 3 cun (approximately 60 mm) above the tip of the medial malleolus (Fig 2A) behind the medial edge of the tibia; and guanyuan (CV4), located in the lower abdomen on the anterior midline, 3 cun below the umbilicus (Fig 2B). The acupoints will be routinely disinfected prior to treatment. Acupuncture needles will be inserted 0.8–1.5 cun (approximately 16–30 mm) into the skin. Needle insertion will follow an angle of 30–45° in an inferomedial direction for SP6.

Fig2. The point positioning diagram

The acupuncture needle will be inserted vertically into CV4. Following needle insertion, small, equal manipulations of twirling, lifting, and thrusting will be performed on all needles to reach de qi (a composite of sensations including soreness, numbness, distention, heaviness, and other sensations believed to be an essential component for acupuncture efficacy). After de qi is achieved, paired electrodes on the electric needle device will be connected to the needle handle on one side of SP6 and CV4. Electroacupuncture stimulation will last for 30 min a priori, with a continuous wave of 100 Hz, and current intensities of 1–5 mA (the skin around the acupoints shakes slightly, which participants can typically tolerate without pain). We will administer EA seven days before each menstrual cycle until menstruation is over. Each menstrual cycle equals one course of treatment. We will evaluate a total of three courses of treatment (i.e., three menstrual cycles) in the current study. A total of 21 treatments will be performed. During treatment, operators (i.e., operating personnel) will be separated from follow-up personnel. We will also control for other influencing factors to avoid information bias.

SA group

Participants in the sham acupuncture group will receive sham electric acupuncture with false (sham) acupuncture points. The sham SP6 point is located 2 cun (approximately 40 mm) above taixi (KD3, midway between the spleen and kidney meridians [Fig 2A]). The sham CV4 point is located between the qihai (CV6) and yinjiao (CV7) levels, 3 cun lateral to the midline (Fig 2B). SA acupuncture needles will be inserted into the surface of skin (2–3 mm). There was no acupuncture technique whatever and have no de qi. The same electrode placements and other treatment settings will be used as in the EA group. However, the internal power cord will be cut off without any actual current output. Throughout the trial, participants will be treated individually with procedures in place to prevent communication between participants. To test for the blinding effect of the participants, all participants will be asked to guess whether they received an EA or a SA within 5 minutes of one session at week 12.

**How the planned methods achieve the aim or research questions**

**Primary outcomes:**

Participants will have been diagnosed with PDMM according to the criteria specified in the International Classification of Diseases criteria (ICD-10-I63; Code: 902) [10]. Moreover, VAS scores will be recorded before and after treatment (i.e., during the follow-up period). We adopted a methodology based on the VAS card created by the Pain Society of the Chinese Medical Association, ranging from “no pain” (at 0 cm on the left side of the scale card) to “the most severe pain” (at 10 cm on the right side of the scale card). The distance from the starting point of the scale to the patient evaluation measurement is recorded in cm. The VAS score is measured by the patient placing the cursor at the area best representing their subjective level of pain at the time of evaluation. The score is recorded according to the position of the cursor

**Secondary outcomes:**

Pain level on this scale is indicated by 11 numbers ranging from 0 to 10 (0 = painless, 10 = the most painful). The degree classification standard was scored as follows: 0 = painless, 1 to 3 = mild pain, 4 to 6 = moderate pain, 7 to 10 = severe pain. The participants will be scored according to their individual pain sensations. This method is easily understood by patients and can be either dictated or written. The CMSS is a total frequency score indicating an average severity evaluation of menstrual dysmenorrhea and includes information on specific symptoms of menstrual dysmenorrhea. Moreover, the traditional Chinese medicine symptom Score is formulated to score the evaluation of Chinese medicine symptoms according to the Guiding Principles for Clinical Research published by the Chinese Medical Science and Technology Press. The Self-Rating Anxiety Scale, Self-Rating Depression Scale, and 36-Item Short Form Survey are scales used to evaluate participants' mood and quality of life during treatment and follow-up. A metabolomics test on blood and urine samples is to be used to evaluate metabolic product changes occurring after the treatment of PDMM with EA and to provide findings informing the evidence base about putative mechanisms associated with metabolic pathways affected by EA.

**Statistical analysis:**

Descriptive statistics

We will perform statistical analyses using SPSS statistical software (version 26.0, IBM Corp., Armonk, NY, USA). A mixed-effect model will be used to compare change values before and after treatment for continuous variables. This analysis model divides the baseline scores as study covariates, accounting for grouping and central effects. Based on this model and differences before and after treatment, we will calculate the least squares mean of the two groups and the associated 95% credible interval of the difference between the two groups. Fisher's exact parametric method and nonparametric Kruskal Wallis tests will be used to evaluate dichotomous variables. Report exact p-values for all values greater than or equal to 0.001.A two-sided P-value of < 0.05 is considered the threshold for statistical significance.

Metabolomics analysis

We will perform first signal denoising, baseline and phase correction for all spec graphs, piecewise data integration, row and column normalization, and scaling normalization to conduct the metabolomics analysis. All data are to be processed using MestReNova software (Mestrelab Research, Santiago de Compostela, Spain). Pattern recognition analysis includes adopting the principal component analysis method of unsupervised mode recognition as well as a classification analysis (SIMCA), a partial least squares method, and a neural network method. All of these procedures will be conducted using SIMCA-P software (version 14. 0, Umetrics AB, Umea, Sweden). Pearson correlation analysis will be used to analyze characteristic hematuria metabolites as well as to determine the metabolites specific to PDMM and their associated metabolic pathways.

**Recruitment**

The study will be conducted by the University of South China (the central research headquarters). However, there are three centers participating in this project: the First Affiliated Hospital, Hengyang Medical School, University of South China; the First Affiliated Hospital of Hunan University of Traditional Chinese Medicine; and the Affiliated Changsha Central Hospital, Hengyang Medical School, University of South China. Participants will be publicly recruited according to three major strategies. The first strategy is to recruit participants from the three aforementioned hospitals. The second strategy is to print recruitment posters and distribute them across major health institutions and frequented public places to recruit potentially eligible research participants. The third strategy is to publish online advertisements introducing our study and recruiting volunteer participants.

**Inclusion criteria**

Eligible participants must meet all of the criteria below to be considered for this study:

1. Diagnosed with PDMM according to the International Classification of Diseases criteria (ICD-10-I63; Code: 902)

(2) Aged 16–35 years.

(3) Regular menstrual cycles (defined as 28-day cycles ± 7 days) for 4–7 menstrual days.

(4) Detailed contact information provided, with no short-term migration and ability to attend follow-up visits.

(5) Received no treatments within the preceding month, including acupuncture, NSAIDs, anti-inflammatory drugs, and analgesics.

(6) Willing to cooperate with the treatment procedures, medical examination, and efficacy evaluations, and must agree to not participate in other clinical experiments during the study period.

(7) Provision of written informed consent prior to participation.

(8) VAS score (i.e., the primary study endpoint described above) for three consecutive menstrual cycles ≥ 4 cm.

**Exclusion criteria**

Participants were excluded for any one of the following:

(1) Diagnosis of secondary dysmenorrhea (i.e., dysmenorrhea caused by uterine fibroids, adenomyosis, endometriosis, pelvic inflammation, internal foreign bodies, ovarian lesions, or other organic lesions).

(2) Irregular menstrual periods

(3) History of thrombosis, embolism, cerebrovascular disease, coronary artery disease, or high risk for thrombosis.

(4) Mental illness or cognitive impairment preventing comprehension of scale evaluation content.

(5) History of complications, depression, or antidepressant medication use.

(6) Severe heart, lung, liver, kidney, blood, immune, or endocrine system diseases.

(7) Hematopoietic diseases, acquired immunodeficiency syndrome, tuberculosis, hepatitis, or miscellaneous infectious diseases (as differentiated from the specific infections defined above).

(8) Malignancy, history of malignancy, or malignancy findings.

(9) Pregnancy, planned pregnancy, or lactation within 1 year.

(10) History of jaundice or herpes during pregnancy.

(11) Participation in other clinical studies within 4 weeks before provision of informed consent.

(12) Use of any of the following drugs within 4 weeks before provision of informed consent: gonadotropin-releasing hormone analogues or testosterone derivatives, hormone preparations containing mainly progesterone or estrogen, estrogen antagonists or aromatase inhibitors, and ongoing treatment for other gynecological diseases.

(13) Metal allergy or severe needle phobia such that electroacupuncture treatment cannot be tolerated.

(14)Skin rupture from needle, scars, or cardiac pacemaker.

(15) Other lesions or conditions deemed inappropriate or too complicated according to the judgment of the researchers, such as frequently changing work environment or unstable living conditions, which easily cause loss to follow-up.

**How will participants be identified and initially contacted including screening processes?**

The snowballing process and public advertisement can identify potential participants. People interested in participating in the study will be encouraged to contact the research team via email for an eligibility check. We will send online screening checklist, including Various scales,to assess their suitability for participation.

**How will formal recruitment of potential participants be conducted?**

This study will use a PROC PLAN software environment (SAS version 9.4, SAS Institute Inc., Cary, NC, USA) for stochastic randomization. A third party produced a confidential dynamic randomization scheme using the Interactive Web Randomization System (Beijing LNKMED Tech Co., Ltd., Beijing, China, http://www.lnkmed.com/edc/a/sysIndex). The group assigned to each participant will be determined by a randomized file. Files will be randomly generated according to statistical units (SAS blocks). When the appropriate district length is selected, a random number of 336 participants will be generated for each of the three centers at a 1:1 ratio (this number shall not be reused) and will include the treatment assignments with corresponding serial numbers: 1001-1336, 2001-23316-336, and 3001-3336 (i. e., random coding table). After screening each eligible participants., the researchers at each center who did not participate in the treatment or evaluation were asked by the research assistant (non-blind personnel) to log into the randomized system and issue the center number randomly according to the enrollment order of the participants . For participants who complete the composition work although do not receive treatment, the random number cannot be reassigned to another participant. The next enrolled participant will receive the next random number in order. To ensure that each participant will receive the same chance of trial conditions in this randomized controlled study, we will balance the influence of confounding factors, avoid trial bias caused by subjective and random arrangements, and provide a scientific basis for statistical analysis. Participants from the three centers who are eligible for enrollment will be randomly assigned to receive an electric or sham needle using a 1:1 ratio. The present study design has been demonstrated as the most effective method for avoiding selection bias. Specifically, after allocation within the LNKMED Interactive Web Randomization System by a research assistant who will not be participating in the treatment and evaluation procedures, basic patient information will be entered into the allocation system to enroll patients meeting the inclusion criteria into the randomization process and obtain the corresponding randomization numbers and groupings. After enrollment, the clinical researcher will record the demographic information, including age, race, marital status, education level, employment status, comorbidities, and body mass index, within the study data system. Moreover, the duration of dysmenorrhea, treatment history, and related scale scores will be recorded before treatment. The study protocol includes regular monitoring and bias evaluations among centers.

The randomization scheme and parameters set during scheme generation are collectively known as the blind background. This study follows a first-level blind design, in which the primary randomized number corresponds to the actual treatment group. The random coding table is generated by the statistical unit, and the blind bottom will be sealed separately and delivered to the responsible clinical trial unit at The First Affiliated Hospital, Department of Chinese Medicine, Hengyang Medical School, University of South China.

The central randomization system designates strict personnel authority. More specifically, no one other than the highest administrator is allowed to view the random scheme within the system, thereby ensuring a completely random distribution and effectively eliminating human interference and error.

The investigator will not perform emergency unblinding unless grouping information is required to medically treat the patient.

In this trial, the emergency unblinding process involves electronic emergency letters. When the investigator believes that the grouping information is required for the treatment of serious adverse events, emergency unblinding can be achieved through the LNKMED Interactive Web Randomization System. Meanwhile, the researcher will record the relevant data in detail, including the time of unblinding, cause, trial treatment, treatment, etc. Once the electronic emergency envelope is opened, the participant will be treated as a shedding case.

**Please provide a response to any ethical issues and concerns raised by the recruitment process such as the relationship between researchers and participants, risks associated with recruitment strategy, and the nature of the population being examined.**

There is no single correct approach, but a balanced recruitment approach will be employed (e.g., use snowballing as well as public advertisement). Recruitment of participants should be viewed as part of the research protocol and should require appropriate informed consent of the already-enrolled participant. Investigators should inform prospective participants why they are being contacted, how information about them was obtained, and what will happen to that information if they decide not to participate.

**Data collection method and location including who will be responsible for the data collection**

Participation in this research project is voluntary. If one does not wish to take part, they do not have to. If participant decided to take part and later changed their mind, they are free to withdraw from the project at any time. If they decide to withdraw from the project after the data has been analysed, we will not be able to remove the individual data as this cannot be identified.

If participant do decide to take part, they will be given the participant information letter and consent form to sign, and they will be given a copy of the information letter to keep. They decision to take part, or to take part and later withdraw, will not affect they relationship with the research team and any staff within the School of Nursing and Midwifery at ECU.

By signing the consent form, they consent to the research team collecting and using personal information about the participant for the research project. Any information obtained in connection with this research project that can identify participant will remain confidential. When all survey responses are returned to the research team, all data are automatically de-identified, and they will not be identifiable by any of their responses to the survey. They information will only be used for the purpose of this research project and it will only be disclosed with they permission, except as required by law.

It is anticipated that the results of this research project will be published and/or presented in a variety of professional forums. In any publication and/or presentation, the information will be provided in such a way that participants cannot be identified, except where requested for specific reasons, and then they will be asked to provide written consent.

In accordance with relevant laws, participants have the right to request access to the information about them that is collected and stored by the research team. They also have the right to request that any information that they disagree to be corrected. They are encouraged to inform the research team member named at the end of this letter if they would like to access they information.

All data collected will be kept in accordance withData Management Policy. Electronic data will be stored on a secure Microsoft SharePoint site provisioned byBeijing LNKMED Tech Co., Ltd., Beijing, China, http://www.lnkmed.com/edc/a/sysIndex Services and physical records will be stored as required in Records Management Policy. The data will be retained for a period of seven years and destroyed, if appropriate at the end of the retention period. Data will be de-identified when stored and at the end of the retention period, the data will be destroyed, if appropriate under the State Records Act.

There are no foreseeable risks associated with participation in this research project.

We will advise participants of the outcomes via email communication. We also intend to publish our results in research journals and present them at research conferences locally, nationally and internationally. Participants' name or any other identifying information will not be included in any of the publications or presentations.

The Principal Investigator (PI) will log in to the EDC system, check the consistency of case report form and source data, and record the case report within the specified time. The researcher can answer the questions online or download the list of questions and answer offline. Then, the researcher input the questions into the EDC. The data administrator and supervisor can approve the researcher to answer questions and ask questions again if necessary.

The proposed randomized controlled trial was approved by the China Ethics Committee of Registering Clinical Trials (approval no: ChiCTR2100054234; http://www.chictr.org.cn/; version 2.0, as of December 9, 2021). This study is preparing for the patient recruitment stage.

**Statistical analysis**

For the primary outcome, rates of recruitment (numbers consented/eligible), completion (undertaken baseline and follow-up tests), adherence (participant completed sessions/number of sessions), and adverse events (number and number per participant hour) will be calculated. Secondary outcome will be assessed following intention-to-treat principles. Repeated measures ANOVA will be conducted to assess changes in secondary outcomes throughout the study. This model allows inclusion of missing data in an intention-to-treat analysis without imputations (e.g. last-observation-carried-forward). If necessary, analysis will be adjusted for baseline levels and potential confounding factors. Normality assumptions will be assessed using the Shapiro-Wilk test. Statistical significance will be set at an alpha level of 0.05. Corrections will be applied to all analysed outcomes to account for multiple comparisons.

**Risk**

**Physical Risks**

All adverse events shall be recorded in detail and handled and tracked until properly resolved or stable, and serious adverse events shall be reported to the ethics committee and competent authorities as required according to institutional regulations and relevant laws. The principal investigator shall conduct a cumulative review of all adverse events and hold an investigator meeting if necessary to reassess the risks and benefits of the study in light of the new information arising during the course of the study. Once an adverse event is identified, this event shall be followed-up until it is resolved or until the adverse event is determined to be permanent.

Moreover, researchers must assess changes in the severity of the adverse events occurring during the course of the study, suspicious reactions to the study treatment, and the need for intervention at each visit (including recommending increased study visit frequency if needed). All adverse events must be recorded on the adverse events page of the CRF. Adverse events must be recorded with regard to time, severity, and duration parameters, as well as with relevant information on measures and outcomes. Adverse events must be carefully and accurately recorded during the trial. Adverse events are to be divided into acupuncture-related and non-acupuncture-related events according to their potential reactions with the acupuncture treatment process.

**Participant Information Letter**

**Project title:** Electroacupuncture treatment of primary dysmenorrhea: a randomized, participant-blinded, sham-controlled clinical trial

**Approval Number:** 20214310NHYCG04

**Trial registration No.** ChiCTR2100054234

**Principal Investigator:** Dr Xiao Xue

**An invitation to participate in research**

You are invited to participate in a project titled “Electroacupuncture treatment of primary dysmenorrhea: a randomized, participant-blinded, sham-controlled clinical trial ”.Fourteen days before the study and 1 day before treatment you need to do urine pregnancy examination (free), after participating in the study, you can get free professional examination to clear diagnosis, you can get 21 days of specialist clinic free acupuncture treatment, acupuncture arranged by special outpatient clinic can ensure your privacy information security at the same time without waiting in line. In addition, we will follow you for 3 months after treatment to assess if your symptoms are relieved.

If the participants can participate in the study, the doctor will be randomly assigned to either the electroacupuncture group or the modern electroacupuncture group of 168 participants each. Patients in the electroacupuncture group received three menstrual cycles, 7 days before menstruation every week, with 21 injections; the follow-up period was 24 weeks with no treatment. Patients in the modern electroacupuncture group received 3 menstrual cycles, treated 7 days before menstruation every week for 7 times, totaling 21 injections; the follow-up period was 24 weeks with no treatment. Traditional electroacupuncture treatment, relatively deep acupuncture point puncture; modern electroacupuncture treatment, relatively shallow puncture; due to the body adaptability, the electrical stimulation may not be felt in the treatment process. participants need to come to the hospital on time for treatment and examination, for outpatient follow-up. During the study period, patients must complete blood and urine tests, transabdominal B ultrasound test, and complete the relevant scale test. Also, the participant had the responsibility to report to the physician on any physical and mental changes during the trial, regardless of whether the participant believed that this change was relevant to this study. In principle, no other treatment and medication should be received and require contraception during the study.

In this study, your blood samples and urine samples are needed to improve the examination indicators. The hematuria samples are stored until the end of the experiment, this process requires you to sacrifice time to cooperate with our examination. In addition, in the process of the study, you need to go to the hospital seven times a month for three consecutive months for acupuncture treatment. You need to make arrangements in advance within these 3 months and complete all the treatment as far as 3 months.Please read this information carefully. Ask questions about anything that you do not understand or want to know more about. Before deciding whether to take part, you might want to talk about it with a relative or friend.

If you decide you want to take part in the research project, you will be asked to sign a consent form. By signing it, you are telling us that you:

Understand what you have read;

Consent to take part in the research project;

Consent to be involved in the research described;

Consent to the use of your personal information as described.

**What is this project about?**

Primary dysmenorrhea (PDMM) is diagnosed when spastic pain occurs during the menstrual cycle and organic genital lesions have been excluded in a differential diagnosis. In 2020, an article published in the Journal of the American Medical Association reported that PDMM affects 50–90% of women worldwide, causing substantial impacts on work and quality of life. Moreover, a recent study reported that hypertension during pregnancy is closely related to dysmenorrhea in early adulthood. Therefore, the primary prevention of dysmenorrhea, as well as slowing the disease progression and developing effective and low risk treatment modalities, are priorities within modern medical research.

At present, the Western medicine approach to treating dysmenorrhea mainly includes non-steroidal drugs. More specifically, drugs commonly prescribed for this condition include ibuprofen and oral contraceptives. However, the failure rate of these drugs is as high as 25% due to contraindications or intolerance. Moreover, these medications are associated with a high risk of adverse side effects. The long-term use of such drugs can cause liver, kidney, and digestive system disorders; inhibit ovulation; thin the endometrium; cause a series of adverse reproductive reactions (including impacts on menstrual volume), headache, and drowsiness; and even increase breast cancer risk .

In contrast, according to a recent meta-analysis, the effect of electroacupuncture (EA) on controlling PDMM symptomology was statistically significant in both animal experiments and clinical epidemiologic investigations. Moreover, EA serves as a safe and effective “green” therapy and therefore may be an excellent candidate as an alternative therapy within Western medicine.

Currently, whether EA can relieve pain within PDMM is controversial worldwide. Thus, this multicenter study aimed to develop a validated protocol for treating PDMM symptomology by applying EA at specific acupoints using a sham acupuncture (SA) control group. The proposed study protocol describes a randomized, controlled, participant-blinded experiment. Specifically, the proposed study evaluates the efficacy of EA in alleviating PDMM as well as the use of metabolomic techniques to reveal the associated effector mechanisms, discover potential biomarkers, and derive associated metabolic pathways. Through this research, we aim to explain the mechanisms mediating the effects of EA on PDMM symptomology at the overall biological level.

The primary aim of our study is to add to the existing evidence base on the effects of EA treatment on PDMM symptomology. Furthermore, we aim to find a suitable alternative to pharmaceutical treatment in patients with PDMM to reduce reliance on non-steroidal anti-inflammatory drugs (NSAIDs).

**Who are the people should not participate in this project?**

Participants were excluded for any one of the following:

(1) Diagnosis of secondary dysmenorrhea (i.e., dysmenorrhea caused by uterine fibroids, adenomyosis, endometriosis, pelvic inflammation, internal foreign bodies, ovarian lesions, or other organic lesions).

(2) Irregular menstrual periods

(3) History of thrombosis, embolism, cerebrovascular disease, coronary artery disease, or high risk for thrombosis.

(4) Mental illness or cognitive impairment preventing comprehension of scale evaluation content.

(5) History of complications, depression, or antidepressant medication use.

(6) Severe heart, lung, liver, kidney, blood, immune, or endocrine system diseases.

(7) Hematopoietic diseases, acquired immunodeficiency syndrome, tuberculosis, hepatitis, or miscellaneous infectious diseases (as differentiated from the specific infections defined above).

(8) Malignancy, history of malignancy, or malignancy findings.

(9) Pregnancy, planned pregnancy, or lactation within 1 year.

(10) History of jaundice or herpes during pregnancy.

(11) Participation in other clinical studies within 4 weeks before provision of informed consent.

(12) Use of any of the following drugs within 4 weeks before provision of informed consent: gonadotropin-releasing hormone analogues or testosterone derivatives, hormone preparations containing mainly progesterone or estrogen, estrogen antagonists or aromatase inhibitors, and ongoing treatment for other gynecological diseases.

(13) Metal allergy or severe needle phobia such that electroacupuncture treatment cannot be tolerated.

(14)Skin rupture from needle, scars, or cardiac pacemaker.

(15) Other lesions or conditions deemed inappropriate or too complicated according to the judgment of the researchers, such as frequently changing work environment or unstable living conditions, which easily cause loss to follow-up.

**What does my participation involve?**

If the participants can participate in the study, the doctor will be randomly assigned to either the electroacupuncture group or the modern electroacupuncture group of 168 participants each. Patients in the electroacupuncture group received 3 menstrual cycles, 7 days before menstruation every week, with 21 injections; the follow-up period was 24 weeks with no treatment. Patients in the modern electroacupuncture group received 3 menstrual cycles, treated 7 days before menstruation every week for 7 times of 21 injections; the follow-up period was 24 weeks with no treatment. Traditional electroacupuncture treatment, relatively deep acupuncture point puncture; modern electroacupuncture treatment, relatively shallow puncture; due to the body adaptability, the electrical stimulation may not be felt in the treatment process. participants need to come to the hospital on time for treatment and examination for outpatient follow-up. During the study period, patients must complete blood and urine tests, transabdominal B ultrasound test, and complete the relevant scale test. Also, the participant had the responsibility to report to the physician on any physical and mental changes during the trial, regardless of whether the participant considered the change to be relevant to this study. In principle, no other treatment and medication should be received and require contraception during the study.

**Do I have to take part in this research project?**

Your participation in this research project is voluntary. If you do not wish to take part, you do not have to. If you decide to take part and later change your mind, you are free to withdraw from the project at any time. If you decide to withdraw from the project after the data has been analysed, we will not be able to remove your individual data as this cannot be identified.

If you do decide to participate, you will get a consent form to sign, and you will receive a duplicate copy of this information letter saved. Your decision to attend or participate and later withdraw will not affect your relationship with any staff of the research team.

**Your privacy**

Participating in this study, the participant's personal privacy will be kept strictly confidential as permitted by law, and we will protect your information from unauthorized contact to others, including removing those that can easily identify you. Only for the researchers of the research team. Inspectors, inspectors, ethics committees, and administrators should be allowed to directly access the participant's original medical records in order to verify the procedures and data of clinical trials. Your personally identifiable information does not appear in the publication when the results are published. Data security measures are uPDMated when new technologies emerge. If the data may be transferred to a third party for research and use, we will review the qualifications of the third party to ensure the safety of the data and sample storage. To ensure that the study is conducted in accordance with the regulations, members of the government administration or the ethics committee can have access to your personal data at the research unit, if necessary.

**Possible Benefits**

Based on past studies, we anticipate that the electroacupuncture treatment that we will provide may help to alleviate the level of primary dysmenorrhea and improve work efficiency and quality of life. We hope that our results can be used to let us understand how to better help us understand the pathogenesis of the disease, promote the improvement of medical care, promote the development of safer or more effective diagnosis and treatment methods, and expand new scientific knowledge.

There are no foreseeable risks associated with your participation in this research project.

**Possible Risks and Risk Management Plan**

There are no known risks to participating in this research project.

**What happens when this research study stops?**

We will advise you of the outcomes via email communication. We also intend to publish our results in research journals and present them at research conferences locally, nationally and internationally. Your name or any other identifying information will not be included in any of the publications or presentations.

**Has this research been approved?**

The proposed randomized controlled trial was approved by the China Ethics Committee of Registering Clinical Trials (approval no: ChiCTR2100054234; http://www.chictr.org.cn/; version 2.0, as of December 9, 2021). This study is preparing for the patient recruitment stage.

**Contacts**

If you would like to discuss any aspect of this project, please contact the following people.

| **Chief Investigator** | |
| --- | --- |
| Dr XiaoXue | |
| 1.College of Acupuncture, Massage and Rehabilitation, Hunan University of Chinese Medicine, Changsha, China  2.The First Affiliated Hospital, Department of Chinese Medicine, Hengyang Medical School, University of South China, Heng Yang, Hunan, China | |
| Edith Cowan University | |
| P: 0734-8279333 | 15211879577 |
| E:xuexiao19860417@163.com |  |

If you have any concerns or complaints about the research project and wish to talk to an independent person, you may contact:

| **Independent Person** |
| --- |
| Research Ethics Support Officer |
| Hengyang Medical School, University of South China |
| P: 0734-8578934 |
| E: nhfyllwyh@163.com |

Participant Consent Form

Informed notice section

Dear lady:

Primary dysmenorrhea (primary dysmen orrhea, PDM) is a common gynecological disease. In 2020, JAMA recently reported that primary dysmenorrhea affects 50% to 90% of women around the world, and seriously affects the work and quality of life ofpatients. Therefore, the prevention of dysmenorrhea, slowing down the progress of the disease course and treatment has become the focus of modern medical research.Currently commonly used treatments include oral medication and physical therapy.Oral drugs such as ibuprofen have been used as a routine treatment for primary dysmenorrhea; physical therapy such as microwave and hot compress is often used as an adjuvant therapy to improve menstrual pain.You can choose your preferred treatment to include oral medication, physiotherapy, etc, without being forced into the study.

The existing research results confirm that electroacupuncture therapy has good recent efficacy and certain continuous effect on improving the clinical symptoms of PDM patients, and is safe and low cost, and has great advantages and prospects in clinical application, but strict and clinical trials need to be designed to confirm it.Multi-center randomized controlled Clinical Study of Primary dysmenorrhea is the 4310 Medical Clinical Research Program ofthe University ofSouth China. It isjointly undertaken by the First Affiliated Hospital ofthe University of South China, the First Affiliated Hospital of Hunan University of Traditional Chinese Medicine, and Changsha Central Hospital Affiliated to the University of South China.The three hospitals are all tertiary class A general hospitals integrating medical treatment, scientific research and teaching. The purpose of the project is to evaluate the effectiveness and safety of electroacupuncture in improving the clinical symptoms and quality of life ofpatients with PDM, and to explore the mechanism of electroacupuncture in the treatment ofblood and urinary metabolomics ofprimary dysmenorrhea.You will not benefit directly from attending this study.Using your biobank and information for research may help us understand the pathogenesis of the disease, promote improved medical standards, promote the development of safer or more effective diagnosis and treatment methods, and expand new scientific knowledge.If you are interested to understand this study, you can get free professional consultation and scale assessment ofthe severity ofPDM symptoms.Fourteen days before the study and 1 day before treatment you need to do urine pregnancy examination (free), after participating in the study, you can get free professional examination to clear diagnosis, you can get 21 days of free specialist outpatient electrical acupuncture treatment, arranged by the special acupuncture clinic can ensure your privacy information security at the same time without waiting in line.In addition, we will follow you for 3 months after treatment to assess if your symptoms resolve.Upon inspection, Western medicine diagnosis system by uterine fibroids, adenomyosis, endometriosis, pelvic inflammation, internal foreign body, Secondary menstrual cramps caused by ovarian lesions; Menstrual period is successively irregular; Patients with serious cardiovascular and cerebrovascular diseases, serious diabetes, serious infection, liver and kidney insufficiency, hematopoietic diseases, HIV, and tuberculosis, hepatitis and other infectious diseases; patients with serious cardiovascular and cerebrovascular diseases, serious diabetes, serious infection, liver and kidney insufficiency, hematopoietic diseases, HIV, and tuberculosis and hepatitis; Pregnant or planned pregnancy, lactation and December postpartum within 1 year; Those who are participating in other clinical trials should not participate in this study. If the participants are able to participate in this study, the doctors will be randomly assigned to the electric needle group or the modern electric needle group of 168 people each. Patients in the electroneedle group received 3 menstrual cycle electroinjections, 7 days before menstruation,7 times for 21 injections; the follow-up period was

24 weeks with no treatment.Patients in the modern electroneedle group received 3 menstrual cycles ofmodern electricity Needle treatment, 7 days before menstruation, 7 times, 21 injections; the follow-up period was 24 weeks with no treatment.Traditional electroacupuncture treatment causes deep acupuncture points; due to the body adaptability, electrical stimulation may not be felt during the treatment.participants need to go to the hospital on time for treatment and examination for outpatient follow- up.During the study period, patients must complete blood and urine tests, pass abdominal B-ultrasound examination, and complete the relevant scale test.Also, the participant was responsible to report to the physician any physical and mental changes in the course of the trial, regardless ofwhether the participant considered the change to be relevant to this study.In principle, other treatment and medication should not be received and the need requires contraception.We promise that in case of acupuncture-related adverse events such as acupuncture, pain, subcutaneous hematoma, we will immediately stop the acupuncture operation, suspend the trial and give the sleeper, pain, swelling and other treatment; the study-related damage, give the most routine follow-up treatment according to professional judgment, the cost will be borne by the hospital.The whole experiment will be given a full free consultation, scale measurement (including the follow-up period), free acupuncture treatment, and the necessary specialist and laboratoryexaminations.

Expected conditions and reasons for possible termination in this trial include needle sickness, intolerable needle pain, local subcutaneous hematoma in the acupuncture, sudden acute exacerbation of PDM symptoms and long- term oral PDM-related symptoms improvement medication during treatment.In general, the symptoms of acupuncture, needle pain and subcutaneous hematoma are mild. If the participant has the acute symptoms of PDM during the trial, we will evaluate them with specialists and deal with them in time.In this study, your blood samples and urine samples are collected in order to fully improve the examination indicators. The preservation of hematuria samples occurs until the end ofthe experiment, and this process requires you to sacrifice your time to cooperate with our examination.In addition, in the process of research, you need to go to the hospital for acupuncture for three consecutive months a month. You need to make arrangements in advance within these 3 months and complete all the treatment for 3 months as far as possible.

Participating in this study, participants ' personal privacy will be kept strictly confidential as permitted by law, and we will protect your information from unauthorized other contacts, including removing those that can easily identify you.Check only for the researchers ofthis research team.Supervisors, inspectors, ethics committees, and administrative authorities shall be allowed to directly access the original medical records for the verification of procedures and data for clinical trials without violating the permission of applicable laws and regulations.Your personally identifiable information does not appear in the publication when the results are published.Data security protection measures are updated promptly when a new technology appears.Ifthe data may be transferred to a third party for research and use, we will review the qualifications ofthe third party to ensure the safety ofthe data and sample storage.To ensure that the study is conducted as required, members of the government administration or theethics committee may access your personal information at the research unit as required.participants had the right to decide to withdraw from the study at any time without any discrimination and retaliation,and will not affect any medical services.If you make a consent decision, you can later withdraw your consent,which does not affect the effectiveness of processing activities based on biological samples and data conducted based on personal consent.Ifyou change your decision, please contact:Xiao Xue , Tel.: 15211879577. participants have the right to ask our doctor at any time and, ifcomplaining, contact the Ethics Committee Office at

0734-8578934,Email: [nhfyllwyh@163.com](mailto:nhfyllwyh@163.com)

If information is obtained that may affect the trial, the participant or his legal agent will be promptly notified.

Basic research information of researcher and qualification of research institution:

The trial was led by Professor Liu Xin, director of the Traditional Chinese Medicine Department of the First Affiliated Hospital of the University of South China and a famous Traditional Chinese medicine of Hunan Province. The Department of Traditional Chinese Medicine of the First Affiliated Hospital of the University of South China has a high reputation in the field of acupuncture and moxibustion. The department currently undertakes a number of provincial and ministerial and national projects.Director Liu Xin has presided over national and provincial projects in the past five years.He has served as the director of the Department ofTraditional Chinese Medicine ofthe First Affiliated Hospital ofthe University ofSouth China for

many years.

Agree to the signature section

I have been informed ofthe background, purpose, steps, risks and benefits ofthe study.I have enough time and opportunity to ask questions and I am satisfied with the answer to the question.I have also been told who

should be contacted when I have problems, want to reflect difficulties, concerns, have advice on research, or want to get further information, or provide help with research.I have read this informed consent form and fully understand the consent to participate in this study.I know that I can withdraw from this study at any time without any reason. I am willing to accept the

research requirements and work with the researcher. I voluntarily and actively cooperate with the relevant inspection to fulfill the rights and obligations of the participants to ensure the final completion ofthis study.

Participant

(signature):

Signed on by: year month say

Contact telephone number:

The investigator's statement:

I confirm that the details of this study have been explained to the patients, particularly to the possible risks and benefits arising from participation in this study .

Investigator signature:

Date:

Investigator Contact Information ：
