# Supplementary material for: Electroacupuncture treatment of primary dysmenorrhea: A randomized, participant-blinded, sham-controlled clinical trial protocol
Source: PLoS One. 2023 May 26;18(5):e0282541. doi: 10.1371/journal.pone.0282541 (PMC10218736; doi:10.1371/journal.pone.0282541)
Supplement: S1 File — (PDF) [file pone.0282541.s004.pdf]

Electroacupuncture Treatment of Primary Dysmenorrhea: A multicenter randomized controlled clinical study

Case report form

Research sponsor: The First Affiliated Hospital of South China University

Start time:____Year___Month _Day

End Time： ___Year___Month _Day

Research center:

☐01 The First Affiliated Hospital, Department of Chinese Medicine, Hengyang Medical School,University of South China, Heng Yang, Hunan, China

□02 College of Acupuncture, Massage and Rehabilitation, Hunan University of Chinese Medicine, Changsha , China

□03 The Affiliated Changsha Central Hospital, Hengyang Medical School, University of South China.

Version number: 2.0

Participants randomized number：|_|_|_|

Participants name abbreviation：|_|_|_|_|

Contact address：______________________

Telephone number：___________________

The researcher signed：________________

Instructions for filling out the case observation form

1..This case report form must be filled out by a full-time evaluator and completed by the same person whenever possible.

2.Fill form in pen or signature pen, not in pencil or ballpoint pen.

3.Those who pass the screening should formally fill in the case report form, and those who suspend treatment should truthfully record the time and reasons for withdrawal from the study.

4.The case must be filled in accurately and clearly, and must not be corrected at will. The error should be marked in the horizontal middle, and the name abbreviation of the modifier and the modification time should be signed.or example：~~57~~75$^{\text{ws 2018/10/1}}$。

5.The entry sequence number shall be filled in by each hospital according to the time order of Participant visit (order of entry sequence number， It should be consistent with the visit sequence of time and the order of visit number).

6.The first two letters ofthe two-character name should be filled in; the three-character name should be filled in with the first letter ofthe third character and the second letter ofthe third character; the four-character name should be filled in with the first letter ofeach character.Example: Zhang Hong **Z H H O**,Li Shuming **L S M I**,Ouyang Xiaohui **O Y X H.**

7.All selected items are marked with √ in □. as: ☑.

8.If the item is not checked or missed for some reason, fill in " ND " , the specific dose and time are unknown, fill in " UK ", "cannot provide" or "not applicable" fill in " NA " .

9.Date adopts the international ISO 8601 date format: yyyymmdd. For example: On October 15,2018, written as: 2018 1015. Please fill in "UK" for date unknown date. For example: 201010UK, 2010UKUK, UKUKUK. Informed consent, medication history, concomitant medication, and adverse events must be completed. Time is used for 24 hours, such as 21:00 instead of 09:00pm, midnight record 00:00 (not 24:00) and as a new day, such as 00:00 on September 2,2012, and not 24:00 on September 1,2012

10.Do not use cross-referenced words, such as: with before, with above, etc. When describing medical history and adverse events, please use medical terms, please use the full name, do not use abbreviation or abbreviation,Unless these abbreviations or abbreviation have been listed in the study protocol.

11.Fill in the adverse event record form truthfully during the test. The time, severity, duration, measures taken and outcome of adverse events were recorded. In case of serious adverse events (including hospitalization, extended treatment time, disability, working ability, life-threatening or death events that occur during the clinical trial), the research group of Traditional Chinese Medicine Department of the First Affiliated Hospital of the University must be notified to South China immediately. Contact: Xiao ; tel: 0734-8279333

12.Clinical trials should be conducted in strict accordance with the clinical trial protocol. Inspection and records required in different periods of the test should be performed according to the clinical research flow chart.

13.All screening cases were filled in with screening forms and the screening data were entered into the central randomized database within 1 week.The included patients entered the data into the electronic database within 1 month. Before the research group data review, each score is in For each case of data completed, you must first conduct your own data review, supplement and revise the missing and logical and problematic data immediately; each case should have a branch data review record, and submit the electronic version to the scientific research secretary of the general research group

14.Falling cases must fill in the efficacy evaluation of CRF form by telephone inquiry. Note that even if the patient does not have treatment or suspend treatment, they should find ways to fill in the whole CRF form and the cause of shedding as far as possible

Catalogue

1、Flow chart of the clinical trial

2、General data, medical history, treatment history, drug allergy history, and other existing diseases and medication, uterus and attachments, diagnosis of Traditional Chinese and Western medicine, etc

3、Inclusion criteria, and exclusion criteria

4、Each scale and evaluation: visual simulation rating scale (VAS), pain numerical rating scale (NRS), COX dysmenorrhea symptoms scale (CMSS), traditional Chinese medicine symptom rating scale, anxiety self-rating scale (SAS), depression self-rating scale (SDS), quality of life scale (SF-36), expectation value evaluation, safety and blindness evaluation, overall self-efficacy evaluation

5、laboratory examination

6、Summary of the test completion situation

7、Emergency medication

8、Concomitant treatment

9、Adverse events

10、Evaluation of compliance

11、The CRF Audit Statement

Flow chart of the clinical trial

Study period	Enrollment	Allocation	Treatment period			Follow-up period		
Time point (week)	-1	0	4	8	12	16	20	24
Register	X							
Sign the informed consent form		X						
Inclusion and exclusion criteria		X						
Randomization		X						
Registration of population data		X						
Previous medical history and treatment history		X						
Comorbidities and medication history		X						
Laboratory basic examination		X			X			
Intervation								
Electric acupuncture			—	—	—			
sham electric acupuncture			—	—	—			
Aassessments								
VAS		X			X			X
NRS		X			X			X
CMSS		X			X			X
Traditional Chinese medicine symptom score		X			X			X
SAS		X			X			X
SDS		X			X			X
SF-36		X			X			X
Blind method evaluation					X			
Security observation			X	X	X			
Record adverse events			X	X	X			
Quality Control Assessment			X	X	X			
Evaluation of compliance			X	X	X			X

Approval Number	Random number □□	Treatment number □□	Patient name phonetic alphabet □□□□		Page：<br>Screening period

一般资料

normal information

Name □□□□ Gender: Female

Nationality Married or not □ Occupation □□

Nationality:________________Zip code □□□□□□

Case source: outpatient clinic □ sickroom □

Day of birth : |_|_|/|_|_|/|_|_|_|_|
month day year

height□□□ cm weight□□□ kg

blood pressure□□□ / □□□mmHg heart rate □□□ times / min

Medical history、 treatment history、 drug allergy history

Primary dysmenorrhea diagnosis time ____year____month___day

Treatment history: Western medicine treatment □
Chinese treatment □
Chinese and Western Medicine □

Therapeutic drugs: Drug name______________

Dose___________________

Family History: Have□ None□ Relatives□□

History of drug allergies:________________________________

Observing physician's signature:_________________ Date:___year___month___day

					Page:
Approval Number	Random number □□	Treatment number □□	Patient name phonetic alphabet □□□□		Screening period

History of other diseases and treatment

There was any history of other diseases and treatment □no □yes							
Disease name fit	Drug / treatment Spa name	Master single test dosage	rate *	Treatment start time （year/mouth/ day/）	Whether still cure cure	Treatment end time （（year/mouth/day/）	Is the disease recure
				|_|_|_|_|\\\|_|_|\\\|_|_|	□Yes □No	|_|_|_|_|\\\|_|_|\\\|_|_|	□Yes □No
				|_|_|_|_|\\\|_|_|\\\|_|_|	□Yes □No	|_|_|_|_|\\\|_|_|\\\|_|_|	□Yes □No
				|_|_|_|_|\\\|_|_|\\\|_|_|	□Yes □No	|_|_|_|_|\\\|_|_|\\\|_|_|	□Yes □No
				|_|_|_|_|\\\|_|_|\\\|_|_|	□Yes □No	|_|_|_|_|\\\|_|_|\\\|_|_|	□Yes □No
				|_|_|_|_|\\\|_|_|\\\|_|_|	□Yes □No	|_|_|_|_|\\\|_|_|\\\|_|_|	□Yes □No
				|_|_|_|_|\\\|_|_|\\\|_|_|	□Yes □No	|_|_|_|_|\\\|_|_|\\\|_|_|	□Yes □No
				|_|_|_|_|\\\|_|_|\\\|_|_|	□Yes □No	|_|_|_|_|\\\|_|_|\\\|_|_|	□Yes □No
				|_|_|_|_|\\\|_|_|\\\|_|_|	□Yes □No	|_|_|_|_|\\\|_|_|\\\|_|_|	□Yes □No

Notes：Non-drug treatment, a single dose filled in NA

Frequency: 1. Once a day (qd) twice a day (bid) 3. Three times a day (tid) 4. Four times a day (qid) 5. Once the other day (qod) 6. Once before bed (qn) 7. When necessary, use the following information: (prn)8.other_______________

Family history and a history of drug allergy

Family history： NO□ YES□ kinsfolk□□

Drug allergy history：NO□ YES□，anaphylactogen：______， ___________

					Page：
Approval Number	Random number □□	Treatment number □□	Patient name phonetic alphabet □□□□		Screening period

Case screening table

Menstrual general situation inquiries			
last menstrual period			
Average duration of menstrual period in last 3 months			
Expected time of next menstrual period			
**Inclusion criteria (if any item is selected "No", it cannot be included in the criteria)**	**Yes**	**No**	**Not checked**
1. Meet the diagnostic criteria of Chinese and Western medicine for primary dysmenorrhea			
2. Age between 16 and 35 years old			
3. The basic regularity of the menstrual cycle (28±7 days); the menstrual period is 4-7 days			
4.There was no other therapy for dysmenorrhea in one month before electroacupuncture therapy			
5.Willing to cooperate with the treatment operation, examination and efficacy evaluation, and did not participate in other clinical experiments			
6.Can provide detailed contact information, no short-term migration, can cooperate with the follow-up person			
7.The average pain visual analogue scale (VAS) for 3 consecutive menstrual cycles is ≥4cm.			
8.Informed consent form was signed			
**Exclusion criteria (if any item is selected "No", it cannot be included in the criteria)**	**Yes**	**No**	**Not checked**
1.Undiagnosed abnormal vaginal bleeding, endometrial polyps, uterine malformation, or endometrial hyperplasia			
2.He has polycystic ovary syndrome			
3.Have thrombosis, embolism, cerebrovascular disease or a history of coronary artery disease, or a tendency to thrombosis			
4.With mental illness or cognitive impairment, no scale evaluation content can be understood			
5.A history of complications or depression or medication for depression			

6.Severe heart, lung, liver, kidney disease; or blood, immune, or endocrine system diseases			
7.Infectious diseases			
8.Having malignancy or a history of malignancy or malignancy findings indicated			
9.Pregnancy or planned pregnancy or lactation within 1 year			
10.There was a history of jaundice or herpes herpes during pregnancy			
11.Participate in other clinical studies within 16 weeks before the date of informed consent			
12.Receive any of the following drugs within 12 weeks prior to the date of informed consent: GnRH analogues or testosterone derivatives; hormone preparations containing mainly progesterone or estrogen; estrogen antagonists or aromatase inhibitors, and ongoing treatment for other gynecological diseases			
13.Metal allergy or severe fear of the needle, electroacupuncture treatment can not be tolerated			
14.Electric needle local rupture, scar, cardiac pacemaker。			
15.According to the judgment of the researchers, there are other lesions or conditions that reduce the possibility of enrollment or complicated the enrollment, such as often changing working environment and unstable living environment, which are easy to cause lost to follow-up.			

Participant Signature :______________ Date :_____year___month___day

Investigator Signature : ___________ Date :______year___month___day

					Page：
Approval Number	Random number □□	Treatment number □□	Patient name phonetic alphabet □□□□		Screening period

Paste the place of the laboratory inspection report form

					Page:
**Approval Number**	**Random number** □□	**Treatment number** □□	**Patient name phonetic alphabet** □□□□		**Screening period**

Urinary pregnancy experiment

Date of examination|_|_|_|_|\|_|_|\|_|_|

Inspection result □Positive □Negative

Participant Signature :_____________ Date :_____year___month___day

Investigator Signature : ___________ Date : ______year month day

					Page：
Approval Number	Random number<br>□□	Treatment number<br>□□	Patient name phonetic alphabet<br>□□□□		Screening period

Gynecological B-ultrasound examination situation

Whether to conduct gynecological B-ultrasound examination：☐NO ☐YES

Whether the uterus morphology is normal： ☐NO ☐YES，Please explain__________

Palace size：__×____×____cm

Is the myometrium

Whether there are lesions in the uterus：☐NO ☐YES，The name of the lesion：☐hysteromyoma ☐adenomyosis ☐endometriosis Other________

Whether pelvic effusion：☐NO ☐YES Is there any pelvic inflammation：☐NO ☐YES

Ovarian ____×____×____cm

Is there an ovarian cyst：☐NO ☐ YES，________ Please explain

Whether the ovary is diseased：☐NO ☐YES， The name of the lesion

Diagnose：

Western medicine diagnosis：

Participant Signature :___________ Date :_____year___month___day

Investigator Signature : Date : year month day

					Page：
Approval Number	Random number □□	Treatment number □□	Patient name phonetic alphabet □□□□		Screening period

Gynecological B-ultrasound examination report paste place

					Page：
Approval Number	Random number □□	Treatment number □□	Patient name phonetic alphabet □□□□		Screening period

Visual Analogue Scale (VAS)

This scale is a scale line from 0-10 cm line length, right (0 cm) painless and right (10 cm) of the "most serious pain", the middle represents different degrees of pain, please according to the past three menstrual cycle dysmenorrhea, in the most can represent their pain to draw a cross line, select the highest value as the effective data.(Please keep one decimal place)

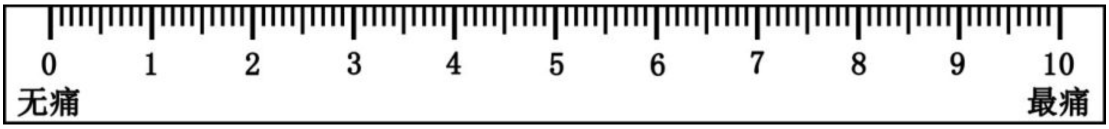

0 points: no pain;

1-3 points: mild pain, daily life and work are not affected;

4 to 6 points: moderate pain, daily life and work are affected;

7-9 points: severe pain, daily life and work and sleep are affected;

10 points: severe pain

Fill in for reference

Participant Signature :________________ Date :_____year___month___day

Investigator Signature :_______________ Date :_____year___month___day

					**Page：**
**Approval Number**	**Random number** □□	**Treatment number** □□	**Patient name phonetic alphabet** □□□□		**Screening period**

Numeric Pain Rating Scale（NRS）

The intensity of the current, best, and worst pain levels is expressed from 0 (no pain) to 10 (worst pain). Please rate your pain best based on your menstrual cramps in the past 3 menstrual cycles.

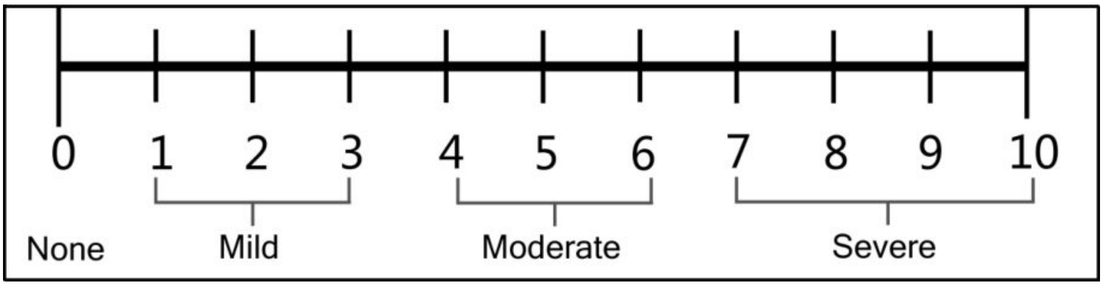

Participant Signature : ____________________Date : ____Year ____month ____day

Investigator Signature :____________________Date :____Year ____month ____day

					Page:
**Approval Number**	**Random number**<br>□□	**Treatment number**<br>□□	**Patient name phonetic alphabet**<br>□□□□		**Screening period**

COX Menstrual Symptom Scale (CMSS)

（Please fill in according to the dysmenorrhea situation of the last 3 menstrual cycles）

symptom	Duration (√)	Severity (√)	Score
Lower abdominal pain	0h <3h 3-7h 7-24h >24h<br>0 1 2 3 4 points	no mild Moderate very heavy<br>0 1 2 3 4 points	
Nausea	0h <3h 3-7h 7-24h >24h<br>0 1 2 3 4 points	no mild Moderate very heavy<br>0 1 2 3 4 points	
Vomit	0h <3h 3-7h 7-24h >24h<br>0 1 2 3 4 points	no mild Moderate very heavy<br>0 1 2 3 4 points	
Loss of appetite	0h <3h 3-7h 7-24h >24h<br>0 1 2 3 4 points	no mild Moderate very heavy<br>0 1 2 3 4 points	
Headache	0h <3h 3-7h 7-24h >24h<br>0 1 2 3 4 points	no mild Moderate very heavy<br>0 1 2 3 4 points	
Back (lumbosacral) Pain	0h <3h 3-7h 7-24h >24h<br>0 1 2 3 4 points	no mild Moderate very heavy<br>0 1 2 3 4 points	
Leg pain	0h <3h 3-7h 7-24h >24h<br>0 1 2 3 4 points	no mild Moderate very heavy<br>0 1 2 3 4 points	
Fatigue	0h <3h 3-7h 7-24h >24h<br>0 1 2 3 4 points	no mild Moderate very heavy<br>0 1 2 3 4 points	
Dizziness	0h <3h 3-7h 7-24h >24h<br>0 1 2 3 4 points	no mild Moderate very heavy<br>0 1 2 3 4 points	
Diarrhea	0h <3h 3-7h 7-24h >24h<br>0 1 2 3 4 points	no mild Moderate very heavy<br>0 1 2 3 4 points	
Complexion	0h <3h 3-7h 7-24h >24h<br>0 1 2 3 4 points	no mild Moderate very heavy<br>0 1 2 3 4 points	
Stomachache	0h <3h 3-7h 7-24h >24h<br>0 1 2 3 4 points	no mild Moderate very heavy<br>0 1 2 3 4 points	
Blushing	0h <3h 3-7h 7-24h >24h<br>0 1 2 3 4 points	no mild Moderate very heavy<br>0 1 2 3 4 points	
Insomnia	0h <3h 3-7h 7-24h >24h<br>0 1 2 3 4 points	no mild Moderate very heavy<br>0 1 2 3 4 points	
Body pain	0h <3h 3-7h 7-24h >24h<br>0 1 2 3 4 points	no mild Moderate very heavy<br>0 1 2 3 4 points	
Depression	0h <3h 3-7h 7-24h >24h<br>0 1 2 3 4 points	no mild Moderate very heavy<br>0 1 2 3 4 points	
Irritable	0h <3h 3-7h 7-24h >24h<br>0 1 2 3 4 points	no mild Moderate very heavy<br>0 1 2 3 4 points	
Neuroticism	0h <3h 3-7h 7-24h >24h<br>0 1 2 3 4 points	no mild Moderate very heavy<br>0 1 2 3 4 points	
Total score			

Participant signature :_______Investigator's signature :___________Date :_____year_____month____day

					Page:
**Approval Number**	**Random number**<br>□□	**Treatment number**<br>□□	**Patient name phonetic alphabet**<br>□□□□		**Screening period**

Observation table of TCM symptoms ofprimary dysmenorrhea

（Please fill in according to the dysmenorrhea situation of the last 3 menstrual cycles）

Name		Age		Telephone	
Address		Married or not		First visit time	
Recently used what medicine		Pasthistory	Course of disease		
Menarche time			Menstrual days		
Pain time	Before menstruation day	□	Pain duration		
	Period - days	□			
Menstrual week Expect	Come on time	□	Menstrual color	Light red	□
	More than 7 days in advance	□		Bright red	□
	More than 7 days delay	□		Dark red	□
	From time to time	□			
Menstruation quantity	Normal	□	Menstruation quality	Normal	□
	Too much	□		Rarefied	□
	Less	□		Thick	□
Painful area	Middle abdomen		Nature ofpain	Cold pain	□
				Pain	□
	Lower abdomen on both sides	□		Dull pain	□
	Pain in the waist	□		Tingling	□
				Burning pain	□
Concomitant disease shape	Breast tenderness	□	lumbosacral pain	□	
	Anal bulge	□	Dizziness and tinnitus	□	
	Feel sick and vomit	□	Headache	□	
	Diarrhea	□			

Participant signature :_______Investigator's signature :______Date :_year___month_day

					Page:
Approval Number	Random number □□	Treatment number □□	Patient name phonetic alphabet □□□□		Screening period

Self-Rating Anxiety Scale (SAS)

According to your actual situation in the last 3 months， tick a √ after the appropriate score.

Project	None or very little time	Small part time	Quite a bit of time	Most or all the time
1. I feel more nervous or anxious than usual	□ 1	□2	□ 3	□ 4
2. I am afraid for no reason	□ 1	□2	□ 3	□ 4
3. I am easily upset or panicked	□ 1	□2	□ 3	□ 4
4. I think I might be going crazy	□ 1	□2	□ 3	□ 4
5. I think everything is fine	□ 1	□2	□ 3	□ 4
6. My hands and feet tremble	□ 1	□2	□ 3	□ 4
7. I suffer from headaches, neck pain and back pain	□ 1	□2	□ 3	□ 4
8. I feel weak and tired easily	□ 1	□2	□ 3	□ 4
9. I feel calm and easy to sit still	□ 1	□2	□ 3	□ 4
10. I feel like my heart is beating fast	□ 1	□2	□ 3	□ 4
11. I was troubled by bouts of dizziness	□ 1	□2	□ 3	□ 4
12. I have an episode of fainting, or feel like I am going to pass out	□ 1	□2	□ 3	□ 4
13. I breathe in and out easily	□ 1	□2	□ 3	□ 4
14. My hands and feet are numb and tingling	□ 1	□2	□ 3	□ 4
15. I suffer from stomach pain and indigestion	□ 1	□2	□ 3	□ 4
16. I often have to urinate	□ 1	□2	□ 3	□ 4
17. My hands and feet are often dry and warm	□ 1	□2	□ 3	□ 4
18. My face is red and hot	□ 1	□2	□ 3	□ 4
19. I fall asleep easily and sleep well through the night	□ 1	□2	□ 3	□ 4
20. I have nightmares	□ 1	□2	□ 3	□ 4

Total score:|______|points

Investigator's signature: year month day

					Page:
Approval Number	Random number □□	Treatment number □□	Patient name phonetic alphabet □□□□		Screening period

Self-rating depressive scale(SDS)

According to your actual situation in the last 3 months， tick a √ after the appropriate score.

Project	None or very little time	Small part time	Quite a bit of time	Most or all the time
1. I feel sullen and depressed	□ 1	□ 2	□ 3	□ 4
2. I think the morning is the best of the day	□ 1	□ 2	□ 3	□ 4
3. I burst into tears or wanted to cry	□ 1	□ 2	□ 3	□ 4
4. I think I might be going crazy	□ 1	□ 2	□ 3	□ 4
5. I eat as much as usual	□ 1	□ 2	□ 3	□ 4
6. I am as happy as ever when I am in close contact with the opposite sex	□ 1	□ 2	□ 3	□ 4
7. I notice my weight is dropping	□ 1	□ 2	□ 3	□ 4
8. I have trouble with constipation	□ 1	□ 2	□ 3	□ 4
9. My heart beats faster than usual	□ 1	□ 2	□ 3	□ 4
10. I feel tired for no reason	□ 1	□ 2	□ 3	□ 4
11. My mind is as clear as usual	□ 1	□ 2	□ 3	□ 4
12. I don’t find it difficult to do things that I do often	□ 1	□ 2	□ 3	□ 4
13. I feel restless and can’t calm down	□ 1	□ 2	□ 3	□ 4
14. I have hope for the future	□ 1	□ 2	□ 3	□ 4
15. I am more agitated than usual	□ 1	□ 2	□ 3	□ 4
16. I find it easy to make decisions	□ 1	□ 2	□ 3	□ 4
17. I feel like a useful person, someone needs me	□ 1	□ 2	□ 3	□ 4
18. My life is very interesting	□ 1	□ 2	□ 3	□ 4
19. I think others would be better off if I died	□ 1	□ 2	□ 3	□ 4
20. I am still interested in the things I am usually interested in	□ 1	□ 2	□ 3	□ 4

Total score:|______|_| points

Investigator's signature: year month day

					Page:
Approval Number	Random number □□	Treatment number □□	Patient name phonetic alphabet □□□□		Screening period

Short form 36 questionnaire (SF-36)

1.Physiological function (PF: Physical Functioning)

(1) Heavy physical activity (e.g. running, lifting heavy objects, strenuous exercise, etc.)

□There are a lot of restrictions □A little restriction □No limit at all

(2) Moderate activities (such as moving tables, sweeping the floor, doing exercises, etc.)

□There are a lot of restrictions □A little restriction □No limit at all

(3) Portable daily necessities (such as grocery shopping, shopping, etc.)

□There are a lot of restrictions □A little restriction □No limit at all

(4) Go up some stairs

□There are a lot of restrictions □A little restriction □No limit at all

(5) go up the stairs

□There are a lot of restrictions □A little restriction □No limit at all

(6) Bend over, bend knees, squat

□There are a lot of restrictions □A little restriction □No limit at all

(7) Walk about 1500 meters

□There are a lot of restrictions □A little restriction □No limit at all

(8) Walk about 800 meters

□There are a lot of restrictions □A little restriction □No limit at all

(9) Walk about 100 meters

□There are a lot of restrictions □A little restriction □No limit at all

(10) bathe and dress yourself

□There are a lot of restrictions □A little restriction □No restrictions at all

A lot of restrictions A little restriction

					Page:
Approval Number	Random number □□	Treatment number □□	Patient name phonetic alphabet □□□□		Screening period

2.Physiological function

(1) Reduced time for work or other activities

□Yes □No

(2) Only part of what I wanted to do

□Yes □No

(3) Restricted types of work or activities you want to do

□Yes □No

(4) Difficulty completing work or other activities (eg, requiring extra effort)

□Yes □No

3.body pain

（1） Have you had any physical pain in the past four weeks ?

□No pain at □All There is very little pain □Have mild pain

□Moderate pain □Severe pain □Very severe pain

（2） In the past four weeks , has your physical pain interfered with your normal work (including work and household activities) ?

□No effect at all □Have a little influence □Moderate impact

□Have a greater impact □Have great influence

4.general health

（1） In general , Your health is

□very good □ Well □Good □ Fair □Poor

（2） I seem to get sick more easily than others

□Absolutely correct □Mostly correct □Not sure □Mostly wrong □Absolutely wrong

（3） I'm as healthy as anyone I know

□Absolutely correct □Mostly correct □ Not sure □ Mostly wrong □Bsolutely wrong

（4） I think my health is getting worse

□Absolutely correct □ Mostly correct □ Not sure □ Mostly wrong□Absolutely wrong

					Page:
Approval Number	Random number □□	Treatment number □□	Patient name phonetic alphabet □□□□		Screening period

（5） My health is very good

☑Absolutely correct □Mostly correct □Not sure □Mostly wrong □Absolutely wrong

5.Energy

（1） Do you feel life is full?

□All the time □Most of the time □More time

□Part of the time □Don't have this feeling

（2） Are you energetic?

□All the time □Most of the time □More time

□Part of the time □Don't have this feeling

（3） Are you feeling exhausted?

□All the time □Most of the time □More time

□Part of the time □Don't have this feeling

（4） Are you feeling tired?

□All the time □Most of the time □More time

□Part of the time □Don't have this feeling

6.Social function

（1） In the past four weeks, to what extent has your poor physical health or mood interfered with your normal social activities with family , friends, neighbors or groups ?

□No impact at all □Slight impact □Moderate impact

□Influence □Great influence

（2） your health limit your social activities (such as visiting family and friends) ?

□All the time □Most of the time □More time

□Part of the time □Don't have this feeling

7.emotional function

（1）Reduced time for work or other activities

□Yes □No

（2）Only part of what I wanted to do

□Yes □No

					Page:
**Approval Number**	**Random number** □□	**Treatment number** □□	**Patient name phonetic alphabet** □□□□		**Screening period**

（3）Doing work or other activities less carefully than usual

□Yes □No

1 . Mental Health

（1） Are you a nervous person?

□All the time □Most of the time □More time

□part of the time □Don't have this feeling

（2） Are you feeling down and nothing can cheer you up?

□All the time □Most of the time □More time

□Part of the time □Don't have this feeling

（3） Do you feel calm?

□All the time □Most of the time □More time

□part of the time □Don't have this feeling

（4） Are you feeling down?

□All the time □Most of the time □More time

□Part of the time □Don't have this feeling

（5） Are you a happy person?

□All the time □Most of the time □More time

□part of the time □Don't have this feeling

Participant Signature :_____________ Date :_____year___month___day

Investigator Signature : ___________ Date:______year___month___day

					Page：
Approval Number	Random number □□	Treatment number □□	Patient name phonetic alphabet □□□□		Screening period

Routine blood test

Date of examination：20|_|_|year|_|_|month|_|_|day							
Index	measured value	unit	Clinical significance judgment				remarks
			1	2	3	4	
Red-cell count（RBC）			□	□	□	□	
Leucocyte count（WBC）			□	□	□	□	
Platelet count（PLT）			□	□	□	□	
Hematocrit（HCT）			□	□	□	□	
Neutrophil count was performed（NEUT)			□	□	□	□	
Hemoglobin(Hb)			□	□	□	□	
**Note: Clinical significance judgment: 1 normal 2 abnormal no clinical significance 3 abnormal clinical significance 4 not checked**							

Blood biochemical

Date of examination：20|_|_|year|_|_|month|_|_|day							
Index	measured value	unit	Clinical significance judgment				remarks
			1	2	3	4	
Alanine aminotransferase（ALT)			□	□	□	□	
Aspartate aminotransferase (AST）			□	□	□	□	
Alkaline phosphatase （ALP）			□	□	□	□	
Total bilirubin （TBIL)			□	□	□	□	
Bilirubin direct(DBIL)			□	□	□	□	
Total protein (TP)			□	□	□	□	
Serum creatinine(SCr)			□	□	□	□	
Purine trione(UA)			□	□	□	□	
Fasting blood-glucose （FPG）			□	□	□	□	
Potassium （K）			□	□	□	□	
Sodium（Na）			□	□	□	□	
Chlorine（Cl）			□	□	□	□	
Calcium（Ca）			□	□	□	□	
**Note: Clinical significance judgment: 1 normal 2 abnormal no clinical significance 3 abnormal clinical significance 4 not checked**							

					Page:
Approval Number	Random number □□	Treatment number □□	Patient name phonetic alphabet □□□□		Screening period

Routine urine test

Date of examination：20|_|_|year|_|_|month|_|_|day							
Index	Measured value	Unit	Clinical significance judgment				Remarks
			1	2	3	4	
Urinary red blood cells（ERY)			□	□	□	□	
Urinary white blood cells (LEU）			□	□	□	□	
Urine protein （PRO）			□	□	□	□	
Urine glucose（GLU)			□	□	□	□	
Urine acetone bodies (KET)			□	□	□	□	
**Note: Clinical significance judgment: 1 normal 2 abnormal no clinical significance 3 abnormal clinical significance 4 not checked**							

Expectation value evaluation

Content: Generally speaking, do you think electroacupuncture has an effect in treating primary dysmenorrhea?

☐yes □no □dimness

Do you think that acupuncture will be helpful in improving your PD?

☐yes □no □dimness

Subjects signed: Date： year month day

Investigator signature： Date： year month day

					Page：
Approval Number	Random number □□	Treatment number □□	Patient name phonetic alphabet □□□□		stage of therapy (Week 4)

Urinary pregnancy experiment

Date of examination|_|_|_|_|\|_|_|\|_|_|

Inspection result □Positive □Negative

Participant Signature :_____________ Date :_____year___month___day

Investigator Signature : ___________ Date : ______year month day

					**Page：**
**Approval Number**	**Random number** □□	**Treatment number** □□	**Patient name phonetic alphabet** □□□□		**stage of therapy (Week 4)**

Visual Analogue Scale (VAS)

This scale is a scale line from the length of the 0-10 cm line, right (0 cm) painless and right (10 cm) of the "most serious pain", in the middle of different degrees of pain, please according to the past three menstrual cycle dysmenorrhea in the most can represent their pain to draw a cross line, select the highest value as the effective data.

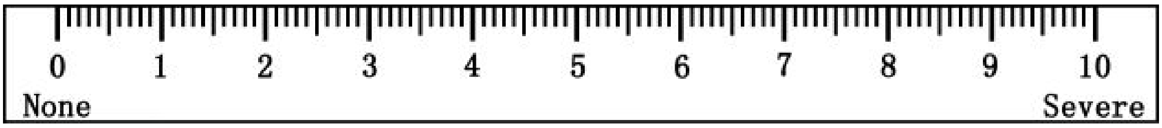

0 points: no pain;

1-3 points: mild pain, daily life and work are not affected;

4 to 6 points: moderate pain, daily life and work are affected;

7-9 points: severe pain, daily life and work and sleep are affected;

10 points: severe pain

Fill in for reference

Participant Signature :________________ Date :_____year___month___day

Investigator Signature :_______________ Date :_____year___month___day

					**Page：**
**Approval Number**	**Random number** □□	**Treatment number** □□	**Patient name phonetic alphabet** □□□□		**stage of therapy (Week 4)**

Numeric Pain Rating Scale（NRS）

The intensity of the current, best, and worst pain levels is expressed from 0 (no pain) to 10 (worst pain). Please rate your pain according on your latest menstrual cycle.

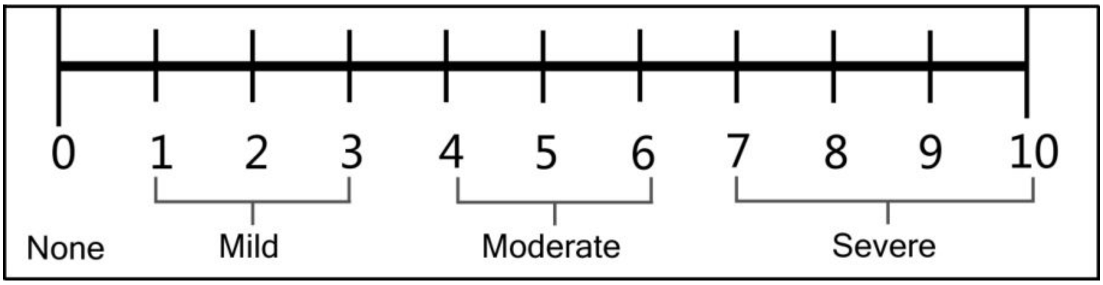

Participant Signature : ____________________Date : ____Year ____month ___day

Investigator Signature :___________________Date :____Year ____month ___day

					Page：
Approval Number	Random number □□	Treatment number □□	Patient name phonetic alphabet □□□□		stage of therapy (Week 4)

COX Menstrual Symptom Scale (CMSS)

(Please fill in according to the last menstrual cycle)

symptom	Duration (√)	Severity (√)	Score
Lower abdominal pain	0h <3h 3-7h 7-24h >24h<br>0 1 2 3 4 points	no mild Moderate very heavy<br>0 1 2 3 4 points	
Nausea	0h <3h 3-7h 7-24h >24h<br>0 1 2 3 4 points	no mild Moderate very heavy<br>0 1 2 3 4 points	
Vomit	0h <3h 3-7h 7-24h >24h<br>0 1 2 3 4 points	no mild Moderate very heavy<br>0 1 2 3 4 points	
Loss of appetite	0h <3h 3-7h 7-24h >24h<br>0 1 2 3 4 points	no mild Moderate very heavy<br>0 1 2 3 4 points	
Headache	0h <3h 3-7h 7-24h >24h<br>0 1 2 3 4 points	no mild Moderate very heavy<br>0 1 2 3 4 points	
Back (lumbosacral) Pain	0h <3h 3-7h 7-24h >24h<br>0 1 2 3 4 points	no mild Moderate very heavy<br>0 1 2 3 4 points	
Leg pain	0h <3h 3-7h 7-24h >24h<br>0 1 2 3 4 points	no mild Moderate very heavy<br>0 1 2 3 4 points	
Fatigue	0h <3h 3-7h 7-24h >24h<br>0 1 2 3 4 points	no mild Moderate very heavy<br>0 1 2 3 4 points	
Dizziness	0h <3h 3-7h 7-24h >24h<br>0 1 2 3 4 points	no mild Moderate very heavy<br>0 1 2 3 4 points	
Diarrhea	0h <3h 3-7h 7-24h >24h<br>0 1 2 3 4 points	no mild Moderate very heavy<br>0 1 2 3 4 points	
Complexion	0h <3h 3-7h 7-24h >24h<br>0 1 2 3 4 points	no mild Moderat very heavy<br>0 1 e 2 3 4 points	
Stomachache	0h <3h 3-7h 7-24h >24h<br>0 1 2 3 4 points	no mild Moderate very heavy<br>0 1 2 3 4 points	
Blushing	0h <3h 3-7h 7-24h >24h<br>0 1 2 3 4 points	no mild Moderate very heavy<br>0 1 2 3 4 points	
Insomnia	0h <3h 3-7h 7-24h >24h<br>0 1 2 3 4 points	no mild Moderate very heavy<br>0 1 2 3 4 points	
Body pain	0h <3h 3-7h 7-24h >24h<br>0 1 2 3 4 points	no mild Moderate very heavy<br>0 1 2 3 4 points	
Depression	0h <3h 3-7h 7-24h >24h<br>0 1 2 3 4 points	no mild Moderate very heavy<br>0 1 2 3 4 points	
Irritable	0h <3h 3-7h 7-24h >24h<br>0 1 2 3 4 points	no mild Moderate very heavy<br>0 1 2 3 4 points	
Neuroticism	0h <3h 3-7h 7-24h >24h<br>0 1 2 3 4 points	no mild Moderate very heavy<br>0 1 2 3 4 points	
Total score			

Participant signature :_______Investigator's signature :__________Date :_____year_____month____day

					Page:
Approval Number	Random number □□	Treatment number □□	Patient name phonetic alphabet □□□□		stage of therapy (Week 4)

Observation table of TCM symptoms ofprimary dysmenorrhea

(Please fill in according to the latest menstrual cycle of dysmenorrhea)

Name		Age		Telephone	
Address		Married or not		First visit time	
Recently used what medicine		Pasthistory	Course of disease		
Menarche time			Menstrual days		
Pain time	Before menstruation day	□	Pain duration		
	Period - days	□			
Menstrual week Expect	Come on time	□	Menstrual color	Light red	□
	More than 7 days in advance	□		Bright red	□
	More than 7 days delay	□		Dark red	□
	From time to time	□			
Menstruation quantity	Normal	□	Menstruation quality	Normal	□
	Too much	□		Rarefied	□
	Less	□		Thick	□
Painful area	Middle abdomen		Nature ofpain	Cold pain	□
				Pain	□
	Lower abdomen on both sides	□		Dull pain	□
	Pain in the waist	□		Tingling	□
				Burning pain	□
Concomitant disease shape	Breast tenderness	□	lumbosacral pain	□	
	Anal bulge	□	Dizziness and tinnitus	□	
	Feel sick and vomit	□	Headache	□	
	Diarrhea	□			

Participant signature :_______Investigator's signature :______Date :_year___month__day

					Page:
**Approval Number**	**Random number** □□	**Treatment number** □□	**Patient name phonetic alphabet** □□□□		**stage of therapy (Week 4)**

Self-Rating Anxiety Scale (SAS)

According to your actual situation in the last month， tick a √ after the appropriate score.

Project	None or very little time	Small part time	Quite a bit of time	Most or all the time
1. I feel more nervous or anxious than usual	□ 1	□2	□ 3	□ 4
2. I am afraid for no reason	□ 1	□2	□ 3	□ 4
3. I am easily upset or panicked	□ 1	□2	□ 3	□ 4
4. I think I might be going crazy	□ 1	□2	□ 3	□ 4
5. I think everything is fine	□ 1	□2	□ 3	□ 4
6. My hands and feet tremble	□ 1	□2	□ 3	□ 4
7. I suffer from headaches, neck pain and back pain	□ 1	□2	□ 3	□ 4
8. I feel weak and tired easily	□ 1	□2	□ 3	□ 4
9. I feel calm and easy to sit still	□ 1	□2	□ 3	□ 4
10. I feel like my heart is beating fast	□ 1	□2	□ 3	□ 4
11. I was troubled by bouts of dizziness	□ 1	□2	□ 3	□ 4
12. I have an episode of fainting, or feel like I am going to pass out	□ 1	□2	□ 3	□ 4
13. I breathe in and out easily	□ 1	□2	□ 3	□ 4
14. My hands and feet are numb and tingling	□ 1	□2	□ 3	□ 4
15. I suffer from stomach pain and indigestion	□ 1	□2	□ 3	□ 4
16. I often have to urinate	□ 1	□2	□ 3	□ 4
17. My hands and feet are often dry and warm	□ 1	□2	□ 3	□ 4
18. My face is red and hot	□ 1	□2	□ 3	□ 4
19. I fall asleep easily and sleep well through the night	□ 1	□2	□ 3	□ 4
20. I have nightmares	□ 1	□2	□ 3	□ 4

Total score:|______| points

Investigator's signature: year month day

					Page:
Approval Number	Random number □□	Treatment number □□	Patient name phonetic alphabet □□□□		stage of therapy (Week 4)

Self-rating depressive scale(SDS)

According to your actual situation in the last month， tick a √ after the appropriate score.

Project	None or very little time	Small part time	Quite a bit of time	Most or all the time
1. I feel sullen and depressed	□ 1	□ 2	□ 3	□ 4
2. I think the morning is the best of the day	□ 1	□ 2	□ 3	□ 4
3. I burst into tears or wanted to cry	□ 1	□ 2	□ 3	□ 4
4. I think I might be going crazy	□ 1	□ 2	□ 3	□ 4
5. I eat as much as usual	□ 1	□ 2	□ 3	□ 4
6. I am as happy as ever when I am in close contact with the opposite sex	□ 1	□ 2	□ 3	□ 4
7. I notice my weight is dropping	□ 1	□ 2	□ 3	□ 4
8. I have trouble with constipation	□ 1	□ 2	□ 3	□ 4
9. My heart beats faster than usual	□ 1	□ 2	□ 3	□ 4
10. I feel tired for no reason	□ 1	□ 2	□ 3	□ 4
11. My mind is as clear as usual	□ 1	□ 2	□ 3	□ 4
12. I don’t find it difficult to do things that I do often	□ 1	□ 2	□ 3	□ 4
13. I feel restless and can’t calm down	□ 1	□ 2	□ 3	□ 4
14. I have hope for the future	□ 1	□ 2	□ 3	□ 4
15. I am more agitated than usual	□ 1	□ 2	□ 3	□ 4
16. I find it easy to make decisions	□ 1	□ 2	□ 3	□ 4
17. I feel like a useful person, someone needs me	□ 1	□ 2	□ 3	□ 4
18. My life is very interesting	□ 1	□ 2	□ 3	□ 4
19. I think others would be better off if I died	□ 1	□ 2	□ 3	□ 4
20. I am still interested in the things I am usually interested in	□ 1	□ 2	□ 3	□ 4

Total score:|______|_| points

Investigator's signature: year month day

					Page：
Approval Number	Random number □□	Treatment number □□	Patient name phonetic alphabet □□□□		stage of therapy (Week 4)

Short form 36 questionnaire (SF-36)

According to your actual situation in the last month， tick a √ after the appropriate score.

1.Physiological function (PF: Physical Functioning)

(1)	Heavy physical activity (e.g. running, lifting heavy objects, strenuous exercise, etc.)	
(2)	□There are a lot of restrictions □A little restriction<br>Moderate activities (such as moving tables, sweeping the floor, doing exercises, etc.)	□No limit at all
(3)	□There are a lot of restrictions □A little restriction<br>Portable daily necessities (such as grocery shopping, shopping, etc.)	□No limit at all
(4)	□There are a lot of restrictions □A little restriction<br>Go up some stairs	□No limit at all
(5)	□There are a lot of restrictions □A little restriction<br>go up the stairs	□No limit at all
(6)	□There are a lot of restrictions □A little restriction<br>Bend over, bend knees, squat	□No limit at all
(7)	□There are a lot of restrictions □A little restriction<br>Walk about 1500 meters	□No limit at all
(8)	□There are a lot of restrictions □A little restriction<br>Walk about 800 meters	□No limit at all
(9)	□There are a lot of restrictions □A little restriction<br>Walk about 100 meters	□No limit at all
(10)	□There are a lot of restrictions □A little restriction<br>bathe and dress yourself<br>A lot of restrictions A little restriction	□No restrictions at all

					Page：
Approval Number	Random number □□	Treatment number □□	Patient name phonetic alphabet □□□□		stage of therapy (Week 4)

2.Physiological function

(5) Reduced time for work or other activities

□Yes □No

(6) Only part of what I wanted to do

□Yes □No

(7) Restricted types of work or activities you want to do

□Yes □No

(8) Difficulty completing work or other activities (eg, requiring extra effort)

□Yes □No

3.body pain

（3） Have you had any physical pain in the past four weeks ?

□No pain at □All There is very little pain □Have mild pain

□Moderate pain □Severe pain □Very severe pain

（4） In the past four weeks , has your physical pain interfered with your normal work (including work and household activities) ?

□No effect at all □Have a little influence □Moderate impact

□Have a greater impact □Have great influence

4.general health

（6） In general , Your health is

□very good □ Well □Good □ Fair □Poor

（7） I seem to get sick more easily than others

□Absolutely correct □Mostly correct □Not sure □Mostly wrong □Absolutely wrong

（8） I'm as healthy as anyone I know

□Absolutely correct □Mostly correct □ Not sure □ Mostly wrong □Bsolutely wrong

（9） I think my health is getting worse

□Absolutely correct □ Mostly correct □ Not sure □ Mostly wrong□Absolutely wrong

					Page:
**Approval Number**	**Random number** □□	**Treatment number** □□	**Patient name phonetic alphabet** □□□□		**stage of therapy (Week 4)**

（10） My health is very good

☑Absolutely correct □Mostly correct □Not sure □Mostly wrong □Absolutely wrong

7.Energy

（5） Do you feel life is full?

□All the time □Most of the time □More time

□Part of the time □Don't have this feeling

（6） Are you energetic?

□All the time □Most of the time □More time

□Part of the time □Don't have this feeling

（7） Are you feeling exhausted?

□All the time □Most of the time □More time

□Part of the time □Don't have this feeling

（8） Are you feeling tired?

□All the time □Most of the time □More time

□Part of the time □Don't have this feeling

8.Social function

（3） In the past four weeks, to what extent has your poor physical health or mood interfered with your normal social activities with family , friends, neighbors or groups ?

□No impact at all □Slight impact □Moderate impact

□Influence □Great influence

（4） your health limit your social activities (such as visiting family and friends) ?

□All the time □Most of the time □More time

□Part of the time □Don't have this feeling

7.emotional function

（1）Reduced time for work or other activities

□Yes □No

（2）Only part of what I wanted to do

□Yes □No

Approval Number	Random number □□	Treatment number □□	Patient name phonetic alphabet □□□□		stage of therapy (Week 4)

（3）Doing work or other activities less carefully than usual

□Yes □No

1 . Mental Health

（6） Are you a nervous person?

□All the time □Most of the time □More time

□part of the time □Don't have this feeling

（7） Are you feeling down and nothing can cheer you up?

□All the time □Most of the time □More time

□Part of the time □Don't have this feeling

（8） Do you feel calm?

□All the time □Most of the time □More time

□part of the time □Don't have this feeling

（9） Are you feeling down?

□All the time □Most of the time □More time

□Part of the time □Don't have this feeling

（10） Are you a happy person?

□All the time □Most of the time □More time

□part of the time □Don't have this feeling

Participant Signature :_____________ Date :_____year___month___day

Investigator Signature : ___________ Date:______year___month___day

					Page：
**Approval Number**	**Random number** □□	**Treatment number** □□	**Patient name phonetic alphabet** □□□□		**stage of therapy (Week 8)**

Urinary pregnancy experiment

Date of examination|_|_|_|_|\|_|_|\|_|_|

Inspection result □Positive □Negative

Participant Signature :_____________ Date :_____year___month___day

Investigator Signature : ___________ Date : ______year month day

					**Page：**
**Approval Number**	**Random number** □□	**Treatment number** □□	**Patient name phonetic alphabet** □□□□		**stage of therapy (Week 8)**

Visual Analogue Scale (VAS)

This scale is a scale line from the length of the 0-10 cm line, right (0 cm) painless and right (10 cm) of the "most serious pain", in the middle of different degrees of pain, please according to the past three menstrual cycle dysmenorrhea in the most can represent their pain to draw a cross line, select the highest value as the effective data.

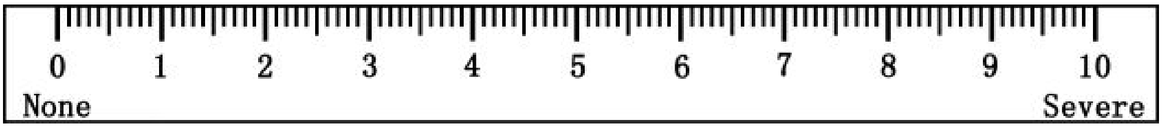

0 points: no pain;

1-3 points: mild pain, daily life and work are not affected;

4 to 6 points: moderate pain, daily life and work are affected;

7-9 points: severe pain, daily life and work and sleep are affected;

10 points: severe pain

Fill in for reference

Participant Signature :________________ Date :_____year___month___day

Investigator Signature :_______________ Date :_____year___month___day

					Page：
**Approval Number**	**Random number** □□	**Treatment number** □□	**Patient name phonetic alphabet** □□□□		**stage of therapy (Week 8)**

Numeric Pain Rating Scale（NRS）

The intensity of the current, best, and worst pain levels is expressed from 0 (no pain) to 10 (worst pain). Please rate your pain according on your latest menstrual cycle.

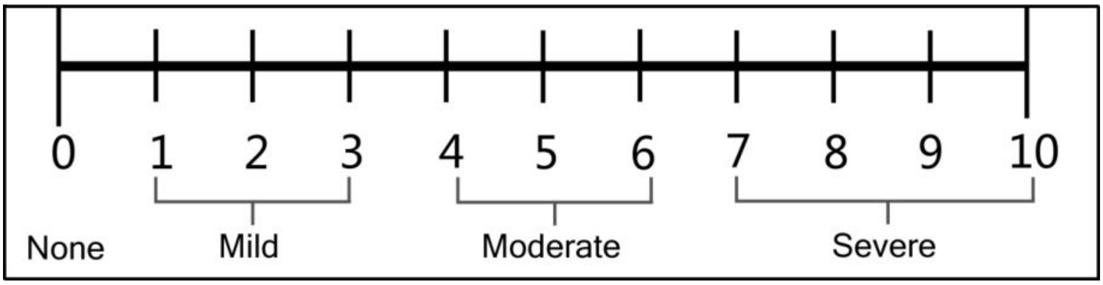

Participant Signature : ____________________Date : ____Year ____month ____day

Investigator Signature :___________________Date :____Year ____month ____day

					Page:
Approval Number	Random number □□	Treatment number □□	Patient name phonetic alphabet □□□□		stage of therapy (Week 8)

COX Menstrual Symptom Scale (CMSS)

(Please fill in according to the last menstrual cycle)

symptom	Duration (√)	Severity (√)	Score
Lower abdominal pain	0h <3h 3-7h 7-24h >24h<br>0 1 2 3 4 points	no mild Moderate very heavy<br>0 1 2 3 4 points	
Nausea	0h <3h 3-7h 7-24h >24h<br>0 1 2 3 4 points	no mild Moderate very heavy<br>0 1 2 3 4 points	
Vomit	0h <3h 3-7h 7-24h >24h<br>0 1 2 3 4 points	no mild Moderate very heavy<br>0 1 2 3 4 points	
Loss of appetite	0h <3h 3-7h 7-24h >24h<br>0 1 2 3 4 points	no mild Moderate very heavy<br>0 1 2 3 4 points	
Headache	0h <3h 3-7h 7-24h >24h<br>0 1 2 3 4 points	no mild Moderate very heavy<br>0 1 2 3 4 points	
Back (lumbosacral) Pain	0h <3h 3-7h 7-24h >24h<br>0 1 2 3 4 points	no mild Moderate very heavy<br>0 1 2 3 4 points	
Leg pain	0h <3h 3-7h 7-24h >24h<br>0 1 2 3 4 points	no mild Moderate very heavy<br>0 1 2 3 4 points	
Fatigue	0h <3h 3-7h 7-24h >24h<br>0 1 2 3 4 points	no mild Moderate very heavy<br>0 1 2 3 4 points	
Dizziness	0h <3h 3-7h 7-24h >24h<br>0 1 2 3 4 points	no mild Moderate very heavy<br>0 1 2 3 4 points	
Diarrhea	0h <3h 3-7h 7-24h >24h<br>0 1 2 3 4 points	no mild Moderate very heavy<br>0 1 2 3 4 points	
Complexion	0h <3h 3-7h 7-24h >24h<br>0 1 2 3 4 points	no mild Moderate very heavy<br>0 1 2 3 4 points	
Stomachache	0h <3h 3-7h 7-24h >24h<br>0 1 2 3 4 points	no mild Moderate very heavy<br>0 1 2 3 4 points	
Blushing	0h <3h 3-7h 7-24h >24h<br>0 1 2 3 4 points	no mild Moderate very heavy<br>0 1 2 3 4 points	
Insomnia	0h <3h 3-7h 7-24h >24h<br>0 1 2 3 4 points	no mild Moderate very heavy<br>0 1 2 3 4 points	
Body pain	0h <3h 3-7h 7-24h >24h<br>0 1 2 3 4 points	no mild Moderate very heavy<br>0 1 2 3 4 points	
Depression	0h <3h 3-7h 7-24h >24h<br>0 1 2 3 4 points	no mild Moderate very heavy<br>0 1 2 3 4 points	
Irritable	0h <3h 3-7h 7-24h >24h<br>0 1 2 3 4 points	no mild Moderate very heavy<br>0 1 2 3 4 points	
Neuroticism	0h <3h 3-7h 7-24h >24h<br>0 1 2 3 4 points	no mild Moderate very heavy<br>0 1 2 3 4 points	
Total score			

Participant signature :_______Investigator's signature :__________Date :_____year_____month____day

					Page:
**Approval Number**	**Random number** □□	**Treatment number** □□	**Patient name phonetic alphabet** □□□□		**stage of therapy (Week 8)**

Observation table of TCM symptoms ofprimary dysmenorrhea

(Please fill in according to the latest menstrual cycle of dysmenorrhea)

Name		Age		Telephone	
Address		Married or not		First visit time	
Recently used what medicine		Pasthistory		Course of disease	
Menarche time				Menstrual days	
Pain time	Before menstruation day	□	Pain duration		
	Period - days	□			
Menstrual week Expect	Come on time	□	Menstrual color	Light red	□
	More than 7 days in advance	□		Bright red	□
	More than 7 days delay	□		Dark red	□
	From time to time	□			
Menstruation quantity	Normal	□	Menstruation quality	Normal	□
	Too much	□		Rarefied	□
	Less	□		Thick	□
Painful area	Middle abdomen		Nature ofpain	Cold pain	□
				Pain	□
	Lower abdomen on both sides	□		Dull pain	□
	Pain in the waist	□		Tingling	□
				Burning pain	□
Concomitant disease shape	Breast tenderness	□	lumbosacral pain	□	
	Anal bulge	□	Dizziness and tinnitus	□	
	Feel sick and vomit	□	Headache	□	
	Diarrhea	□			

Participant signature :_______Investigator's signature :______Date :_year___month_day

					Page：
**Approval Number**	**Random number** □□	**Treatment number** □□	**Patient name phonetic alphabet** □□□□		**stage of therapy (Week 8)**

Self-Rating Anxiety Scale (SAS)

According to your actual situation in the last month， tick a √ after the appropriate score.

Project	None or very little time	Small part time	Quite a bit of time	Most or all the time
1. I feel more nervous or anxious than usual	□ 1	□2	□ 3	□ 4
2. I am afraid for no reason	□ 1	□2	□ 3	□ 4
3. I am easily upset or panicked	□ 1	□2	□ 3	□ 4
4. I think I might be going crazy	□ 1	□2	□ 3	□ 4
5. I think everything is fine	□ 1	□2	□ 3	□ 4
6. My hands and feet tremble	□ 1	□2	□ 3	□ 4
7. I suffer from headaches, neck pain and back pain	□ 1	□2	□ 3	□ 4
8. I feel weak and tired easily	□ 1	□2	□ 3	□ 4
9. I feel calm and easy to sit still	□ 1	□2	□ 3	□ 4
10. I feel like my heart is beating fast	□ 1	□2	□ 3	□ 4
11. I was troubled by bouts of dizziness	□ 1	□2	□ 3	□ 4
12. I have an episode of fainting, or feel like I am going to pass out	□ 1	□2	□ 3	□ 4
13. I breathe in and out easily	□ 1	□2	□ 3	□ 4
14. My hands and feet are numb and tingling	□ 1	□2	□ 3	□ 4
15. I suffer from stomach pain and indigestion	□ 1	□2	□ 3	□ 4
16. I often have to urinate	□ 1	□2	□ 3	□ 4
17. My hands and feet are often dry and warm	□ 1	□2	□ 3	□ 4
18. My face is red and hot	□ 1	□2	□ 3	□ 4
19. I fall asleep easily and sleep well through the night	□ 1	□2	□ 3	□ 4
20. I have nightmares	□ 1	□2	□ 3	□ 4

Total score:|______| points

Investigator's signature: year month day

					Page:
**Approval Number**	**Random number** □□	**Treatment number** □□	**Patient name phonetic alphabet** □□□□		**stage of therapy (Week 8)**

Self-rating depressive scale(SDS)

According to your actual situation in the last month， tick a √ after the appropriate score.

Project	None or very little time	Small part time	Quite a bit of time	Most or all the time
1. I feel sullen and depressed	□ 1	□ 2	□ 3	□ 4
2. I think the morning is the best of the day	□ 1	□ 2	□ 3	□ 4
3. I burst into tears or wanted to cry	□ 1	□ 2	□ 3	□ 4
4. I think I might be going crazy	□ 1	□ 2	□ 3	□ 4
5. I eat as much as usual	□ 1	□ 2	□ 3	□ 4
6. I am as happy as ever when I am in close contact with the opposite sex	□ 1	□ 2	□ 3	□ 4
7. I notice my weight is dropping	□ 1	□ 2	□ 3	□ 4
8. I have trouble with constipation	□ 1	□ 2	□ 3	□ 4
9. My heart beats faster than usual	□ 1	□ 2	□ 3	□ 4
10. I feel tired for no reason	□ 1	□ 2	□ 3	□ 4
11. My mind is as clear as usual	□ 1	□ 2	□ 3	□ 4
12. I don’t find it difficult to do things that I do often	□ 1	□ 2	□ 3	□ 4
13. I feel restless and can’t calm down	□ 1	□ 2	□ 3	□ 4
14. I have hope for the future	□ 1	□ 2	□ 3	□ 4
15. I am more agitated than usual	□ 1	□ 2	□ 3	□ 4
16. I find it easy to make decisions	□ 1	□ 2	□ 3	□ 4
17. I feel like a useful person, someone needs me	□ 1	□ 2	□ 3	□ 4
18. My life is very interesting	□ 1	□ 2	□ 3	□ 4
19. I think others would be better off if I died	□ 1	□ 2	□ 3	□ 4
20. I am still interested in the things I am usually interested in	□ 1	□ 2	□ 3	□ 4

Total score:|______|_| points

Investigator's signature: year month day

					Page：
Approval Number	Random number □□	Treatment number □□	Patient name phonetic alphabet □□□□		stage of therapy (Week 8)

Short form 36 questionnaire (SF-36)

According to your actual situation in the last month， tick a √ after the appropriate score.

1.Physiological function (PF: Physical Functioning)

(1) Heavy physical activity (e.g. running, lifting heavy objects, strenuous exercise, etc.)

□There are a lot of restrictions □A little restriction □No limit at all

(2) Moderate activities (such as moving tables, sweeping the floor, doing exercises, etc.)

□There are a lot of restrictions □A little restriction □No limit at all

(3) Portable daily necessities (such as grocery shopping, shopping, etc.)

□There are a lot of restrictions □A little restriction □No limit at all

(4) Go up some stairs

□There are a lot of restrictions □A little restriction □No limit at all

(5) go up the stairs

□There are a lot of restrictions □A little restriction □No limit at all

(6) Bend over, bend knees, squat

□There are a lot of restrictions □A little restriction □No limit at all

(7) Walk about 1500 meters

□There are a lot of restrictions □A little restriction □No limit at all

(8) Walk about 800 meters

□There are a lot of restrictions □A little restriction □No limit at all

(9) Walk about 100 meters

□There are a lot of restrictions □A little restriction □No limit at all

(10) bathe and dress yourself

□There are a lot of restrictions □A little restriction □No restrictions at all

A lot of restrictions A little restriction

					Page：
Approval Number	Random number □□	Treatment number □□	Patient name phonetic alphabet □□□□		stage of therapy (Week 8)

2.Physiological function

(9) Reduced time for work or other activities

□Yes □No

(10)Only part of what I wanted to do

□Yes □No

(11)Restricted types of work or activities you want to do

□Yes □No

(12)Difficulty completing work or other activities (eg, requiring extra effort)

□Yes □No

3.body pain

（5） Have you had any physical pain in the past four weeks ?

□No pain at □All There is very little pain □Have mild pain

□Moderate pain □Severe pain □Very severe pain

（6） In the past four weeks , has your physical pain interfered with your normal work (including work and household activities) ?

□No effect at all □Have a little influence □Moderate impact

□Have a greater impact □Have great influence

4.general health

（11） In general , Your health is

□very good □ Well □Good □ Fair □Poor

（12） I seem to get sick more easily than others

□Absolutely correct □Mostly correct □Not sure □Mostly wrong □Absolutely wrong

（13） I'm as healthy as anyone I know

□Absolutely correct □Mostly correct □ Not sure □ Mostly wrong □Bsolutely wrong

（14） I think my health is getting worse

□Absolutely correct □ Mostly correct □ Not sure □ Mostly wrong□Absolutely wrong

					Page：
Approval Number	Random number □□	Treatment number □□	Patient name phonetic alphabet □□□□		stage of therapy (Week 8)

(15) My health is very good

☑Absolutely correct □Mostly correct □Not sure □Mostly wrong □Absolutely wrong

9.Energy

(9) Do you feel life is full?

□All the time □Most of the time □More time

□Part of the time □Don't have this feeling

(10) Are you energetic?

□All the time □Most of the time □More time

□Part of the time □Don't have this feeling

(11) Are you feeling exhausted?

□All the time □Most of the time □More time

□Part of the time □Don't have this feeling

(12) Are you feeling tired?

□All the time □Most of the time □More time

□Part of the time □Don't have this feeling

10. Social function

(5) In the past four weeks, to what extent has your poor physical health or mood interfered with your normal social activities with family , friends, neighbors or groups ?

□No impact at all □Slight impact □Moderate impact

□Influence □Great influence

(6) your health limit your social activities (such as visiting family and friends) ?

□All the time □Most of the time □More time

□Part of the time □Don't have this feeling

7.emotional function

(1) Reduced time for work or other activities

□Yes □No

(2) Only part of what I wanted to do

□Yes □No

					Page:
Approval Number	Random number<br>□□	Treatment number<br>□□	Patient name phonetic alphabet<br>□□□□		stage of therapy (Week 8)

（3）Doing work or other activities less carefully than usual

□Yes □No

1 . Mental Health

（11） Are you a nervous person?

□All the time □Most of the time □More time

□part of the time □Don't have this feeling

（12） Are you feeling down and nothing can cheer you up?

□All the time □Most of the time □More time

□Part of the time □Don't have this feeling

（13） Do you feel calm?

□All the time □Most of the time □More time

□part of the time □Don't have this feeling

（14） Are you feeling down?

□All the time □Most of the time □More time

□Part of the time □Don't have this feeling

（15） Are you a happy person?

□All the time □Most of the time □More time

□part of the time □Don't have this feeling

Participant Signature :______________ Date :______year___month___day

Investigator Signature : ___________ Date:______year___month___day

					Page：
**Approval Number**	**Random number** □□	**Treatment number** □□	**Patient name phonetic alphabet** □□□□		**stage of therapy (Week 12)**

Visual Analogue Scale (VAS)

This scale is a scale line from the length of the 0-10 cm line, right (0 cm) painless and right (10 cm) of the "most serious pain", in the middle of different degrees of pain, please according to the past three menstrual cycle dysmenorrhea in the most can represent their pain to draw a cross line, select the highest value as the effective data.

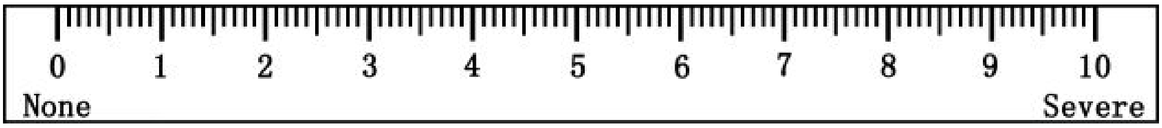

0 points: no pain;

1-3 points: mild pain, daily life and work are not affected;

4 to 6 points: moderate pain, daily life and work are affected;

7-9 points: severe pain, daily life and work and sleep are affected;

10 points: severe pain

Fill in for reference

Participant Signature :________________ Date :_____year___month___day

Investigator Signature :_______________ Date :_____year___month___day

					**Page：**
**Approval Number**	**Random number** □□	**Treatment number** □□	**Patient name phonetic alphabet** □□□□		**stage of therapy (Week 12)**

Numeric Pain Rating Scale（NRS）

The intensity of the current, best, and worst pain levels is expressed from 0 (no pain) to 10 (worst pain). Please rate your pain according on your latest menstrual cycle.

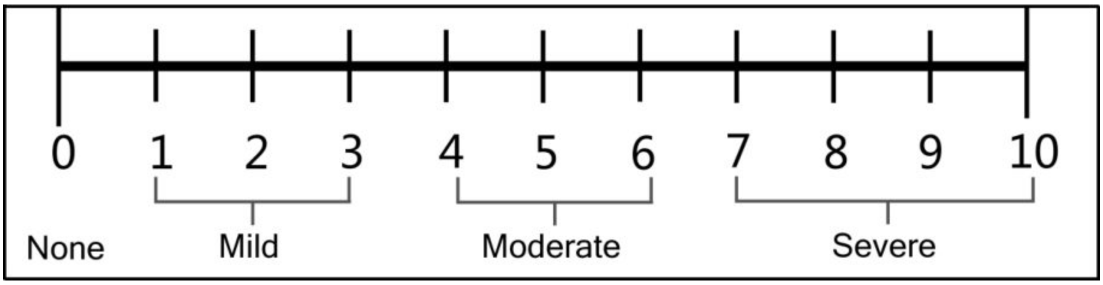

Participant Signature : ____________________Date : ____Year ____month ___day

Investigator Signature :___________________Date :____Year ____month ___day

					Page:
Approval Number	Random number<br>□□	Treatment number<br>□□	Patient name phonetic alphabet<br>□□□□		stage of therapy (Week 12)

COX Menstrual Symptom Scale (CMSS)

(Please fill in according to the last menstrual cycle)

symptom	Duration (√)	Severity (√)	Score
Lower abdominal pain	0h <3h 3-7h 7-24h >24h<br>0 1 2 3 4 points	no mild Moderate very heavy<br>0 1 2 3 4 points	
Nausea	0h <3h 3-7h 7-24h >24h<br>0 1 2 3 4 points	no mild Moderate very heavy<br>0 1 2 3 4 points	
Vomit	0h <3h 3-7h 7-24h >24h<br>0 1 2 3 4 points	no mild Moderate very heavy<br>0 1 2 3 4 points	
Loss of appetite	0h <3h 3-7h 7-24h >24h<br>0 1 2 3 4 points	no mild Moderate very heavy<br>0 1 2 3 4 points	
Headache	0h <3h 3-7h 7-24h >24h<br>0 1 2 3 4 points	no mild Moderate very heavy<br>0 1 2 3 4 points	
Back (lumbosacral) Pain	0h <3h 3-7h 7-24h >24h<br>0 1 2 3 4 points	no mild Moderate very heavy<br>0 1 2 3 4 points	
Leg pain	0h <3h 3-7h 7-24h >24h<br>0 1 2 3 4 points	no mild Moderate very heavy<br>0 1 2 3 4 points	
Fatigue	0h <3h 3-7h 7-24h >24h<br>0 1 2 3 4 points	no mild Moderate very heavy<br>0 1 2 3 4 points	
Dizziness	0h <3h 3-7h 7-24h >24h<br>0 1 2 3 4 points	no mild Moderate very heavy<br>0 1 2 3 4 points	
Diarrhea	0h <3h 3-7h 7-24h >24h<br>0 1 2 3 4 points	no mild Moderate very heavy<br>0 1 2 3 4 points	
Complexion	0h <3h 3-7h 7-24h >24h<br>0 1 2 3 4 points	no mild Moderate very heavy<br>0 1 2 3 4 points	
Stomachache	0h <3h 3-7h 7-24h >24h<br>0 1 2 3 4 points	no mild Moderate very heavy<br>0 1 2 3 4 points	
Blushing	0h <3h 3-7h 7-24h >24h<br>0 1 2 3 4 points	no mild Moderate very heavy<br>0 1 2 3 4 points	
Insomnia	0h <3h 3-7h 7-24h >24h<br>0 1 2 3 4 points	no mild Moderate very heavy<br>0 1 2 3 4 points	
Body pain	0h <3h 3-7h 7-24h >24h<br>0 1 2 3 4 points	no mild Moderate very heavy<br>0 1 2 3 4 points	
Depression	0h <3h 3-7h 7-24h >24h<br>0 1 2 3 4 points	no mild Moderate very heavy<br>0 1 2 3 4 points	
Irritable	0h <3h 3-7h 7-24h >24h<br>0 1 2 3 4 points	no mild Moderate very heavy<br>0 1 2 3 4 points	
Neuroticism	0h <3h 3-7h 7-24h >24h<br>0 1 2 3 4 points	no mild Moderate very heavy<br>0 1 2 3 4 points	
Total score			

Participant signature :_______Investigator's signature :__________Date :_____year_____month____day

Approval Number	Random number □□	Treatment number □□	Patient name phonetic alphabet □□□□		Page: stage of therapy (Week 12)

Observation table of TCM symptoms ofprimary dysmenorrhea

(Please fill in according to the latest menstrual cycle of dysmenorrhea)

Name		Age		Telephone	
Address		Married or not		First visit time	
Recently used what medicine		Pasthistory		Course of disease	
Menarche time			Menstrual days		
Pain time	Before menstruation day	□	Pain duration		
	Period - days	□			
Menstrual week Expect	Come on time	□	Menstrual color	Light red	□
	More than 7 days in advance	□		Bright red	□
	More than 7 days delay	□		Dark red	□
	From time to time	□			
Menstruation quantity	Normal	□	Menstruation quality	Normal	□
	Too much	□		Rarefied	□
	Less	□		Thick	□
Painful area	Middle abdomen		Nature ofpain	Cold pain	□
				Pain	□
	Lower abdomen on both sides	□		Dull pain	□
	Pain in the waist	□		Tingling	□
				Burning pain	□
Concomitant disease shape	Breast tenderness	□	lumbosacral pain	□	
	Anal bulge	□	Dizziness and tinnitus	□	
	Feel sick and vomit	□	Headache	□	
	Diarrhea	□			

Participant signature :_______Investigator's signature :______Date :_year___month_day

					Page：
**Approval Number**	**Random number** □□	**Treatment number** □□	**Patient name phonetic alphabet** □□□□		**stage of therapy (Week 12)**

Self-Rating Anxiety Scale (SAS)

According to your actual situation in the last month， tick a √ after the appropriate score.

Project	None or very little time	Small part time	Quite a bit of time	Most or all the time
1. I feel more nervous or anxious than usual	□ 1	□2	□ 3	□ 4
2. I am afraid for no reason	□ 1	□2	□ 3	□ 4
3. I am easily upset or panicked	□ 1	□2	□ 3	□ 4
4. I think I might be going crazy	□ 1	□2	□ 3	□ 4
5. I think everything is fine	□ 1	□2	□ 3	□ 4
6. My hands and feet tremble	□ 1	□2	□ 3	□ 4
7. I suffer from headaches, neck pain and back pain	□ 1	□2	□ 3	□ 4
8. I feel weak and tired easily	□ 1	□2	□ 3	□ 4
9. I feel calm and easy to sit still	□ 1	□2	□ 3	□ 4
10. I feel like my heart is beating fast	□ 1	□2	□ 3	□ 4
11. I was troubled by bouts of dizziness	□ 1	□2	□ 3	□ 4
12. I have an episode of fainting, or feel like I am going to pass out	□ 1	□2	□ 3	□ 4
13. I breathe in and out easily	□ 1	□2	□ 3	□ 4
14. My hands and feet are numb and tingling	□ 1	□2	□ 3	□ 4
15. I suffer from stomach pain and indigestion	□ 1	□2	□ 3	□ 4
16. I often have to urinate	□ 1	□2	□ 3	□ 4
17. My hands and feet are often dry and warm	□ 1	□2	□ 3	□ 4
18. My face is red and hot	□ 1	□2	□ 3	□ 4
19. I fall asleep easily and sleep well through the night	□ 1	□2	□ 3	□ 4
20. I have nightmares	□ 1	□2	□ 3	□ 4

Total score:|______| points

Investigator's signature: year month day

					Page:
**Approval Number**	**Random number** □□	**Treatment number** □□	**Patient name phonetic alphabet** □□□□		**stage of therapy (Week 12)**

Self-rating depressive scale(SDS)

According to your actual situation in the last month， tick a √ after the appropriate score.

Project	None or very little time	Small part time	Quite a bit of time	Most or all the time
1. I feel sullen and depressed	□ 1	□ 2	□ 3	□ 4
2. I think the morning is the best of the day	□ 1	□ 2	□ 3	□ 4
3. I burst into tears or wanted to cry	□ 1	□ 2	□ 3	□ 4
4. I think I might be going crazy	□ 1	□ 2	□ 3	□ 4
5. I eat as much as usual	□ 1	□ 2	□ 3	□ 4
6. I am as happy as ever when I am in close contact with the opposite sex	□ 1	□ 2	□ 3	□ 4
7. I notice my weight is dropping	□ 1	□ 2	□ 3	□ 4
8. I have trouble with constipation	□ 1	□ 2	□ 3	□ 4
9. My heart beats faster than usual	□ 1	□ 2	□ 3	□ 4
10. I feel tired for no reason	□ 1	□ 2	□ 3	□ 4
11. My mind is as clear as usual	□ 1	□ 2	□ 3	□ 4
12. I don’t find it difficult to do things that I do often	□ 1	□ 2	□ 3	□ 4
13. I feel restless and can’t calm down	□ 1	□ 2	□ 3	□ 4
14. I have hope for the future	□ 1	□ 2	□ 3	□ 4
15. I am more agitated than usual	□ 1	□ 2	□ 3	□ 4
16. I find it easy to make decisions	□ 1	□ 2	□ 3	□ 4
17. I feel like a useful person, someone needs me	□ 1	□ 2	□ 3	□ 4
18. My life is very interesting	□ 1	□ 2	□ 3	□ 4
19. I think others would be better off if I died	□ 1	□ 2	□ 3	□ 4
20. I am still interested in the things I am usually interested in	□ 1	□ 2	□ 3	□ 4

Total score:|______|_| points

Investigator's signature: year month day

					Page:
Approval Number	Random number □□	Treatment number □□	Patient name phonetic alphabet □□□□		stage of therapy (Week12)

Short form 36 questionnaire (SF-36)

According to your actual situation in the last month， tick a √ after the appropriate score.

1.Physiological function (PF: Physical Functioning)

(1)	Heavy physical activity (e.g. running, lifting heavy objects, strenuous exercise, etc.)	
	□There are a lot of restrictions □A little restriction	□No limit at all
(2)	Moderate activities (such as moving tables, sweeping the floor, doing exercises, etc.)	
	□There are a lot of restrictions □A little restriction	□No limit at all
(3)	Portable daily necessities (such as grocery shopping, shopping, etc.)	
	□There are a lot of restrictions □A little restriction	□No limit at all
(4)	Go up some stairs	
	□There are a lot of restrictions □A little restriction	□No limit at all
(5)	go up the stairs	
	□There are a lot of restrictions □A little restriction	□No limit at all
(6)	Bend over, bend knees, squat	
	□There are a lot of restrictions □A little restriction	□No limit at all
(7)	Walk about 1500 meters	
	□There are a lot of restrictions □A little restriction	□No limit at all
(8)	Walk about 800 meters	
	□There are a lot of restrictions □A little restriction	□No limit at all
(9)	Walk about 100 meters	
	□There are a lot of restrictions □A little restriction	□No limit at all
(10)	bathe and dress yourself	
	□There are a lot of restrictions □A little restriction	□No restrictions at all

A lot of restrictions A little restriction

					Page:
Approval Number	Random number □□	Treatment number □□	Patient name phonetic alphabet □□□□		stage of therapy (Week 12)

2.Physiological function

(13)Reduced time for work or other activities

□Yes □No

(14)Only part of what I wanted to do

□Yes □No

(15)Restricted types of work or activities you want to do

□Yes □No

(16)Difficulty completing work or other activities (eg, requiring extra effort)

□Yes □No

3.body pain

（7） Have you had any physical pain in the past four weeks ?

□No pain at □All There is very little pain □Have mild pain

□Moderate pain □Severe pain □Very severe pain

（8） In the past four weeks , has your physical pain interfered with your normal work (including work and household activities) ?

□No effect at all □Have a little influence □Moderate impact

□Have a greater impact □Have great influence

4.general health

（16） In general , Your health is

□very good □ Well □Good □ Fair □Poor

（17） I seem to get sick more easily than others

□Absolutely correct □Mostly correct □Not sure □Mostly wrong □Absolutely wrong

（18） I'm as healthy as anyone I know

□Absolutely correct □Mostly correct □ Not sure □ Mostly wrong □Bsolutely wrong

（19） I think my health is getting worse

□Absolutely correct □ Mostly correct □ Not sure □ Mostly wrong□Absolutely wrong

					Page:
Approval Number	Random number □□	Treatment number □□	Patient name phonetic alphabet □□□□		stage of therapy (Week 12)

（20） My health is very good

☑Absolutely correct □Mostly correct □Not sure □Mostly wrong □Absolutely wrong

11. Energy

（13） Do you feel life is full?

□All the time □Most of the time □More time

□Part of the time □Don't have this feeling

（14） Are you energetic?

□All the time □Most of the time □More time

□Part of the time □Don't have this feeling

（15） Are you feeling exhausted?

□All the time □Most of the time □More time

□Part of the time □Don't have this feeling

（16） Are you feeling tired?

□All the time □Most of the time □More time

□Part of the time □Don't have this feeling

12. Social function

（7） In the past four weeks, to what extent has your poor physical health or mood interfered with your normal social activities with family , friends, neighbors or groups ?

□No impact at all □Slight impact □Moderate impact

□Influence □Great influence

（8） your health limit your social activities (such as visiting family and friends) ?

□All the time □Most of the time □More time

□Part of the time □Don't have this feeling

7.emotional function

（1）Reduced time for work or other activities

□Yes □No

（2）Only part of what I wanted to do

□Yes □No

Approval Number	Random number □□	Treatment number □□	Patient name phonetic alphabet □□□□		stage of therapy (Week 12)

（3）Doing work or other activities less carefully than usual

□Yes □No

1 . Mental Health

（16） Are you a nervous person?

□All the time □Most of the time □More time

□part of the time □Don't have this feeling

（17） Are you feeling down and nothing can cheer you up?

□All the time □Most of the time □More time

□Part of the time □Don't have this feeling

（18） Do you feel calm?

□All the time □Most of the time □More time

□part of the time □Don't have this feeling

（19） Are you feeling down?

□All the time □Most of the time □More time

□Part of the time □Don't have this feeling

（20） Are you a happy person?

□All the time □Most of the time □More time

□part of the time □Don't have this feeling

Participant Signature :_____________ Date :_____year___month___day

Investigator Signature : ___________ Date:______year___month___day

Approval Number	Random number □□	Treatment number □□	Patient name phonetic alphabet □□□□		stage of therapy (Week 12)

Routine blood test

Date of examination：20|_|_|year|_|_|month|_|_|day							
Index	measured value	unit	Clinical significance judgment				remarks
			1	2	3	4	
Red-cell count（RBC）			□	□	□	□	
Leucocyte count（WBC）			□	□	□	□	
Platelet count（PLT）			□	□	□	□	
Hematocrit（HCT）			□	□	□	□	
Neutrophil count was performed（NEUT)			□	□	□	□	
Hemoglobin(Hb)			□	□	□	□	
**Note: Clinical significance judgment: 1 normal 2 abnormal no clinical significance 3 abnormal clinical significance 4 not checked**							

Blood biochemical

Date of examination：20|_|_|year|_|_|month|_|_|day							
Index	measured value	unit	Clinical significance judgment				remarks
			1	2	3	4	
Alanine aminotransferase（ALT)			□	□	□	□	
Aspartate aminotransferase (AST）			□	□	□	□	
Alkaline phosphatase（ALP）			□	□	□	□	
Total bilirubin（TBIL)			□	□	□	□	
Bilirubin direct(DBIL)			□	□	□	□	
Total protein (TP)			□	□	□	□	
Serum creatinine(SCr)			□	□	□	□	
Purine trione(UA)			□	□	□	□	
Fasting blood-glucose（FPG）			□	□	□	□	
Potassium（K）			□	□	□	□	
Sodium（Na）			□	□	□	□	
Chlorine（Cl）			□	□	□	□	
Calcium（Ca）			□	□	□	□	
**Note: Clinical significance judgment: 1 normal 2 abnormal no clinical significance 3 abnormal clinical significance 4 not checked**							

Investigator signature： **Date：**

Approval Number	Random number □□	Treatment number □□	Patient name phonetic alphabet □□□□		Page：<br>stage of therapy (Week 12)

Routine urine test

Date of examination：20|_|_|year|_|_|month|_|_|day							
Inde x	Measured value	Unit	Clinical significance judgment				Rem arks
			1	2	3	4	
Urinary red blood cells（ERY)			□	□	□	□	
Urinary white blood cells (LEU）			□	□	□	□	
Urine protein （PRO）			□	□	□	□	
Urine glucose（GLU)			□	□	□	□	
Urine acetone bodies (KET)			□	□	□	□	
**Note: Clinical significance judgment: 1 normal 2 abnormal no clinical significance 3 abnormal clinical significance 4 not checked**							

Expectation value evaluation

Content: Generally speaking, do you think electroacupuncture has an effect in treating primary dysmenorrhea?

☐yes □no □**d**imness

Do you think that acupuncture will be helpful in improving your PD?

☐yes □no □**d**imness

Blind method evaluation

Inform the patient that there are two kinds of electroacupuncture therapy: one is traditional electroacupuncture, stabbed deep; the other is microelectroacupuncture, stabbed shallow; ask whether the patient receives traditional electroacupuncture therapy?

☑Hear nothing of□Yes□No

Overall evaluation of patient self-efficacy（PGI-I grade）

Selection best describes the changes in the symptoms of dysmenorrhea compared to the trial:□1 A lot of improvement; **□2 Moderate improvement:□3 Get better;☐4 unchanged;□5 Increase a little; ☐6 Moderate exacerbation □7 It lot very aggravation.**

Subjects signed: Date： year month day

Investigator signature： Date： year month day

					Page：
Approval Number	Random number □□	Treatment number □□	Patient name phonetic alphabet □□□□		stage of therapy (Week 24)

Laboratory checklist paste place

					Page：
**Approval Number**	**Random number** □□	**Treatment number** □□	**Patient name phonetic alphabet** □□□□		**stage of therapy (Week 24)**

Visual Analogue Scale (VAS)

This scale is a scale line from the length of the 0-10 cm line, right (0 cm) painless and right (10 cm) of the "most serious pain", in the middle of different degrees of pain, please according to the past three menstrual cycle dysmenorrhea in the most can represent their pain to draw a cross line, select the highest value as the effective data.

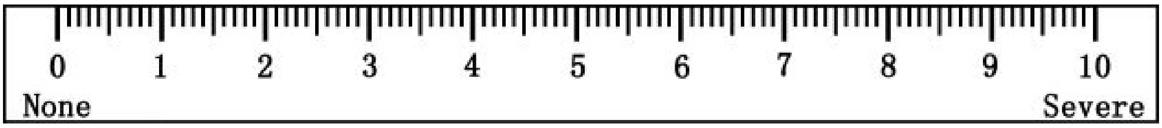

0 points: no pain;

1-3 points: mild pain, daily life and work are not affected;

4 to 6 points: moderate pain, daily life and work are affected;

7-9 points: severe pain, daily life and work and sleep are affected;

10 points: severe pain

Fill in for reference

Participant Signature :________________ Date :_____year___month___day

Investigator Signature :_______________ Date :_____year___month__day

					Page：
**Approval Number**	**Random number** □□	**Treatment number** □□	**Patient name phonetic alphabet** □□□□		**stage of therapy (Week 24)**

Numeric Pain Rating Scale（NRS）

The intensity of the current, best, and worst pain levels is expressed from 0 (no pain) to 10 (worst pain). Please rate your pain according on your latest menstrual cycle.

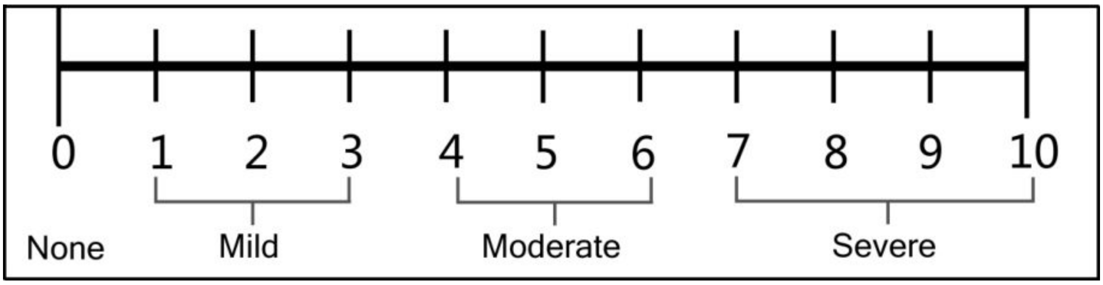

Participant Signature : ____________________Date : ____Year ____month ___day

Investigator Signature :___________________Date :____Year ____month ___day

					Page：
Approval Number	Random number □□	Treatment number □□	Patient name phonetic alphabet □□□□		stage of therapy (Week 24)

COX Menstrual Symptom Scale (CMSS)

(Please fill in according to the last menstrual cycle)

symptom	Duration (√)	Severity (√)	Score
Lower abdominal pain	0h <3h 3-7h 7-24h >24h<br>0 1 2 3 4 points	no mild Moderate very heavy<br>0 1 2 3 4 points	
Nausea	0h <3h 3-7h 7-24h >24h<br>0 1 2 3 4 points	no mild Moderate very heavy<br>0 1 2 3 4 points	
Vomit	0h <3h 3-7h 7-24h >24h<br>0 1 2 3 4 points	no mild Moderate very heavy<br>0 1 2 3 4 points	
Loss of appetite	0h <3h 3-7h 7-24h >24h<br>0 1 2 3 4 points	no mild Moderate very heavy<br>0 1 2 3 4 points	
Headache	0h <3h 3-7h 7-24h >24h<br>0 1 2 3 4 points	no mild Moderate very heavy<br>0 1 2 3 4 points	
Back (lumbosacral) Pain	0h <3h 3-7h 7-24h >24h<br>0 1 2 3 4 points	no mild Moderate very heavy<br>0 1 2 3 4 points	
Leg pain	0h <3h 3-7h 7-24h >24h<br>0 1 2 3 4 points	no mild Moderate very heavy<br>0 1 2 3 4 points	
Fatigue	0h <3h 3-7h 7-24h >24h<br>0 1 2 3 4 points	no mild Moderate very heavy<br>0 1 2 3 4 points	
Dizziness	0h <3h 3-7h 7-24h >24h<br>0 1 2 3 4 points	no mild Moderate very heavy<br>0 1 2 3 4 points	
Diarrhea	0h <3h 3-7h 7-24h >24h<br>0 1 2 3 4 points	no mild Moderate very heavy<br>0 1 2 3 4 points	
Complexion	0h <3h 3-7h 7-24h >24h<br>0 1 2 3 4 points	no mild Moderate very heavy<br>0 1 2 3 4 points	
Stomachache	0h <3h 3-7h 7-24h >24h<br>0 1 2 3 4 points	no mild Moderate very heavy<br>0 1 2 3 4 points	
Blushing	0h <3h 3-7h 7-24h >24h<br>0 1 2 3 4 points	no mild Moderate very heavy<br>0 1 2 3 4 points	
Insomnia	0h <3h 3-7h 7-24h >24h<br>0 1 2 3 4 points	no mild Moderate very heavy<br>0 1 2 3 4 points	
Body pain	0h <3h 3-7h 7-24h >24h<br>0 1 2 3 4 points	no mild Moderate very heavy<br>0 1 2 3 4 points	
Depression	0h <3h 3-7h 7-24h >24h<br>0 1 2 3 4 points	no mild Moderate very heavy<br>0 1 2 3 4 points	
Irritable	0h <3h 3-7h 7-24h >24h<br>0 1 2 3 4 points	no mild Moderate very heavy<br>0 1 2 3 4 points	
Neuroticism	0h <3h 3-7h 7-24h >24h<br>0 1 2 3 4 points	no mild Moderate very heavy<br>0 1 2 3 4 points	
Total score			

Participant signature :_______Investigator's signature :__________Date :_____year_____month____day

					Page:
**Approval Number**	**Random number** □□	**Treatment number** □□	**Patient name phonetic alphabet** □□□□		**stage of therapy (Week 24)**

Observation table of TCM symptoms ofprimary dysmenorrhea

(Please fill in according to the latest menstrual cycle of dysmenorrhea)

Name		Age		Telephone	
Address		Married or not		First visit time	
Recently used what medicine		Pasthistory		Course of disease	
Menarche time			Menstrual days		
Pain time	Before menstruation day	□	Pain duration		
	Period - days	□			
Menstrual week Expect	Come on time	□	Menstrual color	Light red	□
	More than 7 days in advance	□		Bright red	□
	More than 7 days delay	□		Dark red	□
	From time to time	□			
Menstruation quantity	Normal	□	Menstruation quality	Normal	□
	Too much	□		Rarefied	□
	Less	□		Thick	□
Painful area	Middle abdomen		Nature ofpain	Cold pain	□
				Pain	□
	Lower abdomen on both sides	□		Dull pain	□
	Pain in the waist	□		Tingling	□
				Burning pain	□
Concomitant disease shape	Breast tenderness	□	lumbosacral pain	□	
	Anal bulge	□	Dizziness and tinnitus	□	
	Feel sick and vomit	□	Headache	□	
	Diarrhea	□			

Participant signature :_______Investigator's signature :______Date :_year___month_day

					Page:
**Approval Number**	**Random number** □□	**Treatment number** □□	**Patient name phonetic alphabet** □□□□		**stage of therapy (Week 24)**

Self-Rating Anxiety Scale (SAS)

According to your actual situation in the last month， tick a √ after the appropriate score.

Project	None or very little time	Small part time	Quite a bit of time	Most or all the time
1. I feel more nervous or anxious than usual	□ 1	□2	□ 3	□ 4
2. I am afraid for no reason	□ 1	□2	□ 3	□ 4
3. I am easily upset or panicked	□ 1	□2	□ 3	□ 4
4. I think I might be going crazy	□ 1	□2	□ 3	□ 4
5. I think everything is fine	□ 1	□2	□ 3	□ 4
6. My hands and feet tremble	□ 1	□2	□ 3	□ 4
7. I suffer from headaches, neck pain and back pain	□ 1	□2	□ 3	□ 4
8. I feel weak and tired easily	□ 1	□2	□ 3	□ 4
9. I feel calm and easy to sit still	□ 1	□2	□ 3	□ 4
10. I feel like my heart is beating fast	□ 1	□2	□ 3	□ 4
11. I was troubled by bouts of dizziness	□ 1	□2	□ 3	□ 4
12. I have an episode of fainting, or feel like I am going to pass out	□ 1	□2	□ 3	□ 4
13. I breathe in and out easily	□ 1	□2	□ 3	□ 4
14. My hands and feet are numb and tingling	□ 1	□2	□ 3	□ 4
15. I suffer from stomach pain and indigestion	□ 1	□2	□ 3	□ 4
16. I often have to urinate	□ 1	□2	□ 3	□ 4
17. My hands and feet are often dry and warm	□ 1	□2	□ 3	□ 4
18. My face is red and hot	□ 1	□2	□ 3	□ 4
19. I fall asleep easily and sleep well through the night	□ 1	□2	□ 3	□ 4
20. I have nightmares	□ 1	□2	□ 3	□ 4

Total score:|______| points

Investigator's signature: year month day

					Page：
**Approval Number**	**Random number** □□	**Treatment number** □□	**Patient name phonetic alphabet** □□□□		**stage of therapy (Week 24)**

Self-rating depressive scale(SDS)

According to your actual situation in the last month， tick a √ after the appropriate score.

Project	None or very little time	Small part time	Quite a bit of time	Most or all the time
1. I feel sullen and depressed	□ 1	□ 2	□ 3	□ 4
2. I think the morning is the best of the day	□ 1	□ 2	□ 3	□ 4
3. I burst into tears or wanted to cry	□ 1	□ 2	□ 3	□ 4
4. I think I might be going crazy	□ 1	□ 2	□ 3	□ 4
5. I eat as much as usual	□ 1	□ 2	□ 3	□ 4
6. I am as happy as ever when I am in close contact with the opposite sex	□ 1	□ 2	□ 3	□ 4
7. I notice my weight is dropping	□ 1	□ 2	□ 3	□ 4
8. I have trouble with constipation	□ 1	□ 2	□ 3	□ 4
9. My heart beats faster than usual	□ 1	□ 2	□ 3	□ 4
10. I feel tired for no reason	□ 1	□ 2	□ 3	□ 4
11. My mind is as clear as usual	□ 1	□ 2	□ 3	□ 4
12. I don’t find it difficult to do things that I do often	□ 1	□ 2	□ 3	□ 4
13. I feel restless and can’t calm down	□ 1	□ 2	□ 3	□ 4
14. I have hope for the future	□ 1	□ 2	□ 3	□ 4
15. I am more agitated than usual	□ 1	□ 2	□ 3	□ 4
16. I find it easy to make decisions	□ 1	□ 2	□ 3	□ 4
17. I feel like a useful person, someone needs me	□ 1	□ 2	□ 3	□ 4
18. My life is very interesting	□ 1	□ 2	□ 3	□ 4
19. I think others would be better off if I died	□ 1	□ 2	□ 3	□ 4
20. I am still interested in the things I am usually interested in	□ 1	□ 2	□ 3	□ 4

Total score:|______|_| points

Investigator's signature: year month day

					Page：
Approval Number	Random number □□	Treatment number □□	Patient name phonetic alphabet □□□□		stage of therapy (Week 24)

Short form 36 questionnaire (SF-36)

According to your actual situation in the last month， tick a √ after the appropriate score.

1.Physiological function (PF: Physical Functioning)

(1) Heavy physical activity (e.g. running, lifting heavy objects, strenuous exercise, etc.)

□There are a lot of restrictions □A little restriction □No limit at all

(2) Moderate activities (such as moving tables, sweeping the floor, doing exercises, etc.)

□There are a lot of restrictions □A little restriction □No limit at all

(3) Portable daily necessities (such as grocery shopping, shopping, etc.)

□There are a lot of restrictions □A little restriction □No limit at all

(4) Go up some stairs

□There are a lot of restrictions □A little restriction □No limit at all

(5) go up the stairs

□There are a lot of restrictions □A little restriction □No limit at all

(6) Bend over, bend knees, squat

□There are a lot of restrictions □A little restriction □No limit at all

(7) Walk about 1500 meters

□There are a lot of restrictions □A little restriction □No limit at all

(8) Walk about 800 meters

□There are a lot of restrictions □A little restriction □No limit at all

(9) Walk about 100 meters

□There are a lot of restrictions □A little restriction □No limit at all

(10) bathe and dress yourself

□There are a lot of restrictions □A little restriction □No restrictions at all

A lot of restrictions A little restriction

					Page:
**Approval Number**	**Random number** □□	**Treatment number** □□	**Patient name phonetic alphabet** □□□□		**stage of therapy (Week 24)**

2.Physiological function

(17)Reduced time for work or other activities

□Yes □No

(18)Only part of what I wanted to do

□Yes □No

(19)Restricted types of work or activities you want to do

□Yes □No

(20)Difficulty completing work or other activities (eg, requiring extra effort)

□Yes □No

3.body pain

（9） Have you had any physical pain in the past four weeks ?

□No pain at □All There is very little pain □Have mild pain

□Moderate pain □Severe pain □Very severe pain

（10） In the past four weeks , has your physical pain interfered with your normal work (including work and household activities) ?

□No effect at all □Have a little influence □Moderate impact

□Have a greater impact □Have great influence

4.general health

（21） In general , Your health is

□very good □ Well □Good □ Fair □Poor

（22） I seem to get sick more easily than others

□Absolutely correct □Mostly correct □Not sure □Mostly wrong □Absolutely wrong

（23） I'm as healthy as anyone I know

□Absolutely correct □Mostly correct □ Not sure □ Mostly wrong □Bsolutely wrong

（24） I think my health is getting worse

□Absolutely correct □ Mostly correct □ Not sure □ Mostly wrong□Absolutely wrong

					Page：
Approval Number	Random number □□	Treatment number □□	Patient name phonetic alphabet □□□□		stage of therapy (Week2 4)

（25） My health is very good

☑Absolutely correct □Mostly correct □Not sure □Mostly wrong □Absolutely wrong

13. Energy

（17） Do you feel life is full?

□All the time □Most of the time □More time

□Part of the time □Don't have this feeling

（18） Are you energetic?

□All the time □Most of the time □More time

□Part of the time □Don't have this feeling

（19） Are you feeling exhausted?

□All the time □Most of the time □More time

□Part of the time □Don't have this feeling

（20） Are you feeling tired?

□All the time □Most of the time □More time

□Part of the time □Don't have this feeling

14. Social function

（9） In the past four weeks, to what extent has your poor physical health or mood interfered with your normal social activities with family , friends, neighbors or groups ?

□No impact at all □Slight impact □Moderate impact

□Influence □Great influence

（10） your health limit your social activities (such as visiting family and friends) ?

□All the time □Most of the time □More time

□Part of the time □Don't have this feeling

7.emotional function

（1）Reduced time for work or other activities

□Yes □No

（2）Only part of what I wanted to do

□Yes □No

					Page:
**Approval Number**	**Random number** □□	**Treatment number** □□	**Patient name phonetic alphabet** □□□□		**stage of therapy (Week 24)**

（3）Doing work or other activities less carefully than usual

□Yes □No

1 . Mental Health

（21） Are you a nervous person?

□All the time □Most of the time □More time

□part of the time □Don't have this feeling

（22） Are you feeling down and nothing can cheer you up?

□All the time □Most of the time □More time

□Part of the time □Don't have this feeling

（23） Do you feel calm?

□All the time □Most of the time □More time

□part of the time □Don't have this feeling

（24） Are you feeling down?

□All the time □Most of the time □More time

□Part of the time □Don't have this feeling

（25） Are you a happy person?

□All the time □Most of the time □More time

□part of the time □Don't have this feeling

Participant Signature :_____________ Date :_____year___month___day

Investigator Signature : ___________ Date:______year___month___day

					Page：
Approval Number	Random number<br>□□	Treatment number<br>□□	Patient name phonetic alphabet<br>□□□□		stage of therapy (Week 24)

Overall Evaluation of Patient Self-efficacy (PGI-I score)

Selection best describes the changes in the symptoms of dysmenorrhea compared to the trial:
□1 A lot of improvement □2 Moderate improvement; □3 Get better; □4 unchanged;□5 Increase a little; □ 6 Moderate exacerbation; □ 7 It lot very aggravation.

Participant Signature :_________ Date :_____year___month___day

Investigator Signature : ___________ Date:_year_month___day

					Page:
Approval Number	Random number □□	Treatment number □□	Patient name phonetic alphabet □□□□		Experimental summary

Summary of the completion of the test

Date of last treatment of patient: |_|_|/|_|_|/|_|_|_|_| month day year

Were there any adverse events during the trial in this patient? Yes _ No □

If there are adverse reactions, have they been resolved? Yes _ No □

If no, adverse reactions should be monitored until stable or resolved. Yes _ No □

Is the patient in24 weeks ±3Clinical trials completed within days? Yes _ No □

If no, please fill in the following items :

Patient discontinued trial on |_|_|/|_|_|/|_|_|_|_| month day year

The first to propose to discontinue the test is: (choose one)

Patient .... □

Trial investigator.. .... □

Other .... □

If other, please specify____________________________

The main reasons for discontinuing the trial were : (choose one)

Adverse events (completed the adverse event table) .... □

Lack of efficacy.. □

Violation of the test plan .... □

Lost to follow-up .... □

Suspended by the researcher .... □

Other reasons :___________________________________

Participant Signature :__________________ Date :________year___month___day

Investigator Signature :_________________ Date :________year___month___day

					Page _
Approval Number	Random Number □□	Treatment number □□	Patient name phonetic alphabet □□□□		Emergency medication

Emergency Medication (CONCOMITANT MEDICATION)

Trade name or generic	Preparation name	Use the original	Dose	Start date	End date
				year month day	year month day
				year month day	year month day
				year month day	year month day
				year month day	year month day
				year month day	year month day
				year month day	year month day
				year month day	year month day
				year month day	year month day
				year month day	year month day
				year month day	year month day
				year month day	year month day
				year month day	year month day
				year month day	year month day
				year month day	year month day
				year month day	year month day
				year month day	year month day
				year month day	year month day

Investigator Signature :_______________ Date :_____year___month___day

					Page:
License number	Random number □□	Treatment number □□	Patient name of pinyin letter □□□□		**Concomitant treatment**

omitant treatment table

With or without concomitant treatment： □NO □YES，Please continue to complete the form below			
Treatment name	Date commenced（Year/Mouth/Day）	Whether to continue（Year/Mouth/Day)	The cause of reatment
		□No，Deadline： □Yes	
		□No，Deadline： □Yes	
		□No，Deadline： □Yes	
		□No，Deadline： □Yes	
		□No，Deadline： □Yes	
		□No，Deadline： □Yes	
		□No，Deadline： □Yes	
		□No，Deadline： □Yes	
		□No，Deadline： □Yes	
		□No，Deadline： □Yes	
		□No，Deadline： □Yes	
		□No，Deadline： □Yes	
		□No，Deadline： □Yes	
		□No，Deadline： □Yes	
		□No，Deadline： □Yes	
		□No，Deadline： □Yes	
		□No，Deadline： □Yes	
The researcher signed		**Date ：**	

					Page:
License number	Random number □□	Treatment number □□	Patient name of pinyin letter □□□□		**Concomitant treatment**

Electroacupuncture-related safety evaluation (recorded at any time)□0 NO；□1 Yes，Please continue to complete the form below（Only for patients receiving electroand modern electroacupuncture）	
symptom code/ name* （01-13 use）	Time of symptom onset（Please fill in 1-12 week，Time 1-7)
|__|__|	Week |, | | treatment
|__|__|	Week |, | | treatment
|__|__|	Week |, | | treatment
|__|__|	Week |, | | treatment
|__|__|	Week |, | | treatment
|__|__|	Week |, | | treatment
|__|__|	Week |, | | treatment
|__|__|	Week |, | | treatment
|__|__|	Week |, | | treatment
|__|__|	Week |, | | treatment
|__|__|	Week |, | | treatment
|__|__|	Week |, | | treatment
*symptom code ：01= Broken needle; 02= dizzy needle; 03= local subcutaneous bleeding; 04= local infection; 05= unbearable acupuncture pain (acupuncture pain is VAS 8); 06= nausea; 07= vomiting; 08= palpitations; 09= dizziness; 10= headache; 11= anorexia; 12= insomnia; 13= other,name：__________________________	

					Page _
**Approval Number**	**Random Number** □□	**Treatment number** □□	**Patient name phonetic alphabet** □□□□		**Adverse event**

ADVERSE EXPERIENCES

Record (in standard medical terms) all observed and direct-inquiry-derived adverse events with the question "How have you felt different since your last examination?" Try to use diagnostic names instead of symptom names. One adverse event is recorded in each column. If there is no Good things happen, please click here □ hit " ” and sign below this form.

**Adverse event name** (fill in the handwriting to be clear)			
**Start date and time**	|_|_|/|_|_|/|_|_|_|_| month day year	|_|_|/|_|_|/|_|_|_|_| month day year	|_|_|/|_|_|/|_|_|_|_| month day year
**End date and time** (If the adverse event persists, please do not complete this item)	|_|_|/|_|_|/|_|_|_|_| month day year	|_|_|/|_|_|/|_|_|_|_| month day year	|_|_|/|_|_|/|_|_|_|_| month day year
**Characteristics of Adverse Events**			
**Adverse event severity**	□ Level 1 □ Level 2 □ 3 Level □ 4 class		
**The measures taken**			
**Post-processing situation**			
**Whether the patient withdrawal from the trial due to this adverse event?**	□ Yes □ No	□ Yes □ No	□ Yes □ No

Investigator Signature:________________________ Date:________________

Other Instructions

					Page:
License number	Random number □□	Treatment number □□	Patient name of pinyin letter □□□□		Serious adverse events

Serious Adverse Event Report Form（SAE）

Whether there are serious adverse events？ □NO □ YES If so, please complete the form below

Reporter of serious adverse events<br><br>(Please write clearly)	
Report time	|__|__||__|__|year|__||month|__|_____|day
Serious Adverse Event Name(Clear handwriting)	
The doctor is informed of the SAE time	|__|__||__|__|year|__||year|__||day|__|__|hour| ||minute（24 Hour system）
SAE occurrence time	|__|__||__|__|year|__||year|__||day|__|__|hour| ||minute（24 Hour system）
SAE characteristic	□ Symptoms or signs are paroxysmal (number of attacks：|_|_|) □ The symptoms or signs were persisten<br><br>□ Continuous laboratory indicators were abnormal□ Temporary laboratory indicators were abnormal<br><br>other ___________
SAE circumstances	□ Lead to hospitalization □ Extend the length of stay □ permanent disability □Functional disorder □ Influence working ability<br><br>□ Causes congenital malformations □ Life-threatening or death □Other_
For the test medication is taken measure	□No action is taken □Temporary interruption □Permanent stop of study treatment □Increase the combination of drug therapy □Non-pharmacological treatment was administered<br><br>□other_______________
Relationship with the test drugs	□Certainly related □Probably about □Certainly irrelevant□It may not matter □Unidentified
SAE lapse to	□ Symptoms disappear（sequel：□ NO□ YES，Expression：_____） □ Symptoms continue<br><br>□ death（death time：| | || | |Year| | |Mouth| | |day） □ other
Date and time of symptom disappearance(If Symptoms still exist, do not fill in this column)	|_ | || | ||Year| | ||Mouth| | |day| | |Hour| | |minute（24 Hour system）

Whether this patient was severely not because of this Good event exit trial？	☐ No ☐ Yes
SAE procedure description（Including symptoms, signs, laboratory tests, etc）And the details of the handling：<br><br>**Investigator signature：** **Date ：**	

License number	Random number □□	Treatment number □□	Patient name of pinyin letter □□□□		Evaluation of compliance

Compliance with compliance evaluation

The number of treatment	4week	8week	12week
Number of planned treatments			
Actual number of treatments			
Total number of treatments is planned			
Total number of actual treatments			

Investigator signature： **Date： year month day**

					Page _
**Approval Number**	**Random number** □□	**Treatment number** □□□	**Patient name phonetic alphabet** □□□□		**medical records**

Brief medical record

Observing physician:

Date :_______year___month___day

					Page _
**Approval Number**	**random number** □□	**Treatment number** □□	**Patient name phonetic alphabet** □□□□		**CRF Audit**

CRF Audit Statement

Myself________________, Head of Clinical Trials , here by declares , The records of all items in this case report form have breviewed to be true、complete and accurate .

Test Leader **Signature** ________**Date** ________
